# Effectiveness of a live oral human rotavirus vaccine after programmatic introduction in Bangladesh: A cluster-randomized trial

**DOI:** 10.1371/journal.pmed.1002282

**Published:** 2017-04-18

**Authors:** K. Zaman, David A. Sack, Kathleen M. Neuzil, Mohammad Yunus, Lawrence H. Moulton, Jonathan D. Sugimoto, Jessica A. Fleming, Ilias Hossain, Shams El Arifeen, Tasnim Azim, Mustafizur Rahman, Kristen D. C. Lewis, Andrea J. Feller, Firdausi Qadri, M. Elizabeth Halloran, Alejandro Cravioto, John C. Victor

**Affiliations:** 1 International Centre for Diarrhoeal Disease Research, Bangladesh, Dhaka, Bangladesh; 2 Johns Hopkins Bloomberg School of Public Health, Baltimore, Maryland, United States of America; 3 PATH, Seattle, Washington, United States of America; 4 Vaccine and Infectious Disease Division, Fred Hutchinson Cancer Research Center, Seattle, Washington, United States of America; 5 Biostatistics Department, University of Washington, Seattle, Washington, United States of America; Makerere University Medical School, UGANDA

## Abstract

**Background:**

Rotavirus vaccines are now globally recommended by the World Health Organization (WHO), but in early 2009 WHO’s Strategic Advisory Group of Experts on Immunization reviewed available data and concluded that there was no evidence for the efficacy or effectiveness of a two-dose schedule of the human rotavirus vaccine (HRV; Rotarix) given early at 6 and 10 wk of age. Additionally, the effectiveness of programmatic rotavirus vaccination, including possible indirect effects, has not been assessed in low-resource populations in Asia.

**Methods and findings:**

In Bangladesh, we cluster-randomized (1:1) 142 villages of the Matlab Health and Demographic Surveillance System to include two doses of HRV with the standard infant vaccines at 6 and 10 wk of age or to provide standard infant vaccines without HRV. The study was initiated November 1, 2008, and surveillance was conducted concurrently at Matlab Diarrhoea Hospital and two community treatment centers to identify children less than 2 y of age presenting with acute rotavirus diarrhea (ARD) through March 31, 2011. Laboratory confirmation was made by enzyme immunoassay detection of rotavirus antigen in stool specimens. Overall effectiveness of the HRV vaccination program (primary objective) was measured by comparing the incidence rate of ARD among all children age-eligible for vaccination in villages where HRV was introduced to that among such children in villages where HRV was not introduced. Total effectiveness among vaccinees and indirect effectiveness were also evaluated. In all, 6,527 infants were age-eligible for vaccination in 71 HRV villages, and 5,791 in 71 non-HRV villages. In HRV villages, 4,808 (73.7%) infants received at least one dose of HRV. The incidence rate of ARD was 4.10 cases per 100 person-years in non-HRV villages compared to 2.8 per 100 person-years in HRV villages, indicating an overall effectiveness of 29.0% (95% CI, 11.3% to 43.1%). The total effectiveness of HRV against ARD among vaccinees was 41.4% (95% CI, 23.2% to 55.2%). The point estimate for total effectiveness was higher against ARD during the first year of life than during the second (45.2% versus 28.9%), but estimates for the second year of life lacked precision and did not reach statistical significance. Indirect effects were not detected. To check for bias in presentation to treatment facilities, we evaluated the effectiveness of HRV against acute diarrhea associated with enterotoxigenic *Escherichia coli*; it was 4.0% (95% CI, −46.5% to 37.1%), indicating that bias likely was not introduced. Thirteen serious adverse events were identified among recipients of HRV, but none were considered related to receipt of study vaccine. The main limitation of this study is that it was an open-label study with an observed-only control group (no placebo).

**Conclusions:**

The two-dose HRV rotavirus vaccination program significantly reduced medically attended ARD in this low-resource population in Asia. Protection among vaccinees was similar to that in other low-resource settings. In low-resource populations with high rotavirus incidence, large-scale vaccination across a wide population may be required to obtain the full benefit of rotavirus vaccination, including indirect effects.

**Trial registration:**

ClinicalTrials.gov NCT00737503

## Abstract 2

John Victor and colleagues study protection against acute rotavirus diarrhea by a two-dose rotavirus vaccination regimen in Matlab, Bangladesh.

## Author summary

**Why was this study done?:**

- Global diarrheal deaths among children under five years of age in 2013 were estimated at 575,594, of which well over a third (37.3%) were estimated to be due to rotavirus, mostly in low- and middle-income countries in Africa and Asia.
- The efficacy of the two-dose human rotavirus vaccine (HRV) on the schedule currently recommended by the World Health Organization had not been evaluated in a low-resource population in Asia in a prospective trial.
- Individual-randomized efficacy trials and observational (case–control) effectiveness studies cannot evaluate the various population-level effects, including indirect effects, of new rotavirus vaccination programs.

**What did the researchers do and find?:**

- In rural Bangladesh, 142 villages were randomly allocated to introduction of a rotavirus vaccination program with two doses of HRV (given at six and ten weeks of age) or routine infant vaccines already part of the Bangladesh infant immunization schedule.
- Diarrheal surveillance among all children under two years of age was conducted in the local diarrhea hospital and two community treatment centers to identify those presenting with laboratory-confirmed acute rotavirus diarrhea.
- Approximately 70% of infants age-eligible for receipt of HRV received at least one dose. The vaccination program reduced presentations to the treatment facilities due to rotavirus diarrhea by nearly one-third among the entire population of age-eligible infants, regardless of receipt of HRV.
- Among infants receiving two doses of HRV, presentations to the treatment facilities due to rotavirus diarrhea were reduced by 41%.
- Infants not receiving HRV in villages where HRV was introduced did not appear to receive any indirect protection in this study.

**What do these findings mean?:**

- HRV was moderately effective in protecting those vaccinated against acute rotavirus diarrhea.
- In this low-resource population in Asia, HRV offered protection that was comparable to that seen with this vaccine in Africa and to that of other live attenuated rotavirus vaccines studied in similar populations.
- Large-scale and sustained vaccination across a wider population in such settings may be required to achieve substantial reductions in the force of infection of rotavirus that would result in indirect benefits.

## Introduction

Diarrhea continues to be a leading killer of children in low- and middle-income countries, and rotavirus has been shown to be the most common cause of moderate-to-severe diarrhea in infants and young children worldwide [1,2]. In 2005, the World Health Organization (WHO) Strategic Advisory Group of Experts on Immunization called for the generation of efficacy data for currently available live oral rotavirus vaccines in populations with high child mortality in Africa and Asia [3]. While rotavirus vaccines were demonstrated to be safe and efficacious against severe rotavirus disease [4–6], outstanding questions remained at the time the current study was planned.

In Africa, the human rotavirus vaccine (HRV, Rotarix) was not evaluated in prospective trials on the schedule currently recommended by WHO at 6 wk (with diphtheria-tetanus-pertussis [DTP] 1) and 10 wk (with DTP2) of age [7]. Studies have shown that live oral rotavirus vaccine immunogenicity is reduced when the vaccine is given concomitantly with oral poliovirus vaccine (OPV) and might be affected most by the first dose of OPV, given at the same time as DTP1 [8,9]. Additionally, immune responses to vaccination at younger ages might be reduced either because of the infant having a less mature immune system or because of high maternal antibodies, which might interfere with live vaccine virus replication [10]. HRV was not evaluated in a clinical efficacy trial in a developing population in Asia prior to the 2009 WHO recommendation of global rotavirus vaccination [11]. Finally, while current rotavirus vaccines have been found in individual-randomized trials conducted in populations with high child mortality to be about 40% to 60% efficacious (i.e., direct protection) against severe rotavirus gastroenteritis [4–6,12], large-scale programmatic introduction of rotavirus vaccine might reduce overall transmission of rotavirus and provide substantial indirect protection to those who remain unvaccinated. This indirect protection would also improve the total protection for vaccinated infants, i.e., the combined protection conferred by the direct and indirect effects experienced by vaccinated children living in a vaccinated population [13]. Evidence of possible indirect protection was provided after introduction of rotavirus vaccines in industrialized countries, where a rapid decline in the rate of acute rotavirus diarrhea (ARD) among infants and children too young or too old to have received the vaccines was observed [14–18]. The ability of a vaccination program to provide such indirect and additional protection is of particular interest for use of rotavirus vaccines in low- and middle-income countries, as this might reduce the gap in the levels of protection afforded to infants in such countries compared to high-income countries.

To address these outstanding questions, PATH’s Rotavirus Vaccine Program and the International Centre for Diarrhoeal Disease Research, Bangladesh (icddr,b) partnered to design and conduct an effectiveness study of HRV in Bangladesh. The primary objective of the trial was to estimate the “overall effectiveness” [13] of an HRV vaccination program in reducing the risk of presenting with ARD to a treatment facility among all children who had been age-eligible for vaccination with HRV during the vaccination program. Overall effectiveness is a combination of (a) the direct and indirect effects experienced by vaccinated children living in a vaccinated population and (b) the indirect effects experienced by unvaccinated children living in that same population.

## Methods

The Western Institutional Review Board (Puyallup, Washington, US) approved this study (WIRB protocol number 2008–0624). The ethical review committee of icddr,b in Bangladesh also approved this study (protocol 2007–024). The study was conducted in accordance with the principles of the Declaration of Helsinki and in compliance with International Conference on Harmonisation Good Clinical Practice guidelines.

### Study design

The study was an open-label, cluster-randomized (by village), parallel-group field trial with an observed-only control group. The trial was conducted in rural Bangladesh among the infant population of icddr,b’s Matlab Health and Demographic Surveillance System (Matlab HDSS) [19]. The Matlab HDSS has a child population under 2 y of age of approximately 10,000. It comprises 142 villages geographically divided into two administrative areas designated as the icddr,b service area (ISA) (67 villages) and the government service area (GSA) (75 villages). In the ISA, icddr,b provides an extensive intervention program of maternal, child health, and family planning services, including routine immunization; in the GSA, the Bangladesh Ministry of Health and Family Welfare provides its standard public health and immunization services. In this trial, all Matlab HDSS villages were selected as clusters, and an HRV vaccination program was introduced at the village level in which HRV was scheduled to be given along with other standard infant vaccines at 6 and 10 wk of age in villages allocated to receive HRV.

As of March 2017, the government of Bangladesh had not yet included rotavirus vaccination in the routine immunization program, although it had hoped to introduce it in 2014 [20]. Before the trial, HRV was neither available in nor affordable to the Matlab HDSS population. Thus, written informed consent at enrollment (vaccination) was obtained for infants receiving HRV. But for infants in control clusters or infants in HRV clusters who did not receive HRV, written informed consent for administration of only standard Expanded Programme on Immunization (EPI) vaccines without HRV was not sought since these vaccines were the public health standard in Bangladesh. A cluster-level assent was also not utilized. This plan was accepted by national and local government health authorities and by overseeing ethics committees. After the study was completed, the investigators offered vaccination with HRV free of cost to all age-eligible infants in the entire Matlab HDSS for a period of 3 y to allow time for discussions between the Bangladesh Ministry of Health and Family Welfare and Gavi, the Vaccine Alliance, to support the introduction of rotavirus vaccination into the national immunization program.

### Participants

All infants in villages randomized for introduction of HRV were invited to participate if they met eligibility criteria for rotavirus vaccination at the time of presentation at EPI centers for routine immunizations. Inclusion criteria for receipt of HRV were being 6 to 20 wk of age, having primary residence at the time of DTP1 receipt in a village selected for introduction of HRV, and having a parent or guardian provide written informed consent; exclusion criteria were history of intussusception, hypersensitivity to the active substance or any component in the vaccine, uncorrected congenital malformation of the gastrointestinal tract, or known or suspected immunodeficiency. Infants with an acute febrile illness were temporarily excluded from HRV vaccination only if that illness was severe enough to warrant postponement of other EPI vaccinations. Infants with current diarrhea and/or vomiting were not excluded unless the illness met the aforementioned temporary exclusion criterion.

Because the design of the trial required monitoring the entire population younger than 2 y for ARD, regardless of receipt of HRV, a separate written informed consent was utilized for collection of clinical data and stool samples for all such children presenting to a treatment facility with diarrhea. No specific education nor instruction on presenting for diarrhea was given to parents except for what was described on the information sheet accompanying the informed consent form. However, because of the long-standing relationship between the residents of the Matlab HDSS villages and the icddr,b, patients with severe diarrhea in the area generally seek care at the icddr,b facilities.

### Randomization and masking

In this trial, existing administrative units of the Matlab HDSS, called “villages,” were chosen as the unit of randomization. Although the primary residential unit in villages is the bari, a group of households grouped together around a common yard, the larger village unit was considered more feasible and was hypothesized to reduce contamination, defined for a cluster-randomized trial as the possibility that outcomes in individuals in one cluster would be distorted because of contacts with individuals from outside of the cluster [21]. Additionally, villages correspond more closely to the EPI centers providing routine immunization services.

Villages of the GSA and ISA were randomized separately in a 1:1 ratio for introduction of HRV or not. Prior to study initiation, PATH computer-generated the allocation sequences for the GSA and ISA using block randomization with block sizes of 12. To ensure that the groups were similar in terms of diarrhea rates prior to the intervention, population and diarrheal admission data for children under 2 y of age for 2005 and 2006 were utilized to restrict valid randomizations to those with a 2005–2006 average annual diarrheal admission rate difference between study arms of no greater than 0.5%. The generated allocation sequences were then securely transferred to the principal investigator, who distributed the sequences to the field supervisors who oversaw HRV vaccinations.

The study was conducted open-label without masking, and field staff conducting the vaccinations were unblinded. However, medical staff collecting clinical data on diarrheal presentations and laboratory personnel conducting assays on stools were not informed of previous HRV receipt of participants.

### Procedures

In villages randomized for introduction of HRV, one 1-ml dose of HRV (Rotarix; GSK Biologicals, Rixensart, Belgium) was delivered orally to participating infants—along with other routine vaccines, including OPV—at the DTP1 and DTP2 immunization visits, recommended in Bangladesh to occur at 6 and 10 wk of age. In the GSA, icddr,b study staff conducted the informed consent process and collected study-specific baseline data prior to vaccination, but regular government public health staff administered all vaccines, including HRV. In the ISA, icddr,b study staff conducted the informed consent process, collected study-specific baseline data, and administered all vaccines, including HRV. For participants who might have already received DTP1 elsewhere, HRV doses were allowed to be given with DTP2 and DTP3. After vaccination with dose 2 of HRV, no study follow-up visits were scheduled to be conducted for any child in the Matlab HDSS. Only demographic surveys of the population were made bimonthly as part of routine administration of the Matlab HDSS. Serious adverse events among infants vaccinated with HRV were assessed by the principal investigator or trained study physicians and followed to resolution.

**Fig 1:**
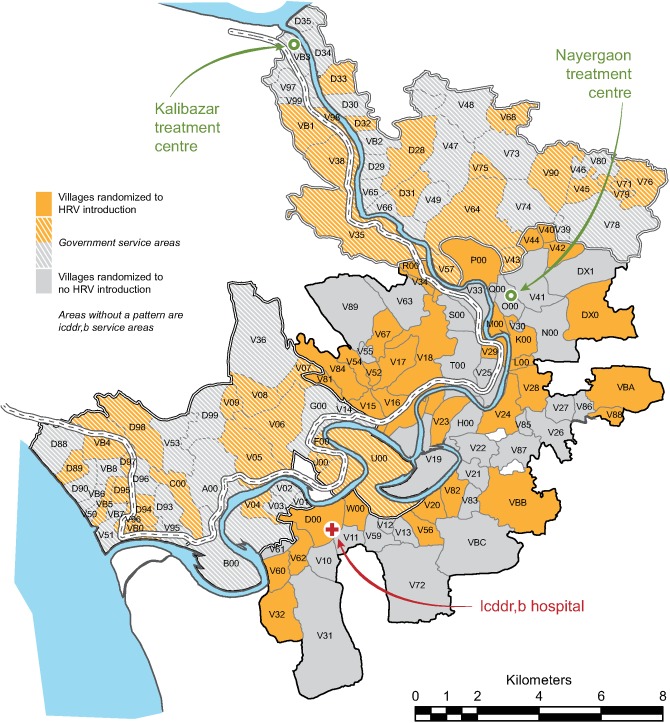
Distribution of villages randomized to human rotavirus vaccine introduction or no human rotavirus vaccine introduction during the trial, Matlab Health and Demographic Surveillance System. HRV, human rotavirus vaccine; icddr,b, International Centre for Diarrhoeal Disease Research, Bangladesh.

Within the Matlab HDSS, icddr,b administers Matlab Diarrhoea Hospital and two community treatment centers (locations shown in Fig 1). Surveillance for diarrhea among all Matlab HDSS children under 2 y of age was conducted at these facilities to identify study outcomes. Children less than 2 y of age presenting to any of these facilities with diarrhea were assessed, including collecting data to calculate the Vesikari score [22], and treated according to their degree of dehydration, and stool specimens were collected.

Vaccination of eligible participants with HRV in villages randomized for introduction of HRV in both the GSA and ISA and surveillance for diarrheal outcomes at Matlab Diarrhoea Hospital and the two community treatment centers continued concurrently and continuously from study initiation until the date of study completion, March 31, 2011.

### Outcomes

The primary study outcome was ARD among children less than 2 y of age who were brought to Matlab Diarrhoea Hospital or one of the two community treatment centers. Diarrhea was defined as three or more looser-than-normal stools in a 24-h period. Confirmation of ARD was by detection of rotavirus antigen (group A rotavirus-specific VP6 proteins) in stool using a qualitative enzyme immunoassay (ProSpecT Rotavirus Microplate Assay, Oxoid, Basingstoke, UK). Stool was not specifically tested for vaccine-type virus.

Secondary study outcomes were G- and P-type-specific ARD, severe ARD (Vesikari score ≥ 11), and acute diarrhea associated with enterotoxigenic *Escherichia coli* (ETEC). A detailed clinical assessment was made for each child using previously developed methods [6]. A modified Vesikari severity score [22] was calculated for each diarrheal episode by a blinded PATH scientist. Rotavirus G and P types were also determined by multiplex reverse transcription polymerase chain reaction and confirmed by Sanger sequencing of VP7 and VP4 gene segments [23]. In addition, a supplementary laboratory assay was conducted on stools to test for the presence of ETEC [24].

### Statistical analysis

The sample size was calculated using methods that condition on the total number of outcomes observed [25,26]. For the primary objective, we assumed an overall effectiveness of 50%. Had this been an individual-randomized trial, 77 ARD outcomes among all age-eligible infants from HRV and non-HRV villages would have been required to ensure that the study had a minimum power of 80% to rule out a lower bound of the two-sided 95% CI of zero. However, for a cluster-randomized trial, the sample size must be inflated by a “design effect” factor to account for the correlation of participant outcomes within village clusters. Based on available data, clusters contained an average of 65 children younger than 2 y, with a coefficient of variation in the size of this age group by village of 0.96. Factoring in these values, along with an assumption of an intracluster correlation coefficient of 0.02, we calculated a design effect of 3.48 using published methods [27]. This inflated the minimum number of ARD outcomes needed to 268. Assuming a 3.5% cumulative incidence of ARD in non-HRV villages during the entire study period, a total sample size of 10,210 infants (5,105 in each group) was then estimated.

After completion of the study, three separate databases were linked for analyses: (a) the Matlab HDSS population database containing demographic data for children under 2 y of age at any time during the study period and their routine immunization data, (b) the database containing demographic data and data on receipt of HRV and routine immunizations for participants consenting to receive HRV, and (c) the surveillance database containing clinical and laboratory data for children presenting with diarrhea to Matlab Diarrhoea Hospital or one of the two community treatment centers.

For the analysis of overall vaccine effectiveness in the age-eligible residents of the Matlab HDSS (primary objective), an intention-to-treat-like approach that disregarded actual receipt of HRV and counted all ARD outcomes occurring from 6 wk of age was appropriate. Thus, the incidence rate of ARD among all children age-eligible to have received HRV, regardless of whether they did, in villages where HRV was introduced was compared to that among all children of equivalent age eligibility in villages where HRV was not introduced. Adjusted vaccine effectiveness (and its associated 95% CI) was calculated as one minus the incidence rate ratio times 100%. This incidence rate ratio was estimated as the exponentiated coefficient for the main effect (dummy variable for village HRV allocation) from a Poisson regression model of the village-level counts of ARD among the age-eligible members of the Matlab HDSS, with an offset of the person-years of exposure at the village level and a dummy variable for the ISA (to account for stratified randomization). A Pearson chi-squared scale parameter was included to allow for overdispersion in the distribution of the village-level ARD counts and to account for within-village correlation (clustering) [28]. The adjusted rate difference between non-HRV and HRV villages (and its associated 95% CI) was estimated, as described in Section 12.3.2 of [21], using the optimal weights approach to estimate the variance of the village-level incidence rate within strata defined by combinations of service area and treatment arm. This approach adjusted the rate difference estimates to account for the stratified randomization. All remaining vaccine effectiveness parameters and rate differences were estimated using the same approach as for the overall effectiveness—modified intention-to-treat (mITT)—with the exception that the portion of the study population contributing to the estimation of each parameter differed, as described in the following paragraphs. Analyses were implemented using Stata, version 13 (StataCorp).

An artifact of rotavirus vaccine introduction is that many age-eligible children (up to 20 wk of age) in HRV villages may not have been vaccinated with HRV because they had already received scheduled vaccinations before study initiation. To avoid diluting the overall effectiveness estimate, the primary analysis included only resident infants who turned 6 wk of age during the period of the trial (i.e., after study initiation). Individual-level person-time of exposure was counted from 6 wk of age until 2 y of age, the end of the study, or the date of first presentation to a treatment facility with an outcome of ARD, whichever came first. Nonetheless, a supportive analysis of overall vaccine effectiveness that included all age-eligible children (up to 20 wk of age at study initiation) was conducted. In both analyses of overall effectiveness, in-migrants into the Matlab HDSS population were included in the analyses if they immigrated during the study period while they were between 6 and 20 wk of age. For these analyses, person-time of exposure was summed at the village level.

For calculations of total effectiveness among vaccinees, both a mITT approach and an according-to-protocol (ATP) approach were used. Because this was an open-label trial with an observed-only control group and the hypothetical date of vaccination with a placebo cannot be known, for both total effectiveness analyses person-time for all participants was counted from the date of receipt of the first dose of OPV. For the mITT analysis, infants in HRV villages must have received at least one dose of HRV and OPV (exclusive of birth dose), and infants in non-HRV villages must have received at least one dose of OPV by their third post-birth EPI visit, i.e., DTP3. Then, for all children, regardless of study arm, person-time was counted starting from the date of receipt of the first of OPV1 or OPV2 received while resident in a study village. For the ATP analysis, infants in HRV villages must have received two doses of HRV and OPV (exclusive of birth dose), and infants in non-HRV villages must have received two doses of OPV (also exclusive of birth dose). Then, person-time was counted starting from 2 wk after the date of receipt of OPV2, again regardless of study arm. In-migrants were included in the analyses if they immigrated during the study when they were between 6 and 20 wk of age.

For all analyses, only first ARD outcomes were counted. Effectiveness estimates were stratified post hoc by first and second year of life.

## Results

**Fig 2:**
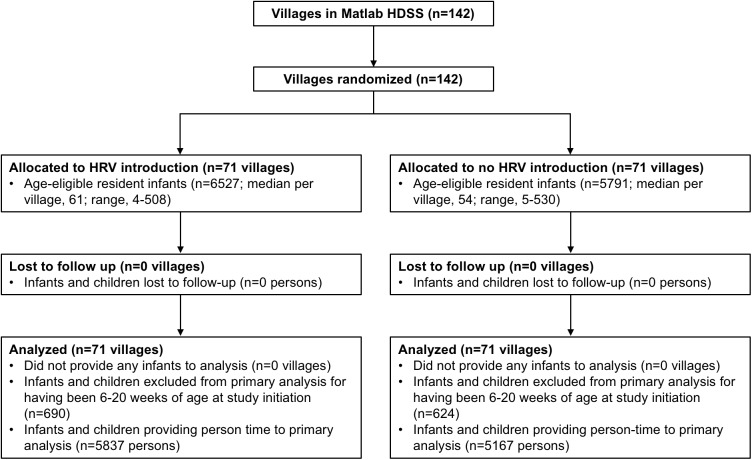
Study profile for the primary objective of overall effectiveness. HRV, human rotavirus vaccine; Matlab HDSS, Matlab Health and Demographic Surveillance System.

The study was initiated on November 1, 2008, in the GSA and on April 1, 2009, in the ISA. The delayed initiation in the ISA was to allow for the completion of a separate research protocol [6]. All 142 villages of the Matlab HDSS were randomized, and during the period of the trial (until March 31, 2011), 12,318 infants in these villages were Matlab HDSS residents age-eligible for vaccination with HRV. There were 71 villages randomized to HRV introduction (6,527 age-eligible infants) and 71 villages randomized to no HRV introduction (5,791 age-eligible infants) (Fig 2). Fig 1 shows the geographic distribution of Matlab HDSS villages randomized to introduction of HRV or not. Person-time was contributed to the primary analysis of overall effectiveness by 11,004 infants (89.3%) (Fig 2).

Table 1 shows the baseline characteristics of randomized villages and the children living in those villages. While HRV villages were modestly larger on average, cluster-level characteristics were generally similar between the study arms. In HRV villages, 4,808 (73.7%) infants received the first dose of HRV, at an average age of 8.6 wk, and 4,595 (70.4%) of these received the second dose, at an average age of 13.1 wk. Receipt of OPV was somewhat differential by study arm, with HRV villages having a dose 1 coverage of 96.1% and non-HRV villages having a coverage of 91.6%.

**Table 1:** Baseline demographic and clinical characteristics of each study group.

Category	Characteristic	HRV arm	Non-HRV arm
**Cluster-level characteristics**	Number of villages	71	71
	Total population (all ages)^a^	116,649	105,569
	Average age of population (years)^a^	27.9	28.0
	Mean (s.d.) village population size^a^	1,643 (1,557)	1,487 (1,531)
	Total population <2 y of age^a^	5,258	4,881
	Number of live births per annum^a^	2,666	2,414
	Mean (s.d.) birth rate per 1,000^a^	21.8 (4.8)	22.6 (7.0)
	Mean number of children <2 y of age per village^a^	74	69
	Mean number of baris per village^a^	38	36
	Mean number of children <2 y of age per bari^b^	2	2
	Mean (s.d.) distance of the centroid of each village to Matlab Diarrhoea Hospital (meters)	7,455 (3,802)	7,534 (4,140)
	Mean annual presentation rate to Matlab Diarrhoea Hospital for diarrhea per 1,000 children <2 y of age (2000–2006)	63.7	66.6
**Individual level variables**^**c**^	**Number of children**	6,527	5,791
	**Percent male**	50.4%	51.1%
	**OPV vaccination**		
	Number (percent) received dose 1	6,271 (96.1%)	5,302 (91.6%)
	Mean (s.d.) age at dose 1 (weeks)	8.6 (2.2)	8.9 (2.8)
	Number (percent) received dose 2	6,082 (93.2%)	5,113 (88.3%)
	Mean (s.d.) age at dose 2 (weeks)	13.3 (2.7)	13.8 (3.3)
	**HRV vaccination**		
	Number (percent) received dose 1	4,808 (73.7%)	—
	Mean (s.d.) age at dose 1 (weeks)	8.6 (1.9)	—
	Number (percent) received dose 2	4,595 (70.4%)	—
	Mean (s.d.) age at dose 2 (weeks)	13.1 (2.2)	—
	**Number who contributed person-time to primary analysis of overall VE**^**d**^	5,837 (89.4%)	5,167 (89.2%)

^a^In 2008 (at the time of randomization); data from 2008 Matlab HDSS.

^b^Not a randomization unit parameter.

^c^Children who were Matlab HDSS residents and were age-eligible for receipt of HRV (i.e., were residents during the period of the trial while they were between 6 and 20 wk of age).

^d^Children who were Matlab HDSS residents during the period of the trial on the date they were 6 wk of age.

HRV, human rotavirus vaccine; Matlab HDSS, Matlab Health and Demographic Surveillance System; OPV, oral poliovirus vaccine; s.d., standard deviation; VE, vaccine effectiveness.

In the primary analysis of overall vaccine effectiveness, 164 outcomes of ARD occurred among all children from HRV villages (incidence rate of 2.80 cases per 100 person-years) and 206 occurred among all children from the non-HRV villages (incidence rate of 4.10 cases per 100 person-years), yielding an overall effectiveness of 29.0% (95% CI, 11.3% to 43.1%) (Table 2). This translated into a reduction of 1.28 presentations for ARD per 100 person-years among age-eligible infants in villages where HRV was introduced at a coverage rate of approximately 74% (as noted in Table 1). The coefficient of variation, *k*, for the event rate data contributing to the primary analysis was estimated using the non-HRV villages in the GSA: 0.22 (it was undefined for the ISA). For presentations of severe ARD, overall effectiveness was slightly lower and did not reach statistical significance (22.9% [95% CI, −0.2% to 40.7%]) (Table 2). Including infants who were up to 20 wk of age on the day of study initiation in the respective areas of the Matlab HDSS resulted in slightly lower estimates of overall effectiveness for all presentations of ARD (24.9% [95% CI, 7.7% to 38.9%]) and for presentations of severe ARD (20.4% [95% CI, −1.8% to 37.7%]), which did not reach statistical significance (Table 2). In an exploratory analysis of overall effectiveness against first presentation for diarrhea due to any etiology, overall effectiveness was −2.2% (95% CI, −14.4% to 8.7%).

**Table 2:** Overall effectiveness of the human rotavirus vaccination program in preventing presentations of acute rotavirus diarrhea of any severity and severe acute rotavirus diarrhea among age-eligible children less than 2 y of age, regardless of actual receipt of human rotavirus vaccine.

ARD analysis	HRV villages			Non-HRV villages			Adjusted VE_O_^b^, percent (95% CI)	Adjusted rate difference^c^, percent (95% CI)
	Cases (*n*)	Person-years	Incidence rate^a^	Cases (*n*)	Person-years	Incidence rate^a^		
**Including resident infants who turned 6 wk of age on or after study initiation**								
Any severity^d^	164	5,857	2.80	206	5,026	4.10	29.0 (11.3, 43.1)	1.28 (0.31, 2.25)
Severe ARD^e^	128	5,880	2.18	149	5,058	2.95	22.9 (−0.2, 40.7)	0.83 (−0.04, 1.71)
**Including above infants plus those up to 20 wk of age at study initiation**								
Any severity	195	6,960	2.80	235	6,031	3.90	24.9 (7.7, 38.9)	1.12 (0.24, 2.01)
Severe ARD^e^	151	6,992	2.16	172	6,068	2.83	20.4 (−1.8, 37.7)	0.74 (−0.07, 1.54)

^a^Per 100 person-years.

^b^Estimated using a Poisson regression model with a Pearson chi-squared scale parameter to account for clustering.

^c^Estimated per 100 person-years using the approach described in Section 12.3.2 of [21].

^d^Primary analysis.

^e^Person-time censored at first severe ARD episode, regardless of severity of previous ARD.

ARD, acute rotavirus diarrhea; HRV, human rotavirus vaccine; VE_O_, overall vaccine effectiveness.

In the mITT analysis of total effectiveness among vaccinees against ARD, 108 outcomes occurred among children receiving HRV in HRV villages (incidence rate of 2.28 cases per 100 person-years) and 194 occurred among children receiving only OPV in non-HRV villages (incidence rate of 3.88 cases per 100 person-years), for a total effectiveness of 38.7% (95% CI, 20.6% to 52.7%) (Table 3). This translated into a reduction of 1.39 presentations for ARD per 100 person-years among vaccinated children. The ATP analysis of total effectiveness against ARD was very similar, with 102 outcomes occurring among children receiving HRV in HRV villages (incidence rate of 2.48 cases per 100 person-years) and 172 occurring among children receiving only OPV in non-HRV villages (incidence rate of 4.42 cases per 100 person-years), for a total effectiveness of 41.4% (95% CI, 23.2% to 55.2%) (Table 3). This translated into a reduction of 1.73 presentations for ARD per 100 person-years among vaccinated children. Total effectiveness estimates were modestly higher for prevention of ARD presentation during the first year of life as compared to the second year of life (45.2% [95% CI, 26.3% to 59.3%] versus 28.9% [95% CI, −15.6% to 56.3%]) (Table 3). However, confidence intervals widely overlapped, and second-year estimates did not reach statistical significance. Of the 274 presentations of ARD included in the ATP analysis of total effectiveness, 222 (81.0%) were episodes in which a single strain of rotavirus was identified for which both P and G type could be determined. Five strains of rotavirus—G1P[8] (25.2%), G2P[4] (12.2%), G9P[8] (15.3%), G12P[6] (11.7%), and G12P[8] (34.2%)—accounted for 98.6% of these cases. Total effectiveness estimates by strain were generally similar (Table 3). Total effectiveness estimates for G9P[8] and G12P[6] were both lower, but again confidence intervals were wide and estimates did not reach statistical significance. When categorized as fully or partially homotypic versus fully heterotypic with respect to the G and P type of the vaccine, total effectiveness estimates were again similar, 48% and 43%, respectively (Table 3).

**Table 3:** Total effectiveness of human rotavirus vaccine in preventing presentations of acute rotavirus diarrhea of any severity and severe acute rotavirus diarrhea among vaccinees, by age of onset and rotavirus strain detected.

ARD analysis	HRV villages			Non-HRV villages			Adjusted VE_T_^c^, percent (95% CI)	Adjusted rate difference^d^, percent (95% CI)
	Cases (*n*)	Person-years^a^	Incidence rate^b^	Cases (*n)*	Person-years^a^	Incidence rate^b^		
**VE**_**T**_ **(mITT)**								
Any severity, all ages	108	4,735	2.28	194	4,998	3.88	38.7 (20.6, 52.7)	1.39 (0.47, 2.32)
**VE**_**T**_ **(ATP)**								
Any severity, all ages	102	4,117	2.48	172	3,893	4.42	41.4 (23.2, 55.2)	1.73 (0.64, 2.81)
Onset <12 mo	75	2,464	3.04	135	2,340	5.77	45.2 (26.3, 59.3)	2.61 (1.10, 4.12)
Onset 12–23 mo	27	1,652	1.63	37	1,553	2.38	28.9 (−15.6, 56.3)	0.36 (−0.89, 1.62)
G1P[8]	18	4,166	0.43	38	3,984	0.95	54.5 (18.7, 74.6)	—
G2P[4]	10	4,166	0.24	17	3,988	0.43	45.8 (−42.4, 79.4)	—
G9P[8]	15	4,166	0.36	19	3,991	0.48	20.0 (−44.7, 55.8)	—
G12P[6]	10	4,167	0.24	16	3,987	0.40	36.4 (−39.3, 71.1)	—
G12P[8]	24	4,164	0.58	52	3,978	1.31	51.5 (18.5, 71.1)	—
Homotypic strain, all ages^e^	57	4,147	1.37	111	3,950	2.81	48.1 (27.9, 62.7)	—
Heterotypic strain, all ages^e^	20	4,159	0.48	34	3,973	0.86	43.0 (−2.9, 68.4)	—
Severe^f^, all ages	75	4,135	1.81	130	3,919	3.32	42.8 (22.1, 57.9)	1.38 (0.42, 2.35)
Onset <12 mo	53	2,472	2.14	101	2,351	4.30	48.0 (27.0, 63.0)	2.08 (0.75, 3.41)
Onset 12–23 mo	22	1,664	1.32	29	1,568	1.85	25.8 (−29.5, 57.5)	0.36 (−0.80, 1.52)

^a^Person-time censored at first ARD episode.

^b^Per 100 person-years.

^c^Estimated using a Poisson regression model with a Pearson chi-squared scale parameter to account for clustering.

^d^Estimated per 100 person-years using the approach described in Section 12.3.2 of [21].

^e^Person-time censored at first severe ARD episode, regardless of severity of previous ARD.

^f^Homotypic includes fully (G1P1A[8]) or partially (G1P# or G#P1A[8]) homotypic strains, while heterotypic includes strains that are neither G1 nor P1A [8]. # indicates any G or P serotype/genotype.

ARD, acute rotavirus diarrhea; ATP, according-to-protocol; HRV, human rotavirus vaccine; mITT, modified intention-to-treat; VE_T_, total vaccine effectiveness.

In the ATP analysis of total effectiveness against severe ARD presentation, 75 outcomes of severe ARD occurred among those receiving HRV in HRV villages (incidence rate of 1.81 cases per 100 person-years) and 130 occurred among those receiving only OPV in non-HRV villages (incidence rate of 3.32 cases per 100 person-years), for a total effectiveness of 42.8% (95% CI, 22.1% to 57.9%) (Table 3). This translated into a reduction of 1.38 presentations for severe ARD per 100 person-years among vaccinated children. Again, total effectiveness estimates were modestly higher for prevention of severe ARD presentations during the first year of life as compared to the second year of life (48.0% [95% CI, 27.0% to 63.0%] versus 25.8% [95% CI, −29.5% to 57.5%]) (Table 3).

Indirect effectiveness against ARD among children resident in HRV villages who were 20 wk to 2 y of age at study initiation was statistically nonsignificant, at −1.2% (95% CI, −43.9% to 28.8%), and indirect effectiveness against ARD among age-eligible infants who did not receive HRV but did receive at least one dose of OPV was statistically nonsignificant, at 9.2% (95% CI, −40.8% to 41.5%).

To explore the possibility that effectiveness estimates against ARD might be biased in this cluster-randomized trial with an observed-only control group, total effectiveness of HRV against acute diarrhea testing positive for ETEC was estimated. Total effectiveness against acute ETEC diarrhea was close to zero, excluding rotavirus co-positive cases (5.3% [95% CI, −55.4% to 42.3%]) or including them (4.0% [95% CI, −46.5% to 37.1%]).

During the course of the study, 13 serious adverse events were identified among vaccinees, and all were deemed to be unrelated to receipt of HRV (Table 4).

**Table 4:** Serious adverse events identified among human rotavirus vaccine recipients.

Serious adverse event	Outcome	Relatedness to HRV receipt
Meningitis with hypoglycemia	Fatal	Not related
Accidental drowning	Fatal	Not related
Pneumonia with malnutrition	Fatal	Not related
Pneumonia with congenital cyanotic heart disease	Fatal	Not related
Accidental kerosene oil poisoning	Fatal	Not related
Pneumonia	Fatal	Not related
Biliary atresia	Fatal	Not related
Accidental drowning	Fatal	Not related
Accidental drowning	Fatal	Not related
Accidental suffocation	Fatal	Not related
Febrile convulsions with malnutrition	Fatal	Not related
Febrile convulsions with sepsis	Fatal	Not related
Suspected ileocolic intussusception^a^	Recovered	Not related

^a^A 4-mo-old female was admitted to Matlab Diarrhoea Hospital with bloody stool, frequent vomiting, and a round mass in the hypogastric region suggestive of ileocolic intussusception. The infant was transferred to Dhaka Shishu Hospital, where the child was managed with laparotomy with manual release under general anesthesia. She was discharged 6 d later after full recovery. She had received dose 1 of HRV at 6.3 wk of age and dose 2 at 15.3 wk of age. Onset of this event was 23 d after dose 2 of HRV. The investigator considered the event unrelated to study vaccination but still documented the event carefully and reported it to regulatory authorities and the manufacturer as required by the protocol.

HRV, human rotavirus vaccine.

## Discussion

This study was able to document the benefit of incorporating HRV into a routine EPI program, and demonstrated that HRV provided a moderate level of impact. During the nearly two and a half years of this study in rural Bangladesh, HRV prevented more than a quarter of all acute rotavirus diarrheal presentations occurring among infants who were age-eligible to have received the vaccine. Because overall effectiveness calculations include cases among unvaccinated infants in villages where vaccine is introduced, overall effectiveness is dependent on vaccine coverage levels. Total effectiveness is a more generalizable estimate (to vaccine recipients), and in the absence of measureable indirect effects and self-selection bias, total effectiveness reduces to direct effectiveness, the parameter closely matching efficacy calculated in individual-randomized trials. As we found no evidence for substantial indirect effects of vaccination during the study period, the total effectiveness estimate is likely a reasonable estimate of the direct protection of HRV given with DTP1 and DTP2 in low-resource populations such as this one. Total effectiveness of HRV in preventing ARD presentation was 41% (ATP). This translates into approximately two cases prevented for every 100 person-years among vaccinees. Direct effectiveness was not directly estimated as it is likely to be a biased measure in a study where within-cluster receipt of HRV was not randomized.

This trial is to our knowledge the first effectiveness study of a rotavirus vaccination program in which rotavirus diarrhea rates were compared between large clusters receiving and not receiving HRV over an extended period in a Gavi-eligible country in Asia, and the first study attempting to study HRV overall effectiveness, a combination of direct and indirect vaccine effects in vaccinated and unvaccinated children. A major strength of this study is that it used an experimental design to prospectively measure the effectiveness of rotavirus vaccination under routine program conditions where inclusion/exclusion criteria were minimal.

Total effectiveness of HRV was moderate in this community with excellent knowledge about early oral rehydration therapy at home and high access to medical care for diarrhea. Even though impact might be even greater in areas with less medical care access and oral rehydration therapy use, our rate reduction estimates still likely well underestimate the true public health value of rotavirus vaccination in this population because many cases probably never present to Matlab Diarrhoea Hospital or one of the two community treatment centers. For example, annual gastroenteritis presentation rates for children under 2 y of age to Matlab Diarrhoea Hospital from the GSA have historically been half of those from the ISA because of additional distance to travel for many in the GSA. While our primary outcome was presentation to a treatment facility for ARD regardless of severity, most (three-fourths) of the cases presenting to the Matlab Diarrhoea Hospital met criteria to be scored as severe by a modified Vesikari scoring system. Total effectiveness against severe ARD was nearly identical to that against ARD presentation of any severity. This estimate is very similar to the efficacy estimate generated in an individual-randomized trial of the pentavalent rotavirus vaccine (RotaTeq) in this same population using nearly identical methods of case identification (42.7% [95% CI, 10.4% to 63.9%] against severe ARD) [6]. It is also comparable to the efficacy (53.6% [95% CI, 35.0% to 66.9%]) of another HRV (Rotavac), given as three doses, in the only other Gavi-eligible country in Asia in which rotavirus vaccine efficacy data have been generated, India [12].

We found no evidence that total effectiveness estimates differed importantly by strain, although there was a lack of precision around strain-specific total effectiveness estimates and the study was not designed to identify strain-specific differences in effectiveness. Studies have not identified reduced effectiveness of rotavirus vaccines against strains not contained in the vaccines [29]. The predominant genotypes circulating in Matlab during the time of this efficacy trial varied, as documented recently [30]. Immediately prior to the study initiation, G1P[8] was the predominant genotype, but then G12P[8] predominated. Whether the vaccination program exerted any pressure toward genotype replacement is not known. In Bangladesh, genotypes have been quite variable over time [31]; this change seen during the duration of the study was interesting, but further studies are needed to determine if rotavirus vaccination can exert sufficient selection pressure to cause strain replacement.

This study was designed as a cluster-randomized trial primarily to take advantage of the programmatic systems for delivery and administration of childhood vaccines in this population. This design also allowed for the potential inducement and measurement of indirect effects, but none were found over the study period of two and a half years. There are several possible explanations for this. First, unlike other cluster-randomized vaccine trials where large numbers of the population can be vaccinated quickly to reach high coverage [32,33], the age requirements for HRV receipt result in coverage slowly increasing over time as infants are born and reach the age for receipt of HRV. Data for this population show that children are at risk for rotavirus transmission through at least 23 mo of age [31]; thus, high coverage of vaccination among children at risk for rotavirus (at risk through approximately 2 y of age) in villages where HRV was introduced was not even possible until nearly 2 y after the study began. It may be that more observation time is needed to measure the impact of the program, which matured only near the end of the trial. Second, although the Matlab HDSS, with a 2010 population of 225,000, is quite large, it is located in central Bangladesh (population 151.1 million) and bisected by numerous rivers and floodplains. With high population densities, Matlab villages are geographically small and therefore susceptible to “contamination” in a cluster-randomized trial. Rotavirus transmission might have been introduced into vaccinated villages from neighboring unvaccinated villages or from outside of the Matlab HDSS area, thus negating any reduction in rotavirus transmission that vaccination may have caused. National rotavirus vaccination programs in low-resource settings might provide indirect effects even where incidence is high and protection by vaccine only modest, but substantial effects likely require coverage across a much larger population than our study was designed to reach.

A potential limitation of this study is that it was an open-label study with an observed-only control group (no placebo). Because the overall effectiveness estimate included all age-eligible children in randomized villages, that estimate should be unbiased under randomization. On the other hand, total effectiveness, which compares subsets of children (those self-selecting for participation) in randomized villages, could have been biased. For example, if parents who elected to receive rotavirus vaccine and other EPI vaccinations for their child were more likely to present to Matlab Diarrhoea Hospital or one of two community treatment centers when their child developed diarrhea compared to parents who did not elect to participate, total effectiveness could be underestimated. We had no control vaccine or placebo in non-HRV villages and therefore had no way to control for self-selection of participation in those clusters. To reduce this type of bias, we attempted to remove from the analysis those in control villages who would not have participated if we had had a placebo by basing total effectiveness estimates on analyses requiring acceptance of OPV vaccination. Further selection bias could have been introduced because we relied on parent or guardian decision to present for health care when their child had an episode of diarrhea. However, we do not believe that the addition of one vaccine to the EPI schedule was likely to change health-seeking behaviors in this population with common knowledge of quality care at Matlab Diarrhoea Hospital and the community treatment centers. Additionally, clinical and laboratory staff remained blinded during the study. The bias indicator (effectiveness against ETEC diarrhea) did not demonstrate a bias.

Total effectiveness estimates in the second year of life were substantially lower than in the first year of life. In this study, enrollment, vaccination, and surveillance occurred concurrently and continuously until the last day of the study, and so over half of infants in the study population were not followed through their second year of life, when rotavirus disease is still common. Thus, estimates for the second year of life are much less precise than if all enrolled infants had been followed to their second birthday, and we cannot draw firm conclusions. Two reasons are hypothesized for measurement of a decline in protection with age. Rates of ARD typically decrease with age, suggesting the development of immunity from natural exposure, and in this study, the rate among children in non-HRV villages during their second year of life was less than half of that observed in their first year of life. It may be that the interaction with natural exposure, which occurred in both the vaccinated and unvaccinated groups, narrowed the difference in immunity between the two groups over time, leading to higher measured relative risk (or lower measured vaccine effectiveness). It is not clear how much ongoing rotavirus circulation can account for this effect, which may be different in different epidemiological settings, especially given that high rates of moderate-to-severe rotavirus diarrhea are measured in many populations well into the second year of life. Alternatively, the reduced effectiveness may reflect actual waning of immunity from the vaccine.

Our results are similar to results from a trial in South Africa and Malawi in which significant efficacy of two-dose HRV was not found during the second year of life [5]. However, that study was not originally designed to estimate efficacy in the second year of life. Case–control observational effectiveness studies of rotavirus vaccine in routine use in Latin American, Europe, the Middle East, and Africa demonstrate that effectiveness may be preserved through 2 y of age in many under-resourced populations [34–45]. While results depend on the vaccine used, the outcome measured, and the location, they nonetheless suggest that effectiveness in the second year of life is frequently lower than during infancy. It seems evident that protection during the first year of life would be particularly important because infants are especially vulnerable. Data from multiple countries nonetheless indicate that rotavirus is still a primary cause of medically important disease (moderate-to-severe diarrhea) well into the second year of life [1]. As rotavirus vaccination programs are rolled out in low-resource populations, they may reduce the force of infection and shift the average age of severe rotavirus diarrhea later. In the high-income setting of the US, rotavirus vaccination is highly effective, and this effectiveness appears to persist at high levels, such that severe rotavirus infections are not shifted to older children [46]. Until data are more widely available for low- and middle-income countries, maintaining protection past infancy and into early childhood will continue to be an important concern for the public health community.

In conclusion, this study from rural Bangladesh documented the effectiveness of HRV when delivered through a routine program in a representative low-resource population in Asia. Effectiveness was comparable to that of other live oral rotavirus vaccines, but a longer follow-up period may be needed to understand the full benefit of immunizing entire populations of infants in resource-poor settings. Although indirect protection was not observed during this 2.5-y period, such indirect protection might be observed if consistent immunization is sustained for a much longer period. Effectiveness appeared to be higher during the first year of life than the second, but delaying such an illness until later may be an important health benefit as well. Understanding the reasons for lower effectiveness after the first year is important as there may be programmatic means to address the issue of waning effectiveness. Further studies are warranted to understand the level of potential waning of protection and if additional doses can improve and extend protection.

## Supporting information

S1 Text Study protocol. (PDF)

S2 Text Western Institutional Review Board approval letter. (PDF)

S3 Text International Centre for Diarrhoeal Disease Research, Bangladesh ethical review committee approval letter. (PDF)

S4 Text Statistical analysis plan. (PDF)

S5 Text Statistical report. (PDF)

S6 Text CONSORT extension for cluster trials checklist. (DOCX)

S1 Data Study 2007–024 dataset. (XLS)

S2 Data Study 2007–024 dataset codebook. (DOCX)

## Abbreviations

ARD: acute rotavirus diarrhea
ATP: according-to-protocol
DTP: diphtheria-tetanus-pertussis
EPI: Expanded Programme on Immunization
ETEC: enterotoxigenic *Escherichia coli*
GSA: government service area
HRV: human rotavirus vaccine
icddr,b: International Centre for Diarrhoeal Disease Research, Bangladesh
ISA: icddr,b service area
Matlab HDSS: Matlab Health and Demographic Surveillance System
mITT: modified intention-to-treat
OPV: oral poliovirus vaccine
WHO: World Health Organization
