# Supplementary material for: Effectiveness of a live oral human rotavirus vaccine after programmatic introduction in Bangladesh: A cluster-randomized trial
Source: PLoS Med. 2017 Apr 18;14(4):e1002282. doi: 10.1371/journal.pmed.1002282 (PMC5395158; doi:10.1371/journal.pmed.1002282)
Supplement: S1 Text — (PDF) [file pmed.1002282.s001.pdf]

icddr,b
KNOWLEDGE FOR
GLOBAL LIFESAVING SOLUTIONS

RRC APPLICATION FORM

RESEARCH PROTOCOL NUMBER: 2007-024

FOR OFFICE USE ONLY

**RRC Approval:**	☒ **Yes /**	☐ **No**	**Date: 20.5.07**
**ERC Approval:**	☐ **Yes /**	☐ **No**	**Date: 12.6.07**
**AEEC Approval:**	☐ **Yes /**	☐ **No**	**Date:**

Protocol Title: Introduction of an oral live human rotavirus (Rotarix) vaccine in Matlab

Short title (in 50 characters including space): Rotavirus vaccine trial

Theme: (Check all that apply)

- ☐ **Nutrition**
- ☐ **Emerging and Re-emerging Infectious Diseases**
- ☐ **Population Dynamics**
- ☐ **Reproductive Health**
- ☒ **Vaccine Evaluation**
- ☐ **HIV/AIDS**
- ☐ **Environmental Health**
- ☐ **Health Services**
- ☒ **Child Health**
- ☐ **Clinical Case Management**
- ☐ **Social and Behavioural Sciences**

Key words: vaccine effectiveness, rotavirus, diarrhoea, Bangladesh

Relevance of the Protocol:
A safe and effective rotavirus vaccine is needed to reduce the enormous public health burden associated with rotavirus illness, especially in developing countries (an estimated 527,000 deaths worldwide annually). Earlier unanticipated adverse events experienced with a rhesus rotavirus vaccine have intensified efforts to develop and evaluate other vaccine candidates so that a safe and effective public health tool would become available. Licensed rotavirus vaccines are now available in the industrialized world. Evaluation of these available rotavirus vaccines is an urgent priority for Bangladesh and other developing countries with high morbidity.

Centre's Priority (as per Strategic Plan, to be imported from the attached Excel Sheet):
Priority areas: Infectious diseases and vaccine sciences, code # 4.3: Define the need for selected vaccines, e.g. hepatitis B, and evaluate promising new vaccines for enteric (rotavirus, cholera, ETEC, typhoid) and respiratory infections (H. influenzae, S. pneumoneae, viral influenza, RSV), dengue, and tuberculosis. Conduct trials of relevant new vaccines including phase 1, 2 and 3 trials.

Programmes:

- ☒ **Child Health Programme**
- ☐ **Nutrition Programme**
- ☒ **Programme on Infectious Diseases & Vaccine Science**
- ☐ **Poverty and Health Programme**
- ☐ **Health and Family Planning Systems Programme**
- ☐ **Population Programme**
- ☐ **Reproductive Health Programme**
- ☐ **HIV/AIDS Programme**

Principal Investigator (Should be a Centre's staff)
Dr. K. Zaman
Address (including e-mail address):
Scientist and Epidemiologist, PHSD,
email:kzaman@icddrb.org

DIVISION:

- ☐ **CSD**
- ☐ **HSID**
- ☐ **LSD**
- ☒ **PHSD**

Co-Principal Investigator(s): Internal Dr. Md. Yunus

**Co-Principal Investigator(s): External:**<br>**(Please provide full official address including e-mail address and Gender)**
**Co-Investigator(s): Internal:** Dr. Shams El Arifeen, Dr. Tasnim Azim, Dr. Goutam Podder, Dr. ASG Faruque, Dr Al Fazal Khan, Dr. Chandra Shekhar Das, Dr. Steve Luby
**Co-Investigator(s): External:** Dr. David A Sack, Johns Hopkins University, Baltimore<br>**(Please provide full official address including e-mail address and Gender)**
**Student Investigator(s): Internal (Centre's staff):**
**Student Investigator(s): External:**<br>**(Please provide full address of educational institution and Gender)**

Collaborating Institute(s): Please Provide full address

Institution 1

**Country**	USA
**Contact person**	David A Sack, M.D.
**Department (including Division, Centre, Unit)**	Department of International Health
**Institution (with official address)**	Johns Hopkins University, 615 N Wolfe Street / E5036, Baltimore, Maryland, 21205

Institution 2

**Country**	USA
**Contact person**	John C. Victor, PhD, MPH
**Department (including Division, Centre, Unit)**	Rotavirus Vaccine Program
**Institution (with official address)**	Program for Appropriate Technology in Health<br>1455 NW Leary Way, Seattle, WA 98107
**Directorate (in case of GoB i.e. DGHS)**	
**Ministry (in case of GoB)**	

Note: If more than 3 collaborating institutions are involved in the research protocol, additional block(s) can be inserted to mention its/there particular(s).

**Country**	
**Contact person**	
**Department**<br>**(including Division, Centre, Unit)**	
**Institution**<br>**(with official address)**	
**Directorate**<br>**(in case of GoB i.e. DGHS)**	
**Ministry (in case of GoB)**	

Population: Inclusion of special groups (Check all that apply):

Gender

- ☒ **Male**
- ☒ **Female**

Age

- ☒ **0 – 4 years**
- ☐ **5 – 9 years**
- ☐ **10 – 19 years**
- ☐ **20 – 64 years**
- ☐ **65 +**

- ☐ **Pregnant Women**
- ☐ **Fetuses**
- ☐ **Prisoners**
- ☐ **Destitutes**
- ☐ **Service Providers**
- ☐ **Cognitively Impaired**
- ☐ **CSW**
- ☐ **Others (specify)**
- ☐ **Animal**

NOTE It is the policy of the Centre to include men, women, and children in all research projects involving human subjects unless a clear and compelling rationale and justification (e.g. gender specific or inappropriate with respect to the purpose of the research) is there. Justification should be provided in the `Sample Size' section of the protocol in case inclusiveness of study participants is not proposed in the study.

Project/study Site (Check all the apply):

- ☐ **Dhaka Hospital**
- ☒ **Matlab Hospital**
- ☒ **Matlab DSS Area**
- ☐ **Matlab non-DSS Area**
- ☐ **Mirzapur**
- ☐ **Dhaka Community**
- ☐ **Chakaria**
- ☐ **Abhoynagar**

- ☐ **Mirsarai**
- ☐ **Patyia**
- ☐ **Other areas in Bangladesh**
- ☐ **Outside Bangladesh**

  Name of Country:
- ☐ **Multi Centre Trial**

(Name other countries involved):

Type of Study (Check all that apply):

- ☐ Case Control Study
- ☒ Community-based Trial/Intervention
- ☐ Program Project (Umbrella)
- ☐ Secondary Data Analysis
- ☒ Clinical Trial (Hospital/Clinic)
- ☐ Family Follow-up Study
- ☐ Cross Sectional Survey
- ☒ Longitudinal Study (cohort or follow-up)
- ☐ Record Review
- ☐ Prophylactic Trial
- ☒ Surveillance/Monitoring
- ☒ Others: Vaccine evaluation

NOTE: Does the study meet the definition of clinical studies/trials given by the International Committee of Medical Journal Editors (ICMJE)? Yes ☒ No ☐

Please note that the ICMJE defined clinical trial as "*Any research project that prospectively assigns human subjects to intervention and comparison groups to study the cause-and-effect relationship between a medical intervention and a health outcome*".

If YES, after approval of the ERC, the PI should complete and send the relevant form to provide required information about the research protocol to the Committee Coordination Secretariat for registration of the study into websites, preferably at the www.clinicaltrials.gov. It should be noted that the PI is required to provide subsequent updates of the research protocol.

Targeted Population (Check all that apply):

- ☒ No ethnic selection (Bangladeshi)
- ☐ Bangalee
- ☐ Tribal group
- ☐ Expatriates
- ☐ Immigrants
- ☐ Refugee

Consent Process (Check all that apply):

- ☒ Written
- ☐ Oral
- ☐ None
- ☒ Bengali Language
- ☐ English Language

Proposed Sample Size:

Sub-group (Name of subgroup (e.g. Men, Women) and Number

Name	Number	Name	Number
Infants born and residing in villages randomized to receive Rotarix	~4550		
Infants born and residing in villages randomized to not receive Rotarix	~4550		

Total sample size: ~9100

Determination of Risk: Does the Research Involve (Check all that apply):

- ☐ Human exposure to radioactive agents?
- ☐ Fetal tissue or abortus?
- ☐ Investigational new device? (specify:)
- ☒ Existing data available from Co-investigator
- ☐ Human exposure to infectious agents?
- ☐ Investigational new drug
- ☐ Existing data available via public archives/sources
- ☒ Pathological or diagnostic clinical specimen only
- ☐ Observation of public behaviour
- ☐ New treatment regime

**Yes** ☒	**No** ☐	**Is the information recorded in such a manner that study participants can be identified from information provided directly or through identifiers linked to the study participants?**
**Yes** ☐	**No** ☒	**Does the research deal with sensitive aspects of the study participants' behaviour; sexual behaviour, alcohol use or illegal conduct such as drug use?**

Could the information recorded about the individual if it became known outside of the research:

**Yes** ☐	**No** ☒	**Place the study participants at risk of criminal or civil liability?**
**Yes** ☐	**No** ☒	**Damage the study participants' financial standing, reputation or employability, social rejection, lead to stigma, divorce etc.?**

Do you consider this research (Check one):

☐ **Greater than minimal risk** ☒ **No more than minimal risk**

☐ **Only part of the diagnostic test**

Minimal Risk is "a risk where the probability and magnitude of harm or discomfort anticipated in the proposed research are not greater in and of themselves than those ordinarily encountered in daily life or during the performance of routine physical, psychological examinations or tests. For example, risk of drawing a small amount of blood from a healthy individual for research purposes is no greater than the risk of doing so as a part of routine physical examination".

Yes/ No

☒ ☐ **Is the proposal funded?**

If yes, sponsor Name:
(1) Rotavirus Vaccine Program (RVP), PATH, primary study Sponsor
(2) UNICEF (donating start-up funds)
(3) GlaxoSmithKline (donating vaccine)

Yes/No

☐ ☒ **Is the proposal being submitted for funding?**

If yes, name of funding agency:

Do any of the participating investigators and/or member(s) of their immediate families have an equity relationship (e.g. stockholder) with the sponsor of the project or manufacturer and/or owner of the test product or device to be studied or serve as a consultant to any of the above?

***IF YES, a written statement of disclosure to be submitted to the Centre's Executive Director*.**

Dates of Proposed Period of Support

(Day, Month, Year - DD/MM/YY)

Beginning Date : 01 May, 2008

End Date : 30 June, 2011

Cost Required for the Budget Period ($)

Years	Direct Cost	Indirect Cost	Total Cost
Year-1			
Year-2			
Year-3			
Year-4			
Year-5			
Total			

Certification by the Principal Investigator

I certify that the statements herein are true, complete and accurate to the best of my knowledge. I am aware that any false, fictitious, or fraudulent statements or claims may subject me to criminal, civil, or administrative penalties. I agree to accept the responsibility for the scientific conduct of the project and to provide the required progress reports including updating protocol information in the SUCHONA (Form # 2) if a grant is awarded as a result of this application.

Signature of PI ____________________ Date ____________

Approval of the Project by the Division Director of the Applicant

The above-mentioned project has been discussed and reviewed at the Division level as well by the external reviewers. The protocol has been revised according to the reviewers' comments and is approved.

Marge Koblinsky

Name of the Division Director **Signature** **Date of Approval**

Table of Contents

**RRC APPLICATION FORM……………………………………………………………………………**	1
Project Summary…………………………………………………………………………………..............	8
Description of the Research Project ……………………………………………………………........	10
Hypothesis to be Tested:…………………………………………………………………………........	10
Specific Aims ………………………………………………………………………………………........	10
Background of the Project including Preliminary Observations ……………………………………...	11
Research Design and Methods ………………………………………………………………………....	16
Sample Size calculations ………………………………………………………………………........	22
Facilities Available ………………………………………………………………………………………	26
Data Analysis ……………………………………………………………………………………………	27
Ethical Assurance for Protection of Human Rights …………………………………………………...	28
Use of Animals ………………………………………………………………………………………..	29
Literature Cited …………………………………………………………………………………...........	30
Dissemination and Use of Findings ………………………………………………………………...	32
Collaborative Arrangements ………………………………………………………………………........	33
Biography of the Investigators ……………………………………………………………………..	34
Detailed budget ……………………………………………………………………………………..	36
Budget Justifications ………………………………………………………………………..	37
**Appendices**	
Appendix I- Rota serotype distribution…………………………………………………………...	38
Appendix II Clinical scoring ………………………………………………………………………	39
Appendix III Vaccination Voluntary Consent Form (English) ……………………………………	40
Appendix IV Illness Voluntary Consent Form (English) ……………………………………………	42

☒ **Check here if appendices are included**

PROJECT SUMMARY

Describe in concise terms, the hypothesis, objectives, and the relevant background of the project. Describe concisely the experimental design and research methods for achieving the objectives. This description will serve as a succinct and precise and accurate description of the proposed research is required. This summary must be understandable and interpretable when removed from the main application. **(TYPE TEXT WITHIN THE SPACE PROVIDED).**

Principal Investigator: K. Zaman

Project Name: Introduction of an oral live human rotavirus (Rotarix) vaccine in Matlab

Total Budget: $ Beginning Date: 01 May, 2008 Ending Date: 30 June, 2011

A safe and effective vaccine is needed to reduce the enormous public health burden associated with rotavirus illness, especially in developing countries. About 40,000 children with rotavirus diarrhoea are treated each year at the ICDDR,B hospitals. Globally, there are an estimated 527,000 deaths annually. Unanticipated adverse events (intussusception) experienced with a rhesus rotavirus vaccine have accelerated efforts to develop and evaluate alternative vaccine candidates so that a safe and effective public health tool would become available. GSK Biologicals' live attenuated human rotavirus vaccine (HRV), Rotarix has been tested in clinical studies and was shown to be efficacious, safe and well tolerated among infants. The vaccine has been licensed in more than 80 countries, including Bangladesh.

The study will be conducted in the Matlab Health and Demographic Surveillance System (HDSS) field area of the International Centre for Diarrhoeal Disease Research, Bangladesh (ICDDR,B) to determine the population effectiveness of Rotarix in Bangladeshi children. Villages in both intervention and government comparison areas will be included in this evaluation. We propose to introduce Rotarix into half of the villages of the Matlab HDSS. In villages randomized to receive the vaccine, all eligible children will be offered Rotarix during their first two Expanded Programme on Immunization (EPI) visits, as would routinely be done if Rotarix were included in the Government EPI schedule. In villages randomized not to receive Rotarix, children will receive their EPI vaccinations exactly as they would have in the absence of this study. Administration of Rotarix will be conducted by regular EPI staff, but ICDDR,B study staff will be present to document informed consent and collect study-specific information. The Ministry of Health will be an active partner in this evaluation since they will be the agency which may follow up with any subsequent vaccine programme. Vaccination with Rotarix will be recorded on the infant's immunization card which is normally used by the EPI programme, but also on a separate study-specific data collection form.

Vaccination with Rotarix will continue from study initiation through June 30, 2011. Surveillance for rotavirus gastroenteritis will occur at Matlab Diarrhoeal Hospital and the community treatment centres of the Matlab HDSS continuously throughout the study period. Diarrheal illness information collected through surveillance will be linked to Rotarix study-specific data through the subject's HDSS identification numbers. The primary study endpoint will be the occurrence of an illness episode of acute diarrhoea, among infants and children admitted to a medical facility, determined to be caused by wild-type rotavirus found in a stool specimen. At the end of the surveillance period, rates of this primary study endpoint among age-eligible infants will be compared for villages randomized to receive Rotarix versus for villages randomized not to receive Rotarix. We expect that the rates of rotavirus diarrhoea will be significantly lower among children from the vaccinated villages.

KEY PERSONNEL (List names of all investigators including PI and their respective specialties

Name	Professional Discipline/ Specialty	Role in the Project
Dr. K. Zaman	Scientist and Epidemiologist	PI
Dr. Md. Yunus	Senior Scientist and Head, Matlab HRC	Co-PI
Dr. David A Sack	Infectious Diseases and Vaccine specialist	Co-investigator
Dr. Shams El Arifeen	Epidemiologist	Co-investigator
Dr. Tasnim Azim	Virologist	Co-investigator
Dr. Goutam Podder	Virologist	Co-investigator
Dr. ASG Faruque	Scientist	Co-investigator
Dr. Al Fazal Khan	Clinician	Co-investigator
Dr. Chandra Shekhar Das	Clinician	Co-investigator
Dr. Steve Luby	Infectious Disease and Vaccine specialist	Co-investigator

DESCRIPTION OF THE RESEARCH PROJECT
Hypothesis to be tested:

Concisely list in order, in the space provided, the hypothesis to be tested and the Specific Aims of the proposed study. Provide the scientific basis of the hypothesis, critically examining the observations leading to the formulation of the hypothesis.

When added to the Bangladesh EPI schedule, Rotarix will significantly reduce the occurrence of rotavirus diarrhoea caused by any rotavirus serotype in the population.

Specific Aims:

Describe the specific aims of the proposed study. State the specific parameters, biological functions/ rates/ processes that will be assessed by specific methods **(TYPE WITHIN LIMITS).**

Primary
To evaluate the population (overall) (Halloran, et al, 1997) effectiveness of Rotarix in reducing rates of acute diarrhoea due to rotavirus, as detected by patients admitted to a treatment facility, among all infants and young children from villages where Rotarix is introduced compared to among those from villages where Rotarix is not introduced.

Secondary
1. To evaluate the total effectiveness of Rotarix in reducing rates of acute diarrhoea due to rotavirus, as detected by patients admitted to a treatment facility, among vaccinated infants and young children from villages where Rotarix is introduced compared to among unvaccinated infants and young children from villages where Rotarix is not introduced.

2. To evaluate the indirect effectiveness of Rotarix in reducing rates of acute diarrhoea due to rotavirus, as detected by patients admitted to a treatment facility, among unvaccinated infants and young children from villages where Rotarix is introduced compared to among those from villages where Rotarix is not introduced.

3. To evaluate the direct effectiveness of Rotarix in reducing rates of acute diarrhoea due to rotavirus, as detected by patients admitted to a treatment facility, among vaccinated infants and young children from villages where Rotarix is introduced compared to among unvaccinated infants and young children from villages where Rotarix is introduced.

4. To investigate the overall, total, indirect, and direct effectiveness of Rotarix in reducing rates of acute diarrhoea due to rotavirus and requiring at least one overnight stay and rehydration therapy (equivalent to WHO plan B or C) in a treatment facility.

5. To investigate the direct effectiveness and total effectiveness of Rotarix in reducing rates of acute diarrhoea due to rotavirus, as detected by patients admitted to a treatment facility, by number of doses received.

6. To investigate the direct effectiveness and total effectiveness of Rotarix in reducing rates of all-cause acute diarrhoea, as detected by patients admitted to a treatment facility.

Background of the Project
including Preliminary Observations

Describe the relevant background of the proposed study. Discuss the previous related works on the subject by citing specific references. Describe logically how the present hypothesis is supported by the relevant background observations including any preliminary results that may be available. Critically analyze available knowledge in the field of the proposed study and discuss the questions and gaps in the knowledge that need to be fulfilled to achieve the proposed goals. Provide scientific validity of the hypothesis on the basis of background information. If there is no sufficient information on the subject, indicate the need to develop new knowledge. Also include the **significance and rationale** of the proposed work by specifically discussing how these accomplishments will bring benefit to human health in relation to biomedical, social, and environmental perspectives. **(DO NOT EXCEED 5 PAGES, USE CONTINUATION SHEETS).**

Rotaviruses, non-enveloped, double-stranded RNA viruses, infect both humans and animals and are distributed worldwide. In humans, rotavirus causes diarrhoea of varying severity ranging from mild to severe. Deaths occur from rotavirus diarrhoea when the diarrhoea is sufficiently severe to cause dehydration. Most illnesses occur in infants, although adults and the elderly may also be affected. Rotavirus is the most common cause of severe diarrhoea leading to hospital treatment in every country where etiologies have been monitored, including both developed and developing countries. Annually, rotavirus causes >25 million outpatient visits, >2 million hospitalizations, and an estimated 527,000 deaths (475,000-580,000) in children less than 5 years of age, of which deaths predominantly occur in low-income countries (WHO, 2007a).

Rotavirus has several serotypes based on G and P antigens. In most industrialized countries, G1 is the most common type by far. Other serotypes also occur and seem to vary from year to year and by geographic location. Recently, G2 has emerged as being common in Bangladesh. Infection with serotype G5 has also been recently reported from some developing countries. All major strains of pathogenic rotavirus (G1, G2, G3, G4 and G9) have been isolated in most countries. The most prevalent is G1, accounting for over 50% of rotavirus diarrhoea in Korea, Taiwan, China, Thailand and Vietnam in certain years, but there have been epidemics caused by G2 in Thailand and Vietnam, G3 in China and Taiwan, G4 in Malaysia and Thailand and G9 in Malaysia. Mixed serotype disease has also been observed in China and Vietnam (Seo et al, 2000; Tsai et al, 2000; Fang et al, 2002; Maneekarn et al, 2000; Nishio et al, 2000; Nguyen et al, 2001).

While accurate estimation of the disease burden of rotavirus in the Asia-Pacific region is difficult due to the paucity of detailed data, it is clear that rotavirus diarrhoea is widely prevalent throughout the Asia-Pacific region (Seo et al, 2000; Tsai et al, 2000; Punyaratabandhu et al, 1991; Yap et al, 1992). However, the incidence of rotavirus gastroenteritis does vary across the region. Factors which cause this variation include the quality of healthcare accessible to the population, the age group of the population at risk and the individual socio-economic status. Hence, Thailand has been found to have an incidence rate of 42% per annum of infants in an urban low income population while Malaysia has a rate of just 2.8% per annum among children from the affluent Klang valley (Punyaratabandhu et al, 1991; Yap et al, 1992). Hong Kong has been found to have an incidence of between 30 to 35% per annum among infants while Korea's incidence rate of rotavirus diarrhoea has been estimated at 13.8% per annum among children (Seo et al, 2000).

Rotavirus surveillance in Dhaka and Matlab

At ICDDR,B rotavirus accounts for about 40% of the diarrhoeal illnesses in patients under 5 years of age and is very common among those less than 2 years of age as determined by the surveillance carried out at both the Dhaka and Matlab hospitals. Deaths due to rotavirus in these settings are extremely rare because

rehydration is provided; however, in many areas of Bangladesh, adequate treatment may not be given, resulting in an estimated 15,382 rotavirus deaths annually in the country (WHO, 2007a).

Unicomb et al (1999) analyzed data from ICDDR,B hospitalized patients during 1987-97 to study the distribution of various types of rotavirus. It revealed that G4 was the most common type followed by G2, G1, G3 and G9. During 2002, G9 was one of the predominant strains isolated in the ICDDR,B (Appendix 1). G2 was the most predominant type during 2005-06.

Tables 1a and 1b show the demographic characteristics of the government comparison and intervention areas, respectively, in 2005.

Table 1a: Population characteristics of the government comparison area, Matlab HDSS, 2005.

<table>
<tr><td>Total population</td><td>112,399</td><td>Number of villages</td><td>75</td></tr>
<tr><td>Crude birth rate</td><td>23.1</td><td>Live births</td><td>2,602</td></tr>
<tr><td>Neonatal mortality rate</td><td>35.4*</td><td>Infants surviving to 4 weeks of age and who could enter into evaluation</td><td>2,510</td></tr>
<tr><td>Post Neonatal mortality rate</td><td>9.6*</td><td>Infants who could receive two doses of vaccine from the selected villages</td><td>1255</td></tr>
<tr><td rowspan="2">Child mortality rate (0-4 years)</td><td rowspan="2">60.2*</td><td>Infants who may die between 4 weeks of age and 1 year of age</td><td>24</td></tr>
<tr><td>Children from whom illnesses could be ascertained through at least 1 year of age</td><td>2486</td></tr>
</table>

* per 1000 live births

Table 1b: Population characteristics of the ICDDR,B intervention area, Matlab HDSS, 2005.

<table>
<tr><td>Total population</td><td>112,351</td><td>Number of villages</td><td>67</td></tr>
<tr><td>Crude birth rate</td><td>23.2</td><td>Live births</td><td>2,608</td></tr>
<tr><td>Neonatal mortality rate</td><td>26.5*</td><td>Infants surviving to 4 weeks of age and who could enter into evaluation</td><td>2,531</td></tr>
<tr><td>Post Neonatal mortality rate</td><td>9.6*</td><td>Infants who could receive two doses of vaccine from the selected villages</td><td>1266</td></tr>
<tr><td rowspan="2">Child mortality rate (0-4 years)</td><td rowspan="2">45.3*</td><td>Infants who may die between 4 weeks of age and 1 year of age</td><td>24</td></tr>
<tr><td>Children from whom illnesses could be ascertained through at least 1 year of age</td><td>2507</td></tr>
</table>

* per 1000 live births

Tables 2a and 2b show the numbers of diarrhoea and rotavirus admissions at the Matlab Diarrhoeal Hospital among children from the government comparison and intervention areas, respectively, over the four-year period, 2003-2006. And Table 3a and 3b show the average annual rates of rotavirus admissions

at Matlab Diarrhoeal Hospital for this same period, among children from the government comparison and intervention areas, respectively. Since most severe infections occur between the ages of 4 months and 2 years of age, vaccination should begin early in life, and immunization should be completed prior to 6 months. From a logistical standpoint, the vaccine can best be delivered if it can be included within the EPI program where the provisions are already in place for the cold chain.

Table 2a: Number of diarrhoea and rotavirus admissions to Matlab Diarrhoeal Hospital among children 0 to 23 months of age living in Matlab HDSS government comparison area, 2003-2006.

Age (months)	2003-2006	
	Diarrhoea admissions	RV admissions (%)
0-2	17	2 (12)
3-5	66	27 (41)
6-11	400	172 (43)
12-17	232	114 (49)
18-23	91	32 (35)
Total	806	347 (43)

Table 2b: Number of diarrhoea and rotavirus admissions to Matlab Diarrhoeal Hospital among children 0 to 23 months of age living in Matlab HDSS ICDDR,B intervention area, 2003-2006.

Age (months)	2003-2006	
	Diarrhoea admissions	RV admissions (%)
0-2	44	7 (16)
3-5	181	52 (29)
6-11	840	348 (41)
12-17	476	202 (42)
18-23	287	77 (27)
Total	1828	686 (38)

Table 3b: Average annual incidence of admissions for diarrhoea due to rotavirus to the ICDDR,B Matlab Diarrhoeal Hospital among children between 0 to 23 months of age living in Matlab HDSS government comparison area, 2003-2006 (based on average mid-year population). Rates are per 1000 children.

Age (month)		2003-2006	
		Average annual numbers	Average annual rate
0-11	RV admissions	50	18.4
	Birth cohort	2728	
12-23	RV admissions	34	12.3
	Birth cohort	2776	

Table 3b: Average annual incidence of admissions for diarrhoea due to rotavirus to the ICDDR,B Matlab Diarrhoeal Hospital among children between 0 to 23 months of age living in Matlab HDSS ICDDR,B intervention area, 2003-2006 (based on average mid-year population). Rates are per 1000 children.

Age (month)		2003-2006	
		Average annual numbers	Average annual rate
0-11	RV admissions	102	37.3
	Birth cohort	2725	
12-23	RV admissions	70	24.5
	Birth cohort	2847	

Rotavirus vaccines

Safe and effective rotavirus vaccines are needed to reduce the enormous public health burden associated with rotavirus illness, especially in developing countries (Miller & McCann, 2000). The large global health burden due to RV disease in both developed and developing country prompted the development of RV vaccines. Prevention by vaccination is considered to be critical for effective control of RV infection since only non-specific symptomatic therapies are available. A variety of approaches to the development of RV vaccines have been undertaken, with live oral attenuated vaccines receiving the most attention.

Previously, it was thought necessary to have a multi-valent vaccine that included all relevant serotypes. For this reason, the tetravalent rhesus reassortant vaccine, Rotashield, was developed. Rotashield was manufactured by Wyeth-Lederle and was licensed in the United States of America (USA) in 1998 and granted a marketing authorization for Europe in 1999 but was withdrawn from the market in 1999 due to an increased risk of intussusception (IS) shortly after its administration (Anonymous, 1999; Murphy et al, 2001). It was not clear, however, that a vaccine needed to be multi-valent. Based on studies of the natural history of rotavirus infections, a first infection is generally the most severe, regardless of serotype, suggesting that there is some heterotypic protection, at least against severe disease. Secondly, since rotavirus infection occurs commonly, subsequent rotavirus infections increase serotype-specific and heterotypic protection. Thus, it was believed that if the first rotavirus infection occurs with an attenuated vaccine strain, subsequent infections with virulent wild-type rotaviruses will not cause the severe life-threatening diarrhoea that now occurs.

GlaxoSmithKline Biologicals' (GSK) rotavirus vaccine, Rotarix, is a monovalent vaccine based on a human rotavirus (HRV) strain 89-12 belonging to the serotype G1P1A and genotype [P8] originally obtained from the stool of a 15-month old infant with a mild RV diarrhoea in December 1988. Rotarix is a lyophilized HRV intended for oral administration after reconstitution with buffer. Rotarix differs from Rotashield, because it is based on a human strain, whereas Rotashield was based on a rhesus strain. There are major differences in terms of biological properties and clinical symptoms between animal (rhesus) and human RV strains, while only minor differences are expected between the attenuated RIX4414 HRV strain and the wild-type HRV. HRV has not been associated with IS in infants. The most powerful evidence refuting a link between HRV infection and IS is the absence of an increase in IS rates during the sharply defined winter RV epidemics that occur in temperate climates (Rennels et al, 1998). The RIX4414 human strain in Rotarix is attenuated and the attenuation might further decrease the likelihood of IS. Administration of Rotarix does not induce a viral exposure that would otherwise not occur, in contrast with the administration of the rhesus rotavirus vaccine which represents a virus that would not normally infect children.

Clinical results with Rotarix

A large multinational trial was conducted in 11 Latin American countries and Finland to evaluate the safety and efficacy of Rotarix. A total of 63,225 infants was randomized to receive vaccine or placebo. The efficacy of the vaccine against severe rotavirus gastroenteritis and against rotavirus associated hospitalization was 85%. Hospitalization for diarrhoea of any cause was reduced by 42%. The vaccine was not associated with an increased risk of intussusception (Ruiz-Palacios et al, 2006). WHO's Global Advisory Committee on Vaccine Safety also reviewed and was "reassured" by Rotarix postmarketing surveillance data which show, with about 5 million doses distributed, no evidence of an excess incidence of intussusceptions (WHO, 2007b). Finally, evidence also suggests the possibility of heterotypic protection including G9 serotype (Ruiz-Palacios et al, 2006; Vesikari, 2007). Rotarix is currently licensed in more than 80 countries, including Bangladesh.

How does this differ from a traditional double-blind, individually-randomized clinical trial?

This evaluation will provide data on the population effectiveness of the vaccine when used in a routine, but high quality, EPI programme. Thus, it will add information which will be most helpful to policy makers when they are deciding how to allocate funds for public health programmes. Furthermore, since this evaluation will be based in Matlab which has pioneered many public health interventions of global importance, the high quality of the study data will add credibility to the usefulness of the vaccine in impoverished countries like Bangladesh. With a birth cohort of about 3.5 million children, the EPI programme in this country must carefully allocate resources to insure maximum cost effectiveness of its scarce resources.

Also, introduction of the vaccine into a pilot area like this one will provide experience with the vaccine in a "real-world" setting and will provide practical experience for the Ministry in actually using the vaccine. Many operational lessons will be learned on vaccine delivery in the villages of Bangladesh. If the Ministry does decide to begin including the vaccine in its EPI programme, it can use these lessons for a rapid scale up. Finally, because of the comprehensive nature of the Matlab HDSS, there is likely to be further evaluations of the public health benefits of the vaccine which are not now anticipated. In fact the publications cited on measles, tetanus and the routine EPI vaccines were evaluations of programmes in which the outcomes were not anticipated at the time of the intervention. (Black et al, 1980; Breiman et al, 2004; Koenig et al, 1998). Because of high quality of the routine data collection and the longitudinal data collection over many years, these very important studies resulted in major policy decisions.

ICDDR,B experience in conducting rotavirus vaccine trials

Before it was withdrawn from the market, a safety and immunogenicity study was conducted at Matlab with Rotashield (RRV-TV) vaccine during 1998-9. The total number of infants participated in the trial was 120. The vaccine was found to be safe and immunogenic (Bresee et al, 2001). Currently in Matlab HDSS population, in the intervention area which neighbors the government comparison area, ICDDR,B investigators are conducting an individually-randomized evaluation of the Merck live oral rotavirus vaccine, RotaTeq. The total number of infants from the intervention area of the Matlab site participating in that field trial is approximately 1200, with enrolment having closed in January, 2008.

Several trials were conducted with Rotarix in urban Dhaka at Mirpur among toddlers and infants during 2002-06 to assess the safety and immunogenicity of the vaccine. The study site is about 7 kilometer northwest of ICDDR,B. A total of 730 infants and toddlers were enrolled in these studies. The vaccine was found to be safe and reported solicited symptoms were general mild and of short duration. The percentage of subjects reporting each solicited symptoms (diarrhoea, fever, irritability, loss of appetite, vomiting) or unsolicited symptoms (pneumonia, otitis media, skin infections etc.) within the 8-day follow-up period after Rotarix or placebo dose were similar in vaccine or placebo group. Two doses of Rotarix have a good immunogenic profile in children in Bangladesh. Anti-RV IgA seroconversion rate in the Rotarix group where OPV was administered concomitantly was 57% (95% CI: 44% to 68%) compared with 67% (95% CI: 54% to 78%) when Rotarix was administered alone. The seroconversion rate in the pooled placebo group was 19% (95% CI: 10% to 30%). No significant difference was observed in OPV seroprotection rates between the vaccine groups (Zaman et al, 2007).

RESEARCH DESIGN AND METHODS

Describe in detail the methods and procedures that will be used to accomplish the objectives and specific aims of the project. Discuss the alternative methods that are available and justify the use of the method proposed in the study. Justify the scientific validity of the methodological approach (biomedical, social, or environmental) as an investigation tool to achieve the specific aims. Discuss the limitations and difficulties of the proposed procedures and sufficiently justify the use of them. Discuss the ethical issues related to biomedical and social research for employing special procedures, such as invasive procedures in sick children, use of isotopes or any other hazardous materials, or social questionnaires relating to individual privacy. Point out safety procedures to be observed for protection of individuals during any situations or materials that may be injurious to human health. The methodology section should be sufficiently descriptive to allow the reviewers to make valid and unambiguous assessment of the project. **(DO NOT EXCEED TEN PAGES, USE CONTINUATION SHEETS).**

Experimental Design and Methods

2.1 Study Area

The study will be conducted in rural Bangladesh at Matlab, where the International Centre for Diarrhoeal Disease Research, Bangladesh (ICDDR,B) has been maintaining a field research project since 1963. Matlab is a low-lying riverine area which lies 45 km south east of Dhaka, the capital of Bangladesh. The principal occupations in the Matlab area are farming and fishing. Since 1966 a Health and Demographic Surveillance System (HDSS), which consists of regular cross-sectional censuses and longitudinal registration of vital events, has been maintained in the area (ICDDR,B, 2006). A central treatment facility, staffed by physicians and paramedics provides free therapy for 12,000-15,000 diarrhoea patients a year. A Maternal, Child Health & Family Planning “intervention” program (MCH-FP) has been in operation for half of the population of the HDSS area since 1978 and intensive research has been conducted in this population (Fauveau, et al, 1988). The other half serves as a comparison area where regular government health care facilities are available. Each community health research worker (CHRW) in the “intervention area” covers a population of about 2700. She visits each household every two months and is responsible for recording of vital events, collecting health information about diarrhoea, acute respiratory infections and breast feeding, immunization to children, referral of severely sick children and mothers etc. The CHRW also provides services, including EPI to mothers and children 4 days in a month through fixed site clinic located in her house. Each CHRW in the “government comparison area” covers a population more than two times larger than that covered by a CHRW in the intervention area. CHRWs in the government comparison areas are mainly responsible for recording of demographic events and collecting of health information. The EPI coverage in the villages is more than 90%.

2.1.1 Advantages of Matlab as study site

- Ongoing HDSS in a large population for more than 40 years. Pregnant women and newborns are identified through HDSS. HDSS data set includes many other variables e.g. SES, family structure, GIS etc.
- Matlab hospital and community treatment centres are well known to the population, and nearly all severe diarrhoea patients come to one of these facilities. Patients with mild diarrhoea are treated with ORS in the home.
- Laboratories in Matlab and Dhaka can detect rotavirus. In addition, laboratories in Dhaka have skills and experience in carrying out serotyping and serology.
- Nearly all (about 95%) of children are given standard EPI vaccines.
- Well established infrastructure for surveillance and referral facilities.
- Several vaccine trials (e.g. parenteral and oral cholera vaccine, rotavirus vaccine, shigella vaccine etc.) have been conducted at Matlab and medical staffs are familiar with conducting such trails.

2.2 Summary of Study Design

This trial will determine the population effectiveness of Rotarix in Bangladeshi infants. The study will be conducted in both the government comparison area and the intervention area of the Matlab HDSS. To carry out the evaluation, we will introduce the Rotarix vaccine into half of the villages of the HDSS area. In villages randomized to receive Rotarix, the study will be explained to parents and if they consent to their child's participation and the child is eligible, the child will receive two doses of Rotarix during their first two EPI visits (scheduled for 6 and 10 weeks of age), if possible, as would routinely be done if Rotarix were included in the government programme. Administration of Rotarix will be conducted by regular EPI staff, but ICDDR,B study staff will be present to document informed consent and collect study-specific information. Vaccination with Rotarix will be recorded on the infant's immunization card which is normally used by the EPI programme, but also on a separate study-specific data collection form. Participation in the study will not affect a subject's receipt of other routine childhood immunizations or other medical care. In villages randomized not to receive Rotarix, children will receive their EPI vaccinations exactly as they would have in the absence of this study.

Surveillance for will be conducted at the Matlab Diarrhoeal Hospital and the two community treatment centres at Nayergaon and Kalirbazaar, which facilitate quick and easy accessibility of treatment for life-threatening diarrhoeal diseases from the HDSS area. The main study outcome will be episodes of rotavirus diarrhoea among children admitted to Matlab Diarrhoeal Hospital or either of the two community treatment centres (Nayergaon and Kalirbazaar). Study personnel will record the child's symptoms and clinical history, and collect a fecal specimen for laboratory testing for rotavirus. The identity of the child will be confirmed through the HDSS subject identification number system. All patients coming to the Matlab Diarrhoea Hospital and Nayergoan community treatment centre from these villages are already included in the Matlab diarrhoea surveillance system, and faecal samples are routinely tested for rotavirus and other pathogens, with results linked to demographic and health databases of the HDSS. Kalirbazaar treatment centre is not currently included in the current surveillance system, but we will establish surveillance at this centre during this study. For enrolled participants who present to one of the treatment facilities, illness information and test results will be linked to HDSS demographic data and study-specific data collected at enrolment through the subject's HDSS number. For residents not enrolled who present to a treatment facility, illness information will be linked only to HDSS demographic data, as is already being done as part of surveillance activities.

Rates of acute gastroenteritis caused by rotavirus and other agents among age-eligible infants will be compared for infants (vaccinated and unvaccinated) from villages randomized to receive Rotarix versus for those (unvaccinated) from villages randomized not to receive Rotarix. We expect that the rates of diarrhoea will be significantly lower among children from the vaccinated villages, but that rates of diarrhoea caused by other pathogens may be similar. (This second analysis - a "control analysis" - will be helpful to verify a lack of bias in health seeking behaviours since one would expect that cholera, ETEC, Salmonella and Shigella gastroenteritis rates would be similar in the two groups.). These cases will be detected from all patients of all ages who come to the Matlab Diarrhoeal Hospital or the community treatment centres (Nayergaon or Kalirbazaar), but the primary analysis will include only those rotavirus diarrhoea cases among children who had been age-eligible for vaccination in their respective villages.

Although not a blinded evaluation, the introduction of bias will be reduced in several ways. First, the vaccine teams located in the EPI centres that are completely separate from the clinical settings where clinicians will be conducting surveillance. Second, the clinicians are separated from those scientists conducting laboratory testing. Third, laboratory assays for rotavirus will be carried out in the laboratory in Dhaka using coded specimens and are not conducted at the time of illness (since they have little

clinical value). Although a parent could inform the clinician as to his/her child's participation in the study, this is believed to be an unlikely or important source of bias for two reasons. One, most rotavirus gastroenteritis is not seen until later than 6 months after an infant's second dose of Rotarix, and there will be no active follow-up among parents after last receipt of Rotarix. Thus, there is a reduced likelihood that parents will remember that their child had received a vaccination targeted specifically against one type of severe diarrhoea. Second, Matlab Diarrhoeal Hospital and the community treatment centres have been caring for severe diarrhoea for many years and the entire community is already accustomed to attending the facilities for such illnesses. Thus, there is a reduced likelihood parents of vaccinated and unvaccinated infants are going to have differential diarrhoeal illness treatment-seeking behaviors.

Again, the study will be conducted in both the government comparison area and the intervention area of Matlab HDSS. Enrolment will begin in the government comparison area no later than May 1, 2008. Enrolment will begin in the intervention area April 1, 2009. Surveillance for rotavirus diarrhoea will occur continuously and concurrently to enrolment activities. Enrolment and surveillance in both areas will cease on June 30, 2011.

By in January 1, 2009, it is expected that less than 10% of children less than 2 years of age in the intervention area will have received Rotateq in the Merck trial. Nonetheless, we will stratify randomization within the intervention area by rates of participation in that trial for each village. Additionally, because we will have the full dataset for Matlab for the Rotateq trial, we will be able to analyze any impact of that Rotateq trial on the Rotarix evaluation within the intervention area.

2.3 Study Methods

2.3.1 Randomization Procedure

There are 75 villages in the government comparison area and 67 villages in the intervention area. The villages in each area will be randomized separately. Infants in half of the villages in each area will be eligible to receive Rotarix during their EPI doses. In each area, villages will be randomly selected for Rotarix introduction after pre-stratifying on village size and baseline hospitalization rates/numbers for diarrhoea at the Matlab Diarrhoeal Hospital and community treatment centres during 2005 and 2006. Within the intervention area, strata will also include participation and participation rates in the previous Rotateq trial. Hospital charts for diarrhoea admissions will be reviewed to determine the number/rates of hospitalizations from those villages. The villages will be arranged sequentially based on diarrhoea admission number/rates to the Matlab and Nayergaon and Kalirbazaar treatment centres. For each stratum of villages, half of the villages will be randomly selected for introduction of Rotarix and the other half will for no introduction of Rotarix.

2.3.2 Inclusions and exclusions

Inclusions

All subjects receiving Rotarix must satisfy the following criteria at study entry:

- A male or female infant at least 6 weeks of age and no older than 20 weeks of age at the time of first dose of Rotarix. *(Note: The Rotarix course is 2 doses. The first dose may be administered from the age of 6 weeks. There should be an interval of at least 4 weeks between doses. The vaccination course should preferably be given before 16 weeks of age, but must be completed by the age of 24 weeks.)*
- An infant whose parent or guardian's primary residence, at the time of first EPI vaccinations, is a village selected to receive Rotarix.

- Written informed consent obtained from the parent or guardian of the subject, prior to the subject's first study vaccination.

Exclusions

- Hypersensitivity to the active substance or any component in the vaccine (please see Rotarix composition).
- Hypersensitivity after previous administration of rotavirus vaccines.
- Previous history of intussusception.
- Subjects with uncorrected congenital malformation of the gastrointestinal tract that would predispose for intussusception.
- Infants with known or suspected immunodeficiency.
- Administration of Rotarix should be postponed in subjects suffering from an acute severe febrile illness *only if that illness also results in postponement of other EPI vaccinations* (temporary exclusion only).

2.3.3 Size of study population

In 2005, there were 2,602 and 2,608 births in the government comparison and intervention areas, respectively. Of these 2510 and 2531 survived (Table 1a and 1b.) (ICDDR,B, 2006). Based on this information, we estimate that during the study period, a similar rate of births and surviving infants will be eligible for enrolment. Thus, in accordance with enrolment plans in each area (as specified above in section 2.2), approximately 6197 infants will be eligible for vaccination with Rotarix and 6197 will be among those in villages where Rotarix is not introduced.

2.3.4 Study Vaccine

The vaccine to be used has been developed and manufactured by GSK Biologicals.

Table 4: Rotarix Formulation and Presentation

Study Vaccine	Formulation	Presentation	Vol.
Rotarix	RIX4414 HRV strain $10^{6.5}$ CCID50 Dulbecco's Modified Eagle Medium (DMEM) 3.7 mg<br>Sucrose 9 mg<br>Dextran 18 mg<br>Sorbitol 13.5 mg<br>Amino acids 9 mg	Lyophilized vaccine in monodose glass vial. Diluent (calcium carbonate buffer) supplied separately.	1 ml

Table 5: Rotarix Dosage and Administration

Visits	Vaccination	Dose	Vaccine	Route
1, 2	Rotavirus vaccine<br><br>Recommended EPI vaccine	1<br><br>1	Rotarix<br><br>BCG, DPT, Polio and Hepatitis B	Oral<br><br>Parenteral or oral

2.3.5 Procedure for administering Rotarix

Having consent from the parents, the lyophilized vaccine will be resuspended in diluent in the provided dispette and 1 ml of the solution will be administered orally. Two doses of rotavirus vaccine will be given, preferably at 4 week intervals, to coincide with a child's first and second routinely scheduled health care visits for immunization (i.e. DPT and polio). These will be recorded in the EPI cards, as well as on study-specific data collection forms. All vaccines will be given by the regular EPI staff. Vaccinations with Rotarix will be documented by ICDDR,B staff.

Should a subject regurgitate or vomit after vaccination with Rotarix, a single replacement dose of Rotarix will ***not*** be administered at the visit.

2.3.6 Storage and Handling of Rotarix

In the government comparison and intervention areas, Rotarix will be stored with and handled identical to other EPI vaccines distributed in the respective area. In the government comparison area, EPI vaccines are stored at the government's health complexes in Matlab Upazila of Chandpur District or in Daudkandi Upazila of Comilla District, the two district Upazilas covering the government comparison area of the HDSS. In the intervention area, EPI vaccines are at ICDDR,B's facilities in Matlab.

Rotarix should be kept in the refrigerator (+2°C to +8°C / 36°F to 46°F) and must not be frozen. Storage temperature will be monitored as per the standard operating procedure for other EPI vaccines in the area.

2.3.7 Surveillance for rotavirus gastroenteritis

Surveillance for rotavirus diarrhoea is currently ongoing as part of diarrhoea surveillance through the Matlab Diarrhoeal Hospital and Nayergaon community treatment centre. Kalirbazaar treatment centre is not currently included in the current surveillance system, but we will establish surveillance at this centre during this study. All children from the Matlab HDSS who present with acute gastroenteritis have stool specimens collected for testing for rotavirus by EIA. Additionally, all children have illness information recorded on a standard format Diarrhoea Treatment Record which contains information on length of illness, dehydration status, associated symptoms, outcome of illness and lab results. [This record contains sufficient clinical data to calculate a "Vesikari score" for most illnesses (Ruuska and Vesikari, 1990; See Appendix 2). However, a Vesikari score will not be this study's primary endpoint. Although most cases are discharged after complete recovery of diarrhoeal illnesses, there may be few cases in which the episode has not resolved fully at the time of discharge. In order to treat subjects from vaccinated and unvaccinated villages equivalently, there will be no home visits to obtain final outcomes in order to have all necessary information to score their illness according to the Vesikari score.] As is currently being done as part of disease surveillance in the hospital, when a child is admitted for acute gastroenteritis, every attempt will be made to identify the child while admitted in order to determine his/her HDSS number and link the child to his/her village of residency (and vaccination eligibility in this study).

2.3.8 Definitions:

- Diarrhoea: three or more looser than normal stools in a 24 hour period.
- Acute rotavirus diarrhoea: an acute diarrhoeal illness episode in which rotavirus is identified in a stool sample collected as soon as possible but no later than 7 days after admission to the medical facility.

- Follow-up period: all subjects will be eligible for the surveillance follow-up from 6 weeks of age until the selected cut-off date for surveillance, currently June 30, 2011.
- ***Primary study endpoint***: acute rotavirus diarrhoea occurring from 6 weeks of age to the end of the follow-up period in subjects who resided in randomized villages during the period when they were age-eligible to have received Rotarix.
- Fever: rectal temperature ≥ 38°C

2.3.9 Laboratory assays

Stool samples collected during episodes of gastroenteritis seen at the Matlab Diarrhoeal Hospital or Nayergaon or Kalirbazaar Community Treatment Centres will be tested as follows:

- Stool samples will be tested for RV using ELISA methods at the Virology Laboratory of the ICDDR,B. If RV is detected, RT-PCR will be done to determine the G type (G1, G2, G3, G4, G5, G8 and G9) and P type (P4, P6, P8, P9 and P10).
- Stool samples will also be tested at the Virology Laboratory of ICDDR,B for the presence of selected bacterial and parasitic pathogens (routine ova and parasite exam and culture of the stool for *Shigella spp.*, *Salmonella spp.* and *Vibrio cholerae*-01, and enterotoxigenic *E coli* using standard methods (WHO, 1987).

2.3.10 EPI centres

The GoB carries out EPI vaccination activities in the government comparison area through EPI outreach centres situated in convenient parts of each village, such as a union parishad office room, school, house of a community member, etc. Each centre covers a population of about 1000 and is run by a health assistant. Vaccinations are given monthly by the centres (Sunday through Thursday). The outreach centres receive vaccines from their respective Upazila Health Complex in the morning of the vaccination day. Similarly they return the vaccines to the Upazila Health Complex at the end of the EPI session. Because villages in the government comparison areas vary in size, some villages may be covered by more than one EPI centre. However, no EPI centre covers more than one village in the comparison area.

ICDDR,B carries out EPI vaccination activities in the intervention area. As mentioned in section 2.1, each CHRW runs a fixed site clinic (FSC) located in her house to provide health services to mothers and children <5 years of age once in a week. In alternate weeks EPI vaccines are provided. Here, one fixed site clinic may cover more than one village (total 67 villages to be covered by 41 CHRWs), and some villages may be covered by more than one FSC. In this study, because randomization will occur among village clusters, we will develop a randomization and vaccination strategy that assures that Rotarix given in intervention areas is given only to infants residing in villages randomized for introduction of Rotarix. The strategy will be designed to reduce the risk of "contamination" of parents of study participants.

2.3.11 Implementation strategy

GSK will donate study vaccine, Rotarix. Study vaccine will be administered from regular EPI outreach centres by the staff who normally conduct these clinics. ICDDR,B staff will document vaccine administration. All vaccines will be recorded in the child's immunization card and on study-specific data collection forms. At the time of EPI vaccination, all children will be identified through unique HDSS number. Field staff from ICDDR,B will identify these subjects and record HDSS identification numbers (CID- Current identification number and RID- Registration identification number) on the study-specific

data collection forms. These numbers will help to identify these children when they will attend Matlab or community treatment centres. If the infant is very young and has not yet been assigned the unique HDSS numbers, the HDSS numbers of their mother and the infant's birth date will be recorded and the infant's HDSS can be assigned at a later time, based on the mother's information.

2.3.12 Study Schedule

Detailed description of procedures to be performed during each visit is presented below.

Visit 1: Dose 1 of Rotarix (from 6 to 20 weeks of age with first doses of EPI vaccines.)

- Written informed consent obtained from the parent/guardian of the subject.
- Check age.
- Check inclusion and exclusion criteria
- Study vaccination: oral administration of first dose of Rotarix.
- Completion of study-specific data collection form.
- Routine EPI vaccinations.

Visit 2: Dose 2 of Rotarix (at least 4 weeks after dose 1 but no later than 24 weeks of age)

- Check age and time since first dose of Rotarix.
- Check inclusion and exclusion criteria
- Study vaccination: oral administration of second dose of Rotarix.
- Completion of study-specific data collection form.
- Routine EPI vaccination

2.3.13 Outline of the study:

- Study site —Matlab HDSS, Bangladesh
- Subjects in clusters (villages) randomized to receive study vaccine will receive 2 doses of Rotarix.
- Rotarix will be administered concomitantly with first two routine EPI vaccinations according to local recommendation (scheduled for 6 and 10 weeks of age).
- Vaccine is given with calcium carbonate buffer; total dose of vaccine and buffer is 1 ml. Vaccination will be done by normal EPI staff.

2.3.14 Sample size calculations

The primary study endpoint will be admission for rotavirus gastroenteritis (see definitions, section 2.3.8). From 2003 to 2006 the average annual rotavirus admission rate to the Matlab Diarrhoeal Hospital among infants 0 to 11 months of age from the government comparison area was about 18.5 per 1000 and among infants 12 to 23 months of age it was 12.3 per 1000 (Table 3a); among infants 0 to 11 months of age from

the intervention area it was 37.3 per 1000 and among infants 12 to 23 months of age it was 24.5 per 1000 (Table 3b). Because we will conduct surveillance among age-eligible infants continuously from study initiation, infants enrolled early will fall under longer surveillance while infants enrolled later will fall under brief surveillance (for example, only a few months). Additionally, because enrolment and surveillance for rotavirus diarrhoea will occur in the government comparison area and intervention area along different timelines, we used modeling to estimate a total study-period rotavirus diarrhoea hospitalization rate of 33 per 1000 infants (3.3%) (data not shown). With inclusion of infants admitted for rotavirus gastroenteritis at the Nayergaon and Kalirbazaar Community Treatment Centres, this rate may be slightly higher at 3.5%.

Because this is a cluster-randomized design, we must account for the intracluster (intra-village) correlation of acute rotavirus gastroenteritis. This value is not currently available for the study population. However, given the ubiquitous nature of rotavirus, we expect this value to be low (we assume 0.02). Additionally, because we predefine clusters as villages for this study, we cannot set the cluster sizes to be equal; villages from the government comparison and intervention areas vary substantially in size (numbers of annual births). According to the timelines for enrolment for the 142 villages in from each area, the average cluster size is expected to be 65 with a coefficient of variation of cluster sizes is 0.96. Factoring in these values, along with the intracluster correlation coefficient, into the calculation of the design effect for a cluster-randomized trial (Eldridge, 2006), we calculate a design effect of 3.48.

We calculate sample size and power based upon a fixed number of events design and conditional on the total number of cases observed (Blackwelder, 1993; Chan 1998). The number of cases in each group will be assumed to follow a Poisson distribution, with parameters λ_V for a vaccine villages and λ_U for the unvaccinated villages. Under this assumption, the number of vaccine cases, x_V, has a binomial distribution (X, P) conditional on X, the total number of cases, with $P = \lambda_V/(\lambda_V+\lambda_U)$. For the primary objective, we assume 50% vaccine population (overall) effectiveness. With no design effect (DE=1), observation of at least 77 admissions for rotavirus gastroenteritis among all age-eligible infants from vaccine and unvaccinated villages would ensure that the study has a minimum power of 80% to rule out a lower bound of 0% overall effectiveness. However, with a design effect of 3.48, a minimum of 268 admissions for rotavirus diarrhoea must be observed. Assuming a 3.5% total study-period attack rate in the unvaccinated villages, 268 admissions translates into an estimated minimum sample size of 10,210 infants (5105 in each group, vaccinated villages and unvaccinated villages) (Table 6).

Table 6: Total sample size estimates to detect a reduction in population incidence of rotavirus diarrhoea hospitalizations for different population effectiveness and baseline incidence rate assumptions, different design effect (DE) values and different numbers of required endpoints (type II error of 5%, 2-sided and 80% power).

**DE=3.48**	**Vaccine Effectiveness**			
	**50%**	**60%**	**70%**	**80%**
**Incidence***	**Required number of outcomes**			
	**268 cases**	**164 cases**	**104 cases**	**59 cases**
4.00	8937	5846	4016	2464
3.75	9528	6229	4287	2631
**3.50**	**10210**	6675	4594	2819
3.25	10997	7190	4942	3035
3.00	11909	7788	5352	3285

*RV admission rate per 100 children

(Assumes 1:1 randomization. Half of these totals sample sizes may receive Rotarix.)

During the planned course of the study (through June 2011), it is estimated that a total of approximately 12,394 infants will be born into both groups (6197 per group, section 2.3.3). While this estimated total study population is higher than that required to achieve 80% power (10,210), if population effectiveness or the underlying attack rate of were lower than 50% or 3.5%, respectively, the study would quickly become underpowered.

2.3.15 Review

The protocol will be reviewed for scientific quality by the Research Review Committee (RRC) of ICDDR,B and the clinical study team of the primary funder, the Rotavirus Vaccine Program (RVP) at PATH. The RRC of ICDDR,B is composed of 15 members, clinicians, epidemiologists, social scientists, laboratory scientists, and demographers/population scientists from both within and outside the centre, and reviews all scientific research proposals of the centre, evaluates their scientific merit, competence of the Investigators, and relevance to the Centre's objectives and priorities.

It will also be reviewed and approved by the Ethical Review Committees (ERCs) of ICDDR,B and PATH prior to starting the study. The ICDDR,B ERC is a recognized committee for review of research protocols involving humans and has a Multiple Project Assurance (MPA) with a US agency (USAID) and a FWA with the US Government (FWA # 00001468). RVP's home institution's ERC (the PATH Human Subjects Protection Committee) currently defers all vaccine trial review to the Western Institutional Review Board (WIRB) in the United States.

In addition, a scientific Steering Committee will provide overall scientific and operational advice for the study. The Steering Committee will consist of representatives of the ICDDR,B, PATH, UNICEF, the Government of Bangladesh and GSK.

Adverse events brought to the attention of investigators will be reported to ethical review committees according to committee guidelines. Additionally, serious adverse events which are judged by the investigator to be at least possibly related to receipt of Rotarix will be reported spontaneously to GSK, Bangladesh.

2.3.16 Informed consent

The parents or guardians of the subjects receiving Rotarix will sign a consent form informing them of the nature of the study, its rationale, and its risks and benefits. The informed consent document will embody the elements of the consent as described in the Declaration of Helsinki and the ICH Harmonized Tripartite Guidelines for Good Clinical Practices. The investigator or designate will describe the protocol to parents of potential subjects face-to-face. The subject information and consent form may be read to the parents of potential subjects, but in any event, the investigator will give the parents ample opportunity to inquire about the details of the study and ask questions before signing the consent form.

This study's objectives require comparing rates of diarrheal admissions among infants from villages where Rotarix is introduced to those among infants from villages where Rotarix is not introduced. In order to analyze, for the purposes of this study, data on diarrheal illnesses occurring among those not yet consented into the Rotarix study, informed consent will be requested from parents or guardians of admitted infants.

2.3.17 Approval by National Drug Administration

The protocol will be reviewed and approved by the National Drug Administration of Bangladesh prior to initiation of the study.

2.3.18 Expected outcomes and timeframe

The study will be conducted in both the government comparison area and the intervention area of Matlab HDSS. Enrolment will begin in the government comparison area no later than May 1, 2008. Enrolment will begin in the intervention area April 1, 2009. Surveillance for rotavirus diarrhoea will occur continuously and concurrently to enrolment activities. Enrolment and surveillance in both areas will cease on June 30, 2011. Estimation of overall vaccine effectiveness for reducing rotavirus diarrhoea among children who had been age-eligible for vaccination during study enrolment will be the primary objective. Demonstrating statistically significant vaccine effectiveness will provide important data for decision-making to the GoB Ministry of Health and other countries world-wide. Additionally, at the end of the study, infants in all villages in Matlab will be offered Rotarix for a period of at least three years.

2.3.19 Previous experience of the laboratory in the proposed field of research

ICDDR,B scientists have conducted a variety of studies to define the epidemiology and microbiology of rotavirus infections. The Centre also conducted several phase II safety and immunogenicity studies with rhesus rotavirus tetravalent vaccines (RRV-TV) and Rotarix at Matlab and in urban Dhaka. The Centre has a long history of studies of prevention of diarrhoeal diseases including rotavirus as well as cholera, typhoid, Shigella, and ETEC. Dr. Sack has studied the vaccine based on the HRV strain 89-12 in safety, immunogenicity and efficacy studies in the US.

2.3.20 Site Monitoring Plan

Site monitoring by the investigator will be conducted to ensure that the human subject protection, study protocol implementation, laboratory, study vaccine administration, and data collection and analysis procedures and processes are of high quality. A separate monitoring plan document will be developed to describe exactly who will conduct monitoring, at what frequency monitoring will be done, and what level of detail monitoring will be conducted. The monitoring plan will include the number of subject case report forms to be reviewed, which/what proportion of data fields will be monitored, who will be responsible for conducting monitoring visits, and who will be responsible for ensuring that monitoring findings are addressed.

2.3.21 Data Handling and Management

All data handling and management will be the responsibility of the investigator. A data management plan document will be developed to describe exactly the steps to be taken to assure that the data collected are accurate, consistent, complete, and reliable. The descriptions will include reference to source documentation, CRFs, instructions for completing forms, data handling procedures, and procedures for data monitoring. Procedures will also be specified for data handling at each the clinical site, the laboratories, and the data management center.

Facilities Available

Describe the availability of physical facilities at the place where the study will be carried out. For clinical and laboratory-based studies, indicate the provision of hospital and other types of patient's care facilities and adequate laboratory support. Point out the laboratory facilities and major equipments that will be required for the study. For field studies, describe the field area including its size, population, and means of communications. **(TYPE WITHIN THE PROVIDED SPACE).**

The International Centre for Diarrhoeal Disease Research, Bangladesh (ICDDR,B) has large multi-disciplinary international and national scientific research staff. Existing field, hospital, laboratory and office facilities will be used for this study. ICDDR,B scientists have conducted a variety of vaccine studies including rotavirus.

Field site

The study will be conducted in rural Matlab. ICDDR,B has been maintaining a field research centre at Matlab for about forty years. Due to the presence of ongoing health and demographic surveillance system (HDSS), clinic and laboratory facilities, effective referral systems and well-established infrastructure at Matlab, it offers excellent research facilities for this study. The HDSS is a regularly updated information system on the approximate population of 220,000.

Laboratory facilities

Existing laboratory facilities in ICDDR,B will be used.

Data Analysis

Describe plans for data analysis. Indicate whether data will be analyzed by the investigators themselves or by other professionals. Specify what statistical software packages will be used and if the study is blinded, when the code will be opened. For clinical trials, indicate if interim data analysis will be required to monitor further progress of the study. **(TYPE WITHIN THE PROVIDED SPACE).**

Before carrying out the primary analysis, baseline characteristics of the subjects receiving vaccine and not receiving vaccine will be determined using HDSS information.

The primary analysis will be to evaluate the vaccine's population effectiveness against rotavirus diarrhoea as detected at the hospital or treatment centre during the follow-up period. The surveillance period will be a minimum from a subject's age of 6 weeks through June 30, 2011. Analyses will compare rates of rotavirus diarrhoea among children from vaccine villages and villages without vaccination, accounting for the cluster randomized design. Subjects with rotavirus diarrhoea will be counted only during their first rotavirus hospitalization episode (e.g. second rotavirus episodes will be monitored but will not be included in the primary analysis). All first rotavirus diarrhoea episodes will be included in the primary analysis regardless of serotype. Only primary endpoints occurring from 6 weeks of age to the end of the study surveillance period in children who were age-eligible for vaccination with Rotarix during the study will be counted in the primary analysis. Vaccine population effectiveness (with 95% CI) will be calculated as [1 - (incidence rate of rotavirus among vaccine villages/incidence rate among the villages with out vaccine)] x 100.

Secondary analyses will include evaluation of Rotarix in preventing severe rotavirus diarrhoea and in preventing rotavirus diarrhoea by number of doses received. Further exploratory analyses will include evaluation of Rotarix in preventing all-cause admission for diarrhoea. Additionally, because rotavirus diarrhoea will be detected from all study villages, we will be able to determine if children from the vaccine villages who are not vaccinated, because they were too old at the time the programme starts, have differing rates of rotavirus diarrhoea due to possible indirect effects of vaccination; such an approach has been used for other diseases. Finally, data from the intervention area villages will be linked to the dataset from the Rotateq trial to analyze the possible impact of previous use of a rotavirus vaccine in the study population on the evaluation of Rotarix.

Further details will be prespecified in a separate Statistical Analysis Plan document within six months of study initiation.

Ethical Assurance for Protection of Human Rights

Describe in the space provided the justifications for conducting this research in human subjects. If the study needs observations on sick individuals, provide sufficient reasons for using them. Indicate how subject's rights are protected and if there is any benefit or risk to each subject of the study.

Human rotavirus vaccine, Rotarix, designed for preventing rotavirus will be evaluated among young children since the risk of the disease is greatest in that age group. Infants will be enrolled after their parents have been given a full explanation of the study and they have understood the implications of participating in this study, and have agreed to participate in writing.

The principal investigator and his staff will take care of any possible immediate side effects of vaccination with Rotarix or any adverse events deemed by study investigators to be related to study participation. All treatment will be free of costs. No subjects will be deprived of existing care facilities.

Confidentiality of collected information will be maintained by keeping all data forms private and locked at the ICDDR,B Matlab office with access limited to those working in the study. Study subjects will only be identified by HDSS study numbers.

Use of Animals

Describe in the space provided the type and species of animal that will be used in the study. Justify with reasons the use of particular animal species in the experiment and the compliance of the animal ethical guidelines for conducting the proposed procedures.

No animal will be used in this study.

Literature Cited

Identify all cited references to published literature in the text by number in parentheses. List all cited references sequentially as they appear in the text. For unpublished references, provide complete information in the text and do not include them in the list of Literature Cited. There is no page limit for this section, however exercise judgment in assessing the "standard" length.

Anonymous. Withdrawal of rotavirus vaccine recommendation. JAMA 1999; 282:2113-4.

Bresee JS, Arifeen SE, Azim T, et al. Safety and immunogenicity of tetravalent rhesus-based rotavirus vaccine in Bangladesh. Pediatr Infect Dis J 2001; 20:1136-43.

Black RE, Huber DH, Curlin GT. Reduction of neonatal tetanus by mass immunization of non pregnant women:duration of protection provided by one or two doses of aluminium-adsorbed tetanus toxoid. Bull World Health Organ 1980; 58:927-30.

Blackwelder WC. Sample size and power for prospective analysis of relative risk. Statistics in Medicine 1993; 12:691-8.

Breiman RF, Streatfield PK, Phelan M, Shifa N, Rashid M, Yunus M. Effect of infant immunisation on childhood mortality in rural Bangladesh: analysis of health and demographic surveillance data.. Lancet 2004; 364:2204-11.

Chan ISF and Bohidar NR. Exact power and sample size for vaccine efficacy studies. Communications in Statistics: Theory and Methods 1998; 27:1305-22.

Eldridge SM, Ashby D and Kerry S. Sample size for cluster-randomized trials: effect of coefficient of variation of cluster size and analysis method. International Journal of Epidemiology 2006; 35:1291-1300.

Fauveau V, Koenig MA, Wojtyniak B, Chakraborty J. Impact of family planning and health services programme on adult female mortality. Health Pol Plann 1988; 3:271-9.

Fang ZY, Yang H, Qi J, et al. Diversity of rotavirus strains among children with acute diarrhoea in China: 1998-2000 surveillance study. J Clin Microbiol 2002; 40:1875-8.

Halloran ME, Struchiner CJ, Longini IM, Jr. Study designs for evaluating different efficacy and effectiveness aspects of vaccines. Am J Epidemiol. 1997;146(10):789–803.

International Centre for Diarrhoeal Disease Research Bangladesh (ICDDR,B). Health and Demographic Surveillance System-Matlab Vol. 37, Registration of Health and Demographic Events. *ICDDRB Sci Rep* 93; 2006.

Koenig MA, Roy NC, McElrath T, Shahidullah M, Wojtyniak B. Duration of protective immunity conferred by maternal tetanus toxoid immunization:further evidence from Matlab, Bangladesh. Am J Public Health 1998; 88:903-7.

Maneekarn N, Ushijima H. Epidemiology of rotavirus infection in Thailand. Pediatr Int 2000; 42:415-21.

Miller MA, McCann L. Policy analysis of the use of hepatitis B, *Haemophilus influenzae* type B, Streptococcus pneumonia-conjugate and rotavirus vaccines in National immunization schedule. Health Economics 2000; 9:19-35.

Murphy TV, Garguiullo PM, Masssoudi MS, et al. Intussusception among infants given an oral rotavirus vaccine. N Engl J Med 2001; 344:564-72.

Nguyen VM, Nguyen VT, Huynh PL, et al. The epidemiology and disease burden of rotavirus in Vietnam: sentinel surveillance at 6 hospitals. J Infect Dis 2001; 183:1707-12.

Nishio O, Matsui K, Lan DT, Ushijima H, Isomura S. Rotavirus infection among infants with diarrhea in Vietnam. Pediatr Int 2000; 42:422-4.

Punyaratabandhu P, Vathanophas K, Varavithya W, et al. Childhood diarrhoea in a low-income urban community in Bangkok: incidence, clinical features, and child caretaker's behaviours. J Diarrhoeal Dis Res 1991; 9:244-9.

Rennels MB, Parashar UD, Holman RC, Le CT, Chang H-G, Glass RI. Lack of an apparent association between intussusception and wild or vaccine rotavirus infection. Pediatr Infect Dis J 1998; 17:924-5.

Ruiz-Palacios GuillermoM. et al. Safety and efficacy of an attenuated vaccine against severe rotavirus gastroenteritis. N Engl J Med 2006; 354:11-22.

Ruuska T and Vesikari T. Rotavirus disease in Finnish children:use of numerical scores for severity of diarrheal episodes. Scand J Infect Dis 1990; 22:259-67.

Seo JK, Sim JG. Overview of rotavirus infections in Korea. Pediatrics International 2000; 42:406-10.

Tsai CH, Chiu HH, Abe T. Epidemiologic features of rotavirus infection in Taiwan:a review. Pediatrics International 2000; 42:411-14.

Unicomb et al. Evidence of high-frequency genomic reassortment of group A rotavirus strains in Bangladesh:emergence of type G9 in 1995. J Clin Microl 1999; 37:1885-91.

Vesikari T, Karvonen A, Prymula R, et al. Efficacy of human rotavirus vaccine against rotavirus gastroenteritis during the first 2 years of life in European infants: randomised, double-blind controlled study. Lancet 2007; 370:1752-69.

World Health Organization. 1987. Programme for control of diarrhoeal disease (CDD/93.3 Rev 1), p. 9-20. In Manual for laboratory investigation of acute enteric infections. World Health Organization, Geneva, Switzerland.

World Health Organization “Estimated rotavirus deaths for children under 5 years of age:2004, 527 000” http://www.who.int/immunization_monitoring/burden/rotavirus_estimates/en/index.html WHO Website, 02 Jan 2007a.

World Health Organization. Global Advisory Committee on Vaccine Safety, 12–13 June 2007. Weekly Epidemiological Record 2007b; 82:252-9.

Yap KL, Yasmin AM, Wong YH, et al. A one year community-based study on the incidence of diarrhoea and rotavirus infection in urban and suburban Malaysian children. Med J Malaysia 1992; 47:303-8.

Zaman K, Sack DA et al. Immunogenicity and reactogenicity of a human rotavirus vaccine in Bangladesh children. Abstract:12th Asia Pacific Congress of Pediatrics, Colombo, SriLanka, 2007. Also in abstract:4th International Conference on Vaccine for Enteric Diseases (VED), Lisbon:2007.

Dissemination and Use of Findings

Describe explicitly the plans for disseminating the accomplished results. Describe what type of publication is anticipated: working papers, internal (institutional) publication, international publications, international conferences and agencies, workshops etc. Mention if the project is linked to the Government of Bangladesh through a training programme.

The findings from this study will be published in internal publications and peer reviewed journals, and disseminated in national and international conferences.

Collaborative Arrangements

Describe briefly if this study involves any scientific, administrative, fiscal, or programmatic arrangements with other national or international organizations or individuals. Indicate the nature and extent of collaboration and include a letter of agreement between the applicant or his/her organization and the collaborating organization. **(DO NOT EXCEED ONE PAGE)**

We will collaboration with National EPI Programme of Bangladesh. More specifically, we will work with EPI managers and staff at the government's health complexes in Matlab Upazila of Chandpur District and in Daudkandi Upazila of Comilla District, the two Upazilas covering the Matlab HDSS.

Dr. David Sack, former Executive Director of the ICDDR,B who was instrumental in planning for the trial, is now a professor at Johns Hopkins University and will be included as an investigator on the trial. He has continued to be involved with the trial by electronic communication, and will visit Bangladesh as needed through the trial.

We will also collaborate with the Rotavirus Vaccine Program (a PATH) in scientific and ethical review for this study and in analyses of data. This collaboration will be detailed in a formal contract between ICDDR,B and PATH.

GSK will provide all the vaccines for the study free of costs. Estimated annual doses of vaccine for the purpose of the study are as follows: 2008, 170 doses; 2009, 4280 doses; 2010, 5042 doses; 2011, 2650 doses.

Biography of the Investigators

Give biographical data in the following table for key personnel including the Principal Investigator. Use a photocopy of this page for each investigator.

(Note: Biography of the external Investigators may, however, be submitted in the format as convenient to them)

1 **Name:** K. Zaman

2 **Present Position:** Scientist and Epidemiologist

3 **Educational background:**
(last degree and diploma & training relevant to the present research proposal)

PhD- Johns Hopkins Bloomberg School of Public Health, USA
MPH- Johns Hopkins Bloomberg School of Public Health, USA
MBBS- Rajshahi Medical College, Bangladesh

4.0 List of ongoing research protocols
(start and end dates; and percentage of time)

4.1. As Principal Investigator

Protocol Number	Starting date	End date	Percentage of time
2003-042	June 2004	Dec 2006	10
2006-44	January 2007	Jan 2009	50
2005-035	March 2006	Feb 2007	20
2006-011	July 2006	June 2009	10

4.2. As Co-Investigator

Protocol Number	Starting date	End date	Percentage of time

5 Publications

Types of publications	Numbers
a. Original scientific papers in peer-review journals	48
b. Peer reviewed articles and book chapters	
c. Papers in conference proceedings	67
d. Letters, editorials, annotations, and abstracts in peer-reviewed journals	4
e. Working papers	
f. Monographs	

6 Five recent publications including publications relevant to the present research protocol

1) **Zaman K**, Takeuchi H, Yunus M, Arifeen SE, Chowdhury HR, Baqui AH, Wakai S, Iwata T. Prevalence and risk factors for wheezing among rural Bangladeshi children: an epidemiological study. **Ind J Pediatrics 2007; 74: 539-543.**

2) **Zaman K**, Yunus M, Arifeen SE, Baqui AH, Sack DA, Hossain S, Rahim Z, Ali M, Banu S, Islam MA, Begum N, Begum V, Breiman RF, Black RE. Prevalence of sputum smear positive tuberculosis in a rural area in Bangladesh. **Epidemiology and Infection 2006; 29:1-8.**

3) **Zaman K**, Rahim Z, Yunus M, Baqui AH, Srifeen SE, Sack DA, Hossain Shahed, Banu S, Islam Md Akramul, Ahmed Jalaluddin, Breiman RF, Black RE. Drug resistance of Mycobacterium tuberculosis in rural and urban areas in Bangladesh. **Scand J Infect Dis 2005; 37: 21-26**

4) **Zaman K,** Sack DA, Chakraborty JC, Yunus M, Baqui AH, Black RE. Children's fluid intake during diarrhea: a comparison of questionnaire responses with data from observations. **Acta Paediatrica 2002; 91: 376-382**.

5) **Zaman K**, Yunus M, Rahman A, Chowdhury HR, Sack DA. Efficacy of a packaged rice ORS among children with cholera and cholera like illness. **Acta Paediatrica 2001; 90; 505-510.**

6) **Zaman K**, Baqui AH, Yunus M, Sack RB, Bateman OM, Chowdhury HR, Black RE. Acute respiratory infections in children: a community based longitudinal study in rural Bangladesh. **J Trop Pediatrics 1997; 43:133-137**.

Detailed Budget

Budget Justifications

The total duration of the proposed study will be of 36 months. The study involves recruitment and training of staffs, approval from GoB, identification of all births during a year from the selected villages, vaccination to all eligible infants with rotavirus vaccine, passive surveillance of diarrhoea cases and collection of stool specimens and test of sample for RV.

Investigators:

The amount budgeted for the investigators reflects a reasonable estimate of the minimum time required to implement the study. Dr. K. Zaman, PI of the study will be responsible for overall implementation of the study. Dr. Md Yunus will provide support for overall implementation as well as advice on epidemiological aspects. Dr. Shams El Arifeen will provide advice on epidemiological aspects to conduct the study. Dr. ASG Faruque on clinical aspects while Drs. Tasnim Azim and Goutam Podder will be responsible for laboratory methods. Prof. David Sack and Dr. Steve Luby will provide overall guidance of the study. The salaries of the investigators have been partly covered by from the another ongoing rotavirus vaccine study (protocol # 2006-044).

Research Officer : For processing and testing of samples.

Field workers: Assist GoB EPI personnel in giving vaccines.

Supplies and equipment

Cold box: Keeping and transportation of specimens.

Laboratory tests: ELISA for identification of rotavirus and G and P types and isolation of enteric pathogens.

Transport costs: Transport cost for field work, supervise data collection, visit families, bringing of subjects and parents for vaccination, and cost for travel between Dhaka and Matlab.

Appendix I

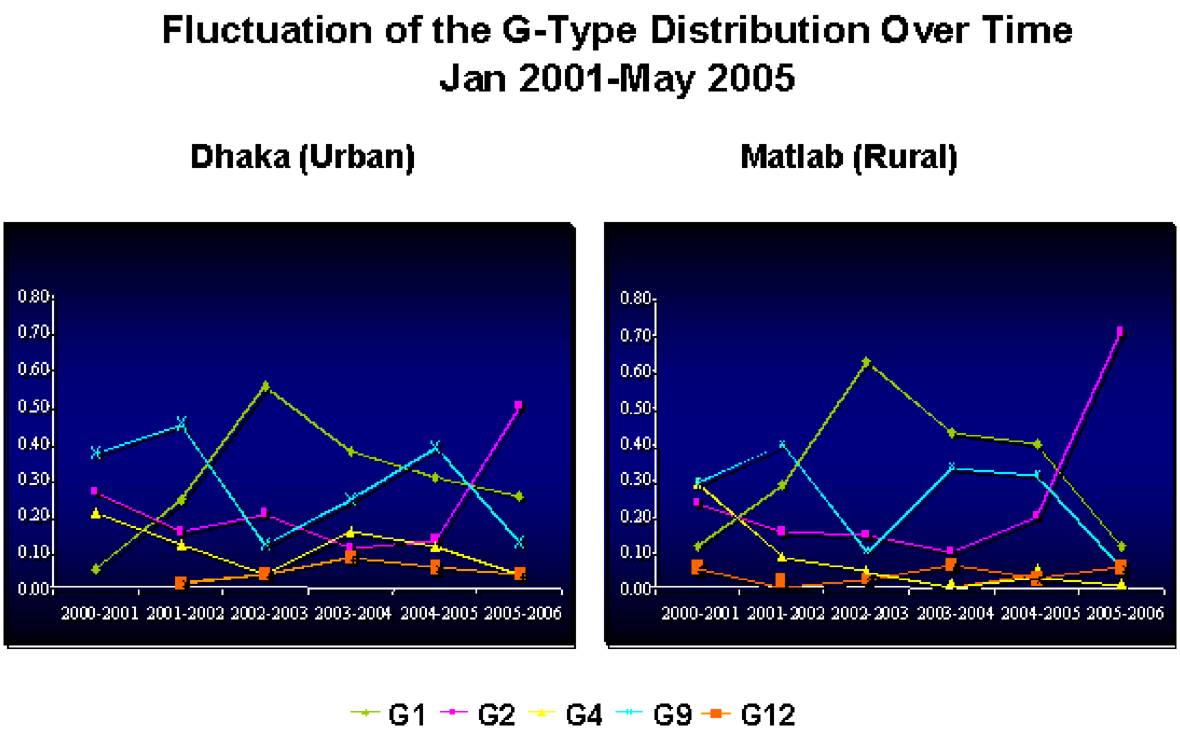
Fluctuation of the G-Type Distribution Over Time
Jan 2001-May 2005
Dhaka (Urban)
Matlab (Rural)
0.80
0.70
0.60
0.50
0.40
0.30
0.20
0.10
0.00
2000-2001 2001-2002 2002-2003 2003-2004 2004-2005 2005-2006
G1 G2 G4 G9 G12

Appendix II

Clinical scoring system

The completion of the diary card for each GE episode will allow the assessment of the intensity by using a 20-point scale. In this system, points will be assigned according to duration and intensity of diarrhoea and vomiting, the intensity of fever, presence of dehydration or hospitalisation for each episode of GE as shown below:

Sign/Symptom		Points
**Duration of looser than normal stools (days)**		
1-4		1
5		2
≥ 6		3
**Maximum number of looser than normal stools /24 hours**		
1-3		1
4-5		2
≥ 6		3
**Duration of vomiting (days)**		
1		1
2		2
≥ 3		3
**Maximum number of episodes of vomiting/24 hours**		
1		1
2-4		2
≥ 5		3
**Fever***		
**Rectally**	**Axillary**	
37.1 – 38.4°C	36.6 – 37.9°C	1
38.5 – 38.9°C	38.0 – 38.4°C	2
≥ 39°C	≥ 38.5°C	3
**Dehydration****		
1-5%		2
≥ 6%		3
**Treatment**		
Rehydration		1
Hospitalisation		2

∗ The highest temperature recorded during the episode will be scored.

** Dehydration wiill be assessed clinically based on World Health Organisation criteria.
