# Supplementary material for: Effectiveness of a live oral human rotavirus vaccine after programmatic introduction in Bangladesh: A cluster-randomized trial
Source: PLoS Med. 2017 Apr 18;14(4):e1002282. doi: 10.1371/journal.pmed.1002282 (PMC5395158; doi:10.1371/journal.pmed.1002282)
Supplement: S2 Text — (PDF) [file pmed.1002282.s002.pdf]

WIRB®

(360) 252-2500
1-800-562-4789
FAX: (360) 252-2498

Western Institutional Review Board ®

Western International Review Board®

3535 SEVENTH AVENUE, SW, OLYMPIA, WA 98502-5010
P.O. BOX 12029, OLYMPIA, WA 98508-2029

Certificate of Approval

THE FOLLOWING WERE APPROVED:

INVESTIGATOR: K. Zaman MPH, Ph.D.
Child Health Unit, Public Health Sciences Division
ICDDR,B, GPO Box 128
Dhaka, 1000
Bangladesh

BOARD ACTION DATE: 4/30/2008
PANEL: 11
STUDY APPROVAL EXPIRES: 4/30/2009
STUDY NUM: 1098600
WIRB PRO NUM: 20080624
INVEST NUM: 127980
WO NUM: 1-485811-1
CONTINUING REVIEW: Annually
SITE STATUS REPORTING: Annually

SPONSOR: Program for Appropriate Technology in Health (PATH)
PROTOCOL NUM: 2007-024
AMD. PRO. NUM:
TITLE:
Introduction of an oral live human rotavirus (Rotarix) vaccine in Matlab

APPROVAL INCLUDES:

Investigator
Protocol (03-31-2008)
APPROVED Consent Form - Illness
APPROVED Consent Form - Vaccination

WIRB APPROVAL IS GRANTED SUBJECT TO:

ASSENT DETERMINATION: No Assent Required

IF YOU HAVE ANY QUESTIONS, CONTACT WIRB AT 1-800-562-4789
This is to certify that the information contained herein is true and correct as reflected in the records of the Western Institutional Review Board (WIRB). WE CERTIFY THAT WIRB IS IN FULL COMPLIANCE WITH GOOD CLINICAL PRACTICES AS DEFINED UNDER THE U.S. FOOD AND DRUG ADMINISTRATION (FDA) REGULATIONS AND THE INTERNATIONAL CONFERENCE ON HARMONISATION (ICH) GUIDELINES.

Theodore D. Schultz, J.D., Chairman

5/6/2008
(Date)

This document electronically reviewed and approved by Orive, Otto on 5/6/2008 8:19:57 AM PST. For more information call Client Services at 1-360-252-2500

Copyright © 2008 Western Institutional Review Board, Inc. All rights reserved.

WIRB HAS APPROVED THE FOLLOWING LOCATIONS TO BE USED IN THE RESEARCH:

- ICDDR,B's Matlab Health and Demographic Surveillance Survey (HDSS) area, Bangladesh, Matlab Health Research Centre, PO Matlab, Dist-Chandppur, , Bangladesh

If the PI has an obligation to use another IRB for any site listed above and has not submitted a written statement from the other IRB acknowledging WIRB's review of this research, please contact WIRB's Client Services department.

ALL WIRB APPROVED INVESTIGATORS MUST COMPLY WITH THE FOLLOWING:

1. Conduct the research in accordance with the protocol, applicable laws and regulations, and the principles of research ethics as set forth in the Belmont Report.

2. Unless consent has been waived, conduct the informed consent process without coercion or undue influence, and provide the potential subject sufficient opportunity to consider whether or not to participate.

    a. Use only the most current consent form bearing the WIRB "APPROVED" stamp.

    b. Provide non-English speaking subjects with a certified translation of the approved consent form in the subject's first language. The translation must be approved by WIRB.

    c. Obtain pre-approval from WIRB for use of recruitment materials and other materials provided to subjects.

3. Obtain pre-approval from WIRB for any planned deviations that could adversely affect the safety or welfare of subjects, or the integrity of the research data and any changes in the research activity. The only exception is when changes are necessary to eliminate apparent immediate hazards to subjects. Immediately report to WIRB any such emergency changes implemented.

4. Promptly report to WIRB any new information that may adversely affect the safety of the subjects or the conduct of the trial.

    a. Report to WIRB all adverse events that are unanticipated and possibly related, within 10 days of the investigator becoming aware of them.

    b. Promptly report to WIRB other unanticipated problems involving risks to human subjects or others. These events do not readily fit the formal definition of Adverse Event, but could impact human subject safety and/or rights. Examples include theft of a computer containing private identifiable subject information, or study staff getting ill from inhaling a study agent.

    c. Provide reports to WIRB concerning the progress of the research, when requested.

5. Report to WIRB any unplanned protocol variance that could adversely affect the safety or welfare of subjects, or the integrity of the research data, within 10 days of becoming aware of the variance. Other unplanned variances may be recorded on a log and submitted with continuing review reports.

Federal regulations require that WIRB conduct continuing review of approved research. You will receive Continuing Review Report forms from WIRB. These reports must be returned even though your study may not have started.

DISTRIBUTION OF COPIES:

Contact	Company Name
K. Zaman MPH, Ph.D.	International Centre for Diarrhoeal Disease Research (Bangladesh)
Elizabeth Trias MA	Program for Appropriate Technology in Health (PATH)
John C. Victor Ph.D.	Program for Appropriate Technology in Health (PATH)

Copyright © 2008 Western Institutional Review Board, Inc. All rights reserved.
