# Supplementary material for: Effectiveness of a live oral human rotavirus vaccine after programmatic introduction in Bangladesh: A cluster-randomized trial
Source: PLoS Med. 2017 Apr 18;14(4):e1002282. doi: 10.1371/journal.pmed.1002282 (PMC5395158; doi:10.1371/journal.pmed.1002282)
Supplement: S3 Text — (PDF) [file pmed.1002282.s003.pdf]

icddr,b
KNOWLEDGE FOR
GLOBAL LIFESAVING SOLUTIONS

Memorandum

12 June 2007

To : Dr. K. Zaman
Principal Investigators of research protocol # 2007-024
Public Health Sciences Division (PHSD)

From: Professor AKM Nurul Anwar
Chairman
Ethical Review Committee (ERC)

Sub : Approval of research protocol # 2007-024

Thank you for your memo dated 06 June 2007 attaching the modified version of your research protocol # 2007-024 titled "**Introduction of an oral live human rotavirus (RotaRix) vaccine in Matlab**". The issues that were raised by the ERC in its meeting held on 30th May 2007 on your research protocol have been addressed in the modified version of the research protocol to the satisfaction of the Committee. Accordingly, the Committee approved the research protocol. You will be required to observe the following terms and conditions in implementing the research protocol:

1. As Principal Investigator, the ultimate responsibility for scientific, and ethical conduct including the protection of the rights and welfare of study participants vest upon you. You shall also be responsible for ensuring competence, integrity and ethical conduct of other investigators and staff directly involved in this research protocol.

2. You shall conduct the study in accordance with the ERC-approved protocol and shall fully comply with any subsequent determinations by the ERC.

3. You shall obtain prior approval from the Research Review Committee and the ERC for any modification in the approved research protocol and/or approved consent form(s), except in case of emergency to safeguard/eliminate apparent immediate hazards to study participants. Such changes must immediately be reported to the ERC Chairman.

4. You shall recruit/enroll participants for this study strictly adhering to the criteria mentioned in the research protocol.

5. You shall obtain legally effective informed consent (i.e. consent should be free from coercion or undue influence) from the selected study participants or their legally responsible representative, as approved in the protocol, using the approved consent form prior to their enrollment in this study. Before obtaining consent, all prospective study participants must be adequately informed about the purpose(s) of the study, its methods and procedures, and also what would be done if they agree and also if they do not agree to participate in the study. They must be informed that their participation in the study is voluntary and that they can withdraw their participation any time without any prejudice. Signed consent forms should be preserved for a period of at least five years following official termination of the study.

International Centre for Diarrhoeal Disease Research Bangladesh
68 Shaheed Tajuddin Ahamed Sharani, Mohakhali, Dhaka 1212, Bangladesh
Mail : ICDDR,B, GPO Box 128, Dhaka 1000, Bangladesh
Fax : 880-2-8823116, 8812530, 9885657, 8811686, 8812529, 8826050, 8811568

6. You shall promptly report the occurrence of any Adverse Event or Serious Adverse Event or unanticipated problems of potential risk to study participants or others to the ERC in writing within 24 hours of such occurrences.

7. Any significant new findings, developing during the course of this study that might affect the risks and benefits and thus influence either participation in the study or continuation of participation should be reported in writing to the participants and the ERC.

8. Data and/or samples should be collected and interviews should be conducted, as specified in the ERC-approved protocol, and confidentiality must be maintained. Data/samples must be protected by reasonable security, safeguarding against risks such as their loss or unauthorized access, destructions, used by others, and modification or disclosure of data. Data/samples should not be disclosed, made available to or use for purposes other than those specified in the protocol, and shall be preserved for a period, as specified under Centre's policies/practices.

9. You shall promptly and fully comply with the decision of the ERC to suspend or withdraw its approval for the research protocol.

10. You shall report progress of research to the ERC for continuing review of the implementation of the research protocol as stipulated in the ERC Guidelines. Relevant excerpt of ERC Guidelines and '*Annual/Completion Report for Research Protocol involving Human Subjects*' are attached for your information and guidance.

I wish you success in running the above-mentioned study.

Copy: Director, PHSD
