# Supplementary material for: Effectiveness of a live oral human rotavirus vaccine after programmatic introduction in Bangladesh: A cluster-randomized trial
Source: PLoS Med. 2017 Apr 18;14(4):e1002282. doi: 10.1371/journal.pmed.1002282 (PMC5395158; doi:10.1371/journal.pmed.1002282)
Supplement: S4 Text — (PDF) [file pmed.1002282.s004.pdf]

STATISTICAL ANALYSIS PLAN

Introduction of an oral live human rotavirus (ROTARIX®) vaccine in Matlab

TABLE OF CONTENTS

1 INTRODUCTION 3

2 BACKGROUND 3
- 2.1 Study Overview 3
- 2.2 Randomization 4
- 2.3 Patient Evaluations 5
- 2.4 Data Capture 5
- 2.5 Sample Size Considerations 6

3 STATISTICAL METHODS 7
- 3.1 General Considerations 7
- 3.2 Analysis Populations 8
- 3.3 Baseline Characteristics 9
- 3.4 Vaccine Exposure 9
- 3.5 Case Definitions and Severity Score 10
- 3.6 Statistical Analyses 12
- 3.7 Exploratory Analyses 15

4 INTERIM ANALYSIS 16

1 INTRODUCTION

The main purpose of this document is to describe the planned statistical analyses of this study so as to avoid “data dredging” at the time of preparing analyses to inform policy and for publication. Some background information and sample size calculations are copied here from the protocol dated 21.02.11 for ease of reference.

This document will guide analyses and may be given to journal editors for clarification of analyses and their timing. This document is being finalized months before the end of data collection, without any knowledge of treatment arm-specific results, and only a general idea of enrollment and total numbers of events.

2 BACKGROUND

A safe and effective vaccine is needed to reduce the enormous public health burden associated with rotavirus illness, especially in developing countries. About 40,000 children with rotavirus diarrhoea are treated each year at the ICDDR,B hospitals. Globally, there are an estimated 527,000 deaths annually. Unanticipated adverse events (intussusception) experienced with a rhesus rotavirus vaccine have accelerated efforts to develop and evaluate alternative vaccine candidates so that a safe and effective public health tool would become available. GSK Biologicals’ live attenuated human rotavirus vaccine (HRV), Rotarix has been tested in clinical studies and was shown to be efficacious, safe and well tolerated among infants. The vaccine has been licensed in more than 80 countries, including Bangladesh.

2.1 Study Overview

The study will be conducted in the Matlab Health and Demographic Surveillance System (HDSS) field area of the International Centre for Diarrhoeal Disease Research, Bangladesh (ICDDR,B) to determine the population effectiveness of Rotarix in Bangladeshi children. Villages in both the Maternal and Child Health and Family Planning intervention area (hereafter referred to as the ICDDR,B service area) and the comparator Government service area will be included in this evaluation. We propose to introduce Rotarix into half of the villages of the Matlab HDSS. In villages randomized to receive the vaccine, all eligible children will be offered Rotarix during their first two Expanded Programme on Immunization (EPI) visits, as would routinely be done if Rotarix were included in the Bangladesh EPI schedule. If a child residing in a village assigned to Rotarix vaccine did not receive Rotarix during the first EPI visit, the child may still receive the Rotarix during subsequent EPI visits so long as the vaccine is given within age guidelines as defined by the Rotarix package insert. In villages randomized not to receive Rotarix, children will receive their EPI vaccinations exactly as they would have in the absence of this study. Administration of Rotarix will be conducted by regular EPI staff, but ICDDR,B study staff will be present to document informed consent and collect study-specific information. The Ministry of Health will be an active partner in this evaluation since they will be the agency which may follow up with any subsequent vaccine programme. Vaccination with Rotarix will be

recorded on the infant's immunization card which is normally used by the EPI programme, but also on a separate study-specific data collection form.

Rotarix vaccination started in the government service area villages in September 2008 (although was not fully operational until November 1 of that year) and in the ICDDR,B serivce area villages on April 1, 2009. The delayed start in the ICDDR,B service area was to allow for completion of follow-up in the RotaTeq individually-randomized trial which was carried out in the 67 villages of the ICDDR,B service area.

Vaccination with Rotarix and surveillance activities for trial purposes will continue from study initiation through March 31, 2011. Surveillance for rotavirus gastroenteritis among children less than 2 years of age will occur at Matlab Diarrhoeal Hospital and the two community treatment centres of the Matlab HDSS continuously throughout the study period. Diarrheal illness information collected through surveillance will be linked to Rotarix study-specific data using the subject's HDSS identification numbers. The primary study endpoint will be the occurrence of an illness episode of acute diarrhoea, among infants and children presenting to a diarrhea treatment centre, determined to be caused by wild-type rotavirus found in a stool specimen. At the end of the surveillance period, rates of this primary study endpoint among age-eligible infants will be compared for villages randomized to receive Rotarix versus for villages randomized not to receive Rotarix. We expect that the rates of rotavirus diarrhoea will be significantly lower among children from the vaccinated villages. "Age-eligible" children is the cohort of children whose birthdate indicates that they could have received rotavirus vaccine if they resided in the villages assigned to vaccine, regardless of whether they actually received it (e.g. an intention to treat analysis comparing rates of rotavirus diarrhea among the cohort of children who were eligible to receive vaccine compared to age eligible children in the comparison villages who did not receive vaccine.)

2.2 Randomization and Blinding

There are 75 villages in the government comparison area and 67 villages in the intervention area. The villages in each area will be randomized separately. Infants in half of the villages in each area will be eligible to receive Rotarix during their EPI doses. In each area, villages will be randomly selected for Rotarix introduction after pre-stratifying on village size and baseline hospitalization rates/numbers for diarrhoea at the Matlab Diarrhoeal Hospital and community treatment centres during 2005 and 2006. Hospital charts for diarrhoea admissions will be reviewed to determine the number/rates of hospitalizations from those villages. The villages will be arranged sequentially based on diarrhoea admission number/rates to the Matlab and Nayergaon and Kalirbazaar treatment centres. For each stratum of villages, half of the villages will be randomly selected for introduction of Rotarix and the other half will for no introduction of Rotarix.

Although not a blinded evaluation, the introduction of bias will be reduced in several ways. First, the vaccine teams located in the EPI centres that are completely separate from the clinical settings where clinicians will be conducting surveillance. Second, the clinicians who treat the children when they present to a treatment facility are separated from those staff who carry out the vaccinations or conduct the laboratory testing. Third, the illness episodes are widely separated in

time from the vaccination, and thus the illness episode is not easily associated with receipt of vaccine. Fourth, laboratory assays for rotavirus will be carried out in the laboratory in Dhaka using coded specimens and are not conducted at the time of illness (since they have little clinical value). Although a parent could inform the clinician as to his/her child's participation in the study, this is believed to be an unlikely or important source of bias.

2.3 Patient Evaluations

Study personnel will record the child's symptoms and clinical history, and collect a fecal specimen for laboratory testing for rotavirus. The identity of the child will be confirmed through the HDSS subject identification number system. All patients coming to the Matlab Diarrhoea Hospital and Nayergoan community treatment centre from these villages are already included in the Matlab diarrhoea surveillance system, and faecal samples are routinely tested for rotavirus, vibrios, salmonellae, shigellae, and enterotoxigenic E coli, with results linked to demographic and health databases of the HDSS. Kalirbazaar treatment centre is not currently included in the current surveillance system, but we will establish surveillance at this centre during this study. All children from the Matlab HDSS who present with acute gastroenteritis have stool specimens collected for testing for rotavirus by EIA. Additionally, all children have illness information recorded on a standard format Diarrhoea Treatment Record which contains information on length of illness, dehydration status, associated symptoms, outcome of illness and lab results. [This record contains sufficient clinical data to calculate a "Vesikari score" for most illnesses (Ruuska and Vesikari, 1990).Symptom data was collected using an illness form with some data abstracted from the patient record (See GE Guidelines document, attached).

Although most cases are discharged after complete recovery of diarrhoeal illnesses, there may be few cases in which the episode has not resolved fully at the time of discharge. In order to treat subjects from vaccinated and unvaccinated villages equivalently, there will be no home visits to obtain final outcomes in order to have all necessary information to score their illness according to the Vesikari score.

Enrollment form_V21Jul09.pdf   patient record.pdf   GE Guidelines v1 25Aug08_v1.doc   Illness Rotarix v1 25Aug08_v1.doc

2.4 Data Capture

As is currently being done as part of disease surveillance in the hospital, when a child is admitted for acute gastroenteritis, the child is identified by his/her HDSS number and link the child to his/her village of residency. In all cases this can be accomplished.

2.5 Sample Size Considerations

2.5.1 Sample size calculations

The primary study endpoint will be admission for rotavirus gastroenteritis (see definitions, section 2.3.8). From 2003 to 2006 the average annual rotavirus admission rate to the Matlab Diarrhoeal Hospital among infants 0 to 11 months of age from the government comparison area was about 18.5 per 1000 and among infants 12 to 23 months of age it was 12.3 per 1000 (Table 3a); among infants 0 to 11 months of age from the intervention area it was 37.3 per 1000 and among infants 12 to 23 months of age it was 24.5 per 1000 (Table 3b). Because we will conduct surveillance among age-eligible infants continuously from study initiation, infants enrolled early will fall under longer surveillance while infants enrolled later will fall under brief surveillance (for example, only a few months). Additionally, because enrolment and surveillance for rotavirus diarrhoea will occur in the government comparison area and intervention area along different timelines, we used modeling to estimate a total study-period rotavirus diarrhoea hospitalization rate of 33 per 1000 infants (3.3%) (data not shown). With inclusion of infants admitted for rotavirus gastroenteritis at the Nayergaon and Kalirbazaar Community Treatment Centres, this rate may be slightly higher at 3.5%.

Because this is a cluster-randomized design, we must account for the intracluster (intra-village) correlation of acute rotavirus gastroenteritis. At the time of study planning, this value is not available for the study population. However, given the ubiquitous nature of rotavirus, we expected this value to be low (we assumed 0.02). Additionally, because we predefine clusters as villages for this study, we cannot set the cluster sizes to be equal; villages from the government comparison and intervention areas vary substantially in size (numbers of annual births). According to the timelines for enrolment for the 142 villages in from each area, the average cluster size is expected to be 65 with a coefficient of variation of cluster sizes is 0.96. Factoring in these values, along with the assumed intracluster correlation coefficient, into the calculation of the design effect for a cluster-randomized trial (Eldridge, 2006), we calculate a design effect of 3.48. Later estimations of the design effect based on rotavirus admission data for Matlab hospital place the design effect in the range of 2.9 to 4.5.

We calculated sample size and power based upon a fixed number of events design and conditional on the total number of cases observed (Blackwelder, 1993; Chan 1998). The number of cases in each group was assumed to follow a Poisson distribution, with parameters λ_V for a vaccine villages and λ_U for the unvaccinated villages. Under this assumption, the number of vaccine cases, x_V, has a binomial distribution (X, P) conditional on X, the total number of cases, with $P = \lambda_V/(\lambda_V+\lambda_U)$. For the primary objective, we assumed 50% vaccine population (overall) effectiveness. With no design effect (DE=1), observation of at least 77 admissions for rotavirus gastroenteritis among all age-eligible infants from vaccine and unvaccinated villages would ensure that the study has a minimum power of 80% to rule out a lower bound of 0% overall effectiveness. However, with a design effect of 3.48, a minimum of 268 admissions for rotavirus diarrhoea must be observed. Assuming a 3.5% total study-period attack rate in the unvaccinated villages, 268 admissions translates into an estimated minimum sample size of 10,210 infants (5105 in each group, vaccinated villages and unvaccinated villages) (Table 6).

Sample size for the immunogenicity subset

We assume that the seropositive rate of the immunized children with Rotarix would be 60% and for the non immunized children be 35%. A sample size of 90 in each group would allow

detection of this difference with an alpha of 0.05, two-sided, and 90% power. Assuming a 10% loss to follow up, 100 subjects in each group are needed to meet the required sample size

Table 6: Total sample size estimates to detect a reduction in population incidence of rotavirus diarrhoea hospitalizations for different population effectiveness and baseline incidence rate assumptions, different design effect (DE) values and different numbers of required endpoints (type II error of 5%, 2-sided and 80% power).

**DE=3.48**	**Vaccine Effectiveness**			
	**50%**	**60%**	**70%**	**80%**
**Incidence***	**Required number of outcomes**			
	**268 cases**	**164 cases**	**104 cases**	**59 cases**
4.00	8937	5846	4016	2464
3.75	9528	6229	4287	2631
**3.50**	**10210**	6675	4594	2819
3.25	10997	7190	4942	3035
3.00	11909	7788	5352	3285

*RV admission rate per 100 children
(Assumes 1:1 randomization. Half of these totals sample sizes may receive Rotarix.)

During the planned course of the study (through March 2011), it is estimated that a total of approximately 11,650 infants will be born into both groups (5825 per group, section 2.3.3). While this estimated total study population is higher than that required to achieve 80% power (10,210), if population effectiveness or the underlying attack rate of were lower than 50% or 3.5%, respectively, the study would quickly become underpowered. Because of these uncertainties we plan to continue enrollment and follow-up through March 2011 (which is practically to the end of the “winter” rotavirus season) in order to assure the study will have sufficient power.

3 STATISTICAL METHODS

3.1 General Considerations

Data analyses will be conducted using Stata Version 11 (StataCorp, College Station, Texas) and SAS® System Version 9.2 (SAS Institute Inc., Cary, NC) in a Microsoft® Windows® environment.

3.2 Analysis Populations

Exclusions:
Except for the Total population, there will be no late exclusions from the analyses based on the study’s inclusion/exclusion criteria for immunization. This is to preserve the effectiveness goals of the study.
In the rest of this SAP, “November 1, 2008” and “April 1, 2009” will be used as shorthand for the dates of first study administration of Rotarix in the government service area and ICDDR,B service area, respectively.

Regardless of the analysis, infants are assigned as their village of residence the village in which they were residing when they turned 6 weeks or age, or when they in-migrated (if they in-migrated at an age greater than 6 weeks).

The following participant populations will be used for analyses:

<table>
<tr><th colspan="2">“Overall effectiveness” (primary objective) analysis populations</th></tr>
<tr><td></td><td>Primary analysis
<u>Those included:</u> Infants who are residents in a Matlab village on the date upon which they turn 6 weeks (42 days) of age between Apr 1, 2009 (for ICDDR,B service area villages) or Nov 1, 2008 (for government service area villages) and March 31, 2011. In-migrant infants will be included if they in-migrate to a Matlab village when they are between 6 weeks and 20 weeks of age between Apr 1, 2009 (ICDDR,B service area) or Nov 1, 2008 (Govt service area) and Mar 31, 2011.

<u>Follow-up period:</u> Person-time will be counted from 6 weeks of age or age of in-migration until two years (731 days) of age or March 31, 2011, whichever comes first.

Secondary analysis
<u>Those included:</u> Identical to those in the “overall effectiveness-primary analysis” population plus those infants residing in study area villages who are between 6 and 20 weeks of age <u>on</u> Apr 1, 2009 (ICDDR,B service area) or <u>on</u> Nov 1, 2008 (Govt service area). Inclusion will then be restricted to those who received OPV1 (exclusive of birth dose) in a Matlab village after Apr 1, 2009 (ICDDR,B service area) or Nov 1, 2008 (Govt service area).

<u>Follow-up period:</u> Person-time will be counted from the date of OPV1 (exclusive of birthdose) until two years (731 days) of age or March 31, 2011, whichever comes first.</td></tr>
<tr><th colspan="2">“Total effectiveness” populations</th></tr>
<tr><td>Modified Intent-to-treat (mITT) populations for total effectiveness</td><td>First priority analysis
<u>Those included:</u> Infants in the “overall effectiveness-secondary analysis” population who 1) received at least one dose of Rotarix and received OPV1 (exclusive of birthdose) while residing in a Rotarix village between 6 and 20 weeks of age after Apr 1, 2009 (ICDDR,B service area) or after Nov 1, 2008 (Govt service area) or 2) received OPV1 (exclusive of birthdose) while residing in a non-Rotarix village between 6 and 20 weeks of age after Apr 1, 2009 (ICDDR,B service area) or after Nov 1, 2008 (Govt service area).</td></tr>
</table>

	Follow-up period: Person-time will be counted from the date of OPV1 (exclusive of birth dose) until two years (731 days) of age or March 31, 2011, whichever comes first.<br>**Second priority analysis**<br>Those included: Infants in the "**overall effectiveness-primary analysis**" population who 1) received at least one dose of Rotarix while residing in a Rotarix village between 6 and 20 weeks of age after Apr 1, 2009 (ICDDR,B service area) or after Nov 1, 2008 (Govt service area) or 2) resided in a non-Rotarix village between 6 and 20 weeks of age after Apr 1, 2009 (ICDDR,B service area) or Nov 1, 2008 (Govt service area).<br><br>Follow-up period: Person-time will be counted from 6 weeks of age or age of in-migration until two years (731 days) of age or March 31, 2011, whichever comes first.
According-to-Protocol (ATP) populations for total effectiveness	**First priority analysis**<br>Those included: Infants in the "**mITT total effectiveness first priority**" population who complete both the second dose of Rotarix and OPV2 (exclusive of birthdose) in Rotarix villages and OPV2 (exclusive of birthdose) in non-Rotarix villages.<br><br>Follow-up period: Person-time will be counted from date of OPV2 (exclusive of birth dose) plus 2 weeks until two years (731 days) of age or March 31, 2011, whichever comes first.<br><br>**Second priority analysis**<br>Those included: Infants in the "**mITT total effectiveness second priority**" population who complete the second dose of Rotarix in Rotarix villages and all those from non-Rotarix villages.<br><br>Follow-up period: Person-time will be counted from 26 weeks of age until two years (731 days) of age or March 31, 2011, whichever comes first.
**"Direct effectiveness" populations**	
	**mITT analysis**<br>Those included: Infants in the "**overall effectiveness-primary analysis**" population from Rotarix villages plus those infants residing in Rotarix study villages who are between 6 and 20 weeks of age on Apr 1, 2009 (ICDDR,B service area) or on Nov 1, 2008 (Govt service area), regardless of receipt of Rotarix.<br><br>Follow-up period: Person-time will be counted from six weeks of age until two years (731 days) of age or March 31, 2011, whichever comes

	first.<br>**ATP analysis**<br>Those included: Infants in the "**overall effectiveness-primary analysis**" population from Rotarix villages plus those infants residing in Rotarix study villages who are between 6 and 20 weeks of age on Apr 1, 2009 (ICDDR,B service area) or on Nov 1, 2008 (Govt service area), regardless of receipt of Rotarix.<br><br>Follow-up period: Person-time will be counted from 26 weeks of age until two years (731 days) of age or March 31, 2011, whichever comes first.
**"Indirect effectiveness" populations**	
	Three groups are definable for analysis of indirect effects.<br>**Indirect Population 1**<br>Those included: Infant and child residents of Matlab who are at least 20 weeks of age as of Apr 1, 2009 (ICDDR,B service area villages) or Nov 1, 2008 (Govt service area villages) or in-migrants to the study area who are between 20 weeks and 2 years of age during the study period. Treatment group assignment for these children will be determined by village of residency at the time that first allows them to be included in this population.<br><br>Follow-up period: Person-time will be counted from Apr 1, 2009 (ICDDR,B service area villages) or Nov 1, 2008 (Govt service area villages) or date of in-migration until two years of age (731 days) of age or March 31, 2011, whichever comes first.<br><br>**Indirect Population 2**<br>Those included: Infants who resided in Matlab between 6 and 20 weeks of age after Apr 1, 2009 (ICDDR,B service area) or after Nov 1, 2008 (Govt service area).<br><br>Follow-up period: Person-time will be counted from Apr 1, 2009 (ICDDR,B service area villages) or Nov 1, 2008 (Govt service area villages) or date of in-migration until receipt of OPV1, the child turns two years of age (731 days), or March 31, 2011, whichever comes first.<br><br>**Indirect Population 3**<br>Those included: All infants or children resident of the study area aged between 6 weeks and 2 years at some point during the study period.<br><br>Follow-up period: All person-time contributed during the study period. The analysis will account for receipt of vaccine (this will include any

	indirect effects that also accrue to those who receive Rotarix, which is not 100% protective).

3.3 Baseline Characteristics

Summaries will be provided to describe the subject populations in this study by study arm. Only subjects with data will be included in the summaries, i.e. missing data will be counted as missing.

We will report baseline characteristics in two ways:

1) Data on infants and children who are aged 6 weeks to 2 years residing in the service areas on November 1, 2008 (April 1, 2009)
2) Data on infants who are part of the Overall cohort of those who turn 6 weeks of age over the course of the entire study period, or who in-migrated at an age less than 24 weeks

Also by age groups: 6 weeks to 1 year, 1 to 2 years. Also by sex. Also, the proportions of children aged 12-23 months within each randomization unit with 1, 2, and 3 OPV doses. Also, by SES quintile…

3.4 Case Definitions and Severity Score

3.4.1

- Seeking care for treatment of diarrhea at a treatment facility in Matlab, Nayargaon or Kalibazar.
- Age less than 2 years of age.
- Diarrhoea: three or more looser than normal stools in a 24 hour period.
- Acute rotavirus diarrhoea: an acute diarrhoeal illness episode in which rotavirus is identified in a stool sample collected as soon as possible but no later than 7 days after admission to the medical facility.
- Fever: rectal temperature ≥ 38°C

Severe rotavirus Diarrhea:
Defined as a case of acute rotavirus diarrhea, as above, with a Vesikari score of 11 or greater; next most important will be for 15 greater.

Vesikari score (slightly modified for lack of full follow up of cases):

The completion of the diary card for each GE episode will allow the assessment of the intensity by using a 20-point scale. In this system, points will be assigned according to duration and intensity of diarrhoea and vomiting, the intensity of fever, presence of dehydration or

hospitalisation for each episode of GE as shown below:

Sign/Symptom		Points
**Duration of looser than normal stools (days)**		
1-4		1
5		2
≥ 6		3
**Maximum number of looser than normal stools /24 hours**		
1-3		1
4-5		2
≥ 6		3
**Duration of vomiting (days)**		
1		1
2		2
≥ 3		3
**Maximum number of episodes of vomiting/24 hours**		
1		1
2-4		2
≥ 5		3
**Fever***		
**Rectally**	**Axillary**	
37.1 – 38.4°C	36.6 – 37.9°C	1
38.5 – 38.9°C	38.0 – 38.4°C	2
≥ 39°C	≥ 38.5°C	3
**Dehydration****		
1-5%		2
≥ 6%		3
**Treatment**		
Rehydration		1
Hospitalisation		2

∗ The highest temperature recorded during the episode will be scored.
** Dehydration will be assessed clinically based on World Health Organisation criteria.

Adverse Events and Serious Adverse Events

Overall mortality in the two types of study areas will be compared with Kaplan-Meier curves among those in the Overall population, up to earliest of: out-migration, 24 months of age, Mar 31, 2010. Statistical inference will proceed via Cox models with gamma frailty.

3.5 Statistical Analyses

Here we describe the analyses according to the study aims (in italics) listed in the protocol;

Vaccine population effectiveness parameters (with 95% CI) will be calculated as [1 - (incidence rate of rotavirus among vaccine villages/incidence rate among the villages without vaccine)] x 100.

For all analyses, we will handle migration of infants among study villages as follows: Regardless of the analysis, an infant or child’s village of residence will be the one in which he or she first

enters the Overall population. This village assignment will be used regardless of migration within any of the study areas.

Primary Aim

To evaluate the population (overall) (Halloran, et al., 1997) effectiveness of Rotarix in reducing rates of acute diarrhoea due to rotavirus, as detected by patients admitted to a study treatment facility, among all infants and young children from villages where Rotarix is introduced compared to among those from villages where Rotarix is not introduced.

Main analysis, on Overall population: first episodes of rotavirus diarrhea. A Poisson regression with Pearson scale parameter (calculated at the village level) will be fit, offset with person-years of exposure, with a dummy covariate for village randomization arm status and a dummy covariate for villages in the Matlab service area (corresponding to the stratified randomization).

Note that this overall effectiveness is a function of vaccine efficacy and vaccine coverage. Since over 90% coverage is expected, this will not be much different from a total effects analysis. Also, if there are any indirect effects that act to reduce transmission, these may exert a differential effect over time, as it is not until near the end of the study period that the entire population of children under 2 years of age will be saturated with vaccine.

Additional analysis: same as main analysis, but including ***all*** episodes of rotavirus diarrhea, not just first episodes. The scale parameter at the randomization unit level also accounts for all within-child correlation, so the same analysis approach can be used.

Secondary Aims

1. To evaluate the total effectiveness of Rotarix in reducing rates of acute diarrhoea due to rotavirus, as detected by patients admitted to a study treatment facility, among vaccinated infants and young children from villages where Rotarix is introduced compared to unvaccinated infants and young children from villages where Rotarix is not introduced.

Main analysis, “overall effectiveness” primary analysis population: with the modification of starting observation time at 12 weeks instead of 6 weeks of age. A Poisson regression with Pearson scale parameter (calculated at the village level) will be fit, offset with person-years of exposure, with a dummy covariate for village randomization arm status and a dummy covariate for villages in the Matlab service area.

Additional analyses:

a. As in 1., but for all (not just first) cases of rotavirus diarrhea.
b. As in 1. and 1.a, but for the mITT population.

2. To evaluate the indirect effectiveness of Rotarix in reducing rates of acute diarrhoea due to rotavirus, as detected by patients admitted to a study treatment facility, among unvaccinated

infants and young children from villages where Rotarix is introduced compared to those from villages where Rotarix is not introduced.

Analyses are given according to the populations a, b, c given above.

a) Those aged at least 20 weeks as of Nov 1, 2008 (Apr 1, 2009), with censoring at earliest of 2 years of age or Mar 31, 2001, whichever comes first. Also included are those who in-migrate between ages 20 weeks-2 years. Poisson regression as described above will be used; all cases (first and subsequent) will be used.

b) Those in the Overall population, but with censoring at first OPV dose. Because of the high coverage, this will not comprise many person-years.
Poisson regression as described above will be used; all cases (first and subsequent) will be used.

c) All person-time contributed by infants or children who were between 6 weeks and 2 years of age at some point during the study period.

A detailed, yet "black box" analysis will use a Cox regression model with calendar timeline, with baseline hazard stratified by age at entry: 6wks-6 months, 6 months-1 year, 1yr-2yrs. The first step is to determine, for each village, for each day of the study, the time-varying proportion of infants and young children (6wks to 2 years) who have received at least 1 dose of OPV. Then, for each village, we find the 3 dates corresponding to when the village first achieves the proportions: 0.25, 0.50, 0.75. Thus, at any point in time, we can categorize each village into one of four immunization coverage categories: 0-24%, 25-49%, 50-74%, and 75-99%. There may be some misclassification in that a village may reach 27% one day and drop to 20% a month later, but these variations will be too difficult to track, and should not have much effect.

Crossing these four categories with whether a village is a Rotarix or control village yields 8 time-varying categories, which can be represented by 7 covariates:
$R_{it}^{0-24}, R_{it}^{25-49}, R_{it}^{50-74}, R_{it}^{75+}, C_{it}^{25-49}, C_{it}^{50-74}, C_{it}^{75+}$
where *R* and *C* correspond to Rotarix and control villages, respectively, *i* is for the *i*th infant, and *t* is for the infant's age in days. For example, if a given infant living in a Rotarix village is 87 days old on a given day, and on that day the village has already reached 25% coverage but not yet 50% coverage as defined above, then R_{it}^{25-49} =1 and the other 7 covariates are coded as 0. Note the reference category is 0-24% coverage in a control village. In addition, we will track the immunization status of each infant with the variable Vac_{it} which starts as 0 and becomes 1 upon first OPV. This variable is not of much interest—its coefficient will estimate any protective effect due to selection for vaccination. Crossing this term with village type of residence, denoted by X_i (=1 for Rotarix, 0 for control) yields an interaction term that indicates the individual-level protection afforded by Rotarix. Putting this all together into a specification of the hazard function, we get:

$$\lambda_i(t) = \lambda_0(t)\exp(\beta_1 Vac_{it} + \beta_2 X_i Vac_{it} + \beta_3 R_{it}^{0-24} + \beta_4 R_{it}^{25-49} + \beta_5 R_{it}^{50-74} + \beta_6 R_{it}^{75+} + \beta_7 C_{it}^{25-49} + \beta_8 C_{it}^{50-74} + \beta_9 C_{it}^{75+})$$

where $\lambda_0(t)$ is the unspecified baseline hazard function. X_i does not appear by itself because there is no expected effect of whether a child lives in a Rotarix or control village *per se*, as that was determined through randomization.

The indirect effects at any given level of coverage are estimated by taking the difference of the relevant covariates. For example, $\exp(\beta_6 - \beta_9)$ gives the relative hazard of those in a highly Rotarix-immunized village compared to those in a highly OPV-only immunized village. Note there will not be a great deal of power for these comparisons, however.

It will also be advisable to add two time-varying dummy variables to track the three main seasons (see the seasonality discussion below).

The best way to analyze these data is to use a gamma frailty model that assigns a random intercept term to each village to account for within-village correlation. Stata has a function for this, although it will take some careful work to split up each child's observation time into a number of intervals to account for all the time-varying covariates. It may also be possible to use the SAS PROC SURVEYPHREG to estimate a robust variance to account for the correlation. The advantage to this approach is that SAS allows for continuously-varying time-varying covariates that are relatively easily coded. If this specification in SAS, that will be the preferred approach for analysis.

3. To evaluate the direct effectiveness of Rotarix in reducing rates of acute diarrhoea due to rotavirus, as detected by patients admitted to a treatment facility, among vaccinated infants and young children from villages where Rotarix is introduced compared to unvaccinated infants and young children from villages where Rotarix is introduced.

This direct effect is best estimated by β_2 in the above model, which is the remaining effect of Rotarix after accounting for the indirect effects. This will be run for the mITT population.

A second estimate will be provided by applying a Cox model to the Direct effect population (i.e. the mITT population only in Rotarix villages), with a time-varying covariate for receipt of first dose of Rotarix.

*4. To investigate the overall, total, indirect, and direct effectiveness of Rotarix in reducing rates of acute diarrhoea due to rotavirus and requiring at least one overnight stay and rehydration therapy (equivalent to WHO plan B or C) in a treatment facility*Perform the above analyses, but for this severity definition.

Two more definitions for severe diarrhea analyses will be used in each analysis:

1) Acute rotavirus diarrhea scoring Vesikari 11 or greater

2) Acute rotavirus diarrhea scoring Vesikari 15 or greater

Thus, we have a total of four effectiveness parameters according to varying degrees of severity: the above three, and the first definition of any acute rotavirus diarrhea.

5. To investigate the direct effectiveness and total effectiveness of Rotarix in reducing rates of all-cause acute diarrhoea, as detected by patients admitted to a treatment facility.

Same as above, but not restricted to rotavirus diarrhea or to first case.

7. Immunogenicity analyses: The proportions seroconverting after receipt of both doses will be calculated and compared. Reverse cumulative distribution curves will be drawn.

If there is a 3.5% cumulative incidence of rotavirus diarrhea, and 50% vaccine effectiveness, we expect only about 8 cases of rotavirus diarrhea in the immunogenicity subset, not enough to explore incidence as a function of immune response.

3.6 Exploratory Analyses

Further analyses focusing on the total effectiveness of Rotarix include the following:

a. Total effect analyses of rotavirus diarrhea, but adjusting for all relevant covariates (p<0.1 and [RR>1.5 or RR<0.67], in a forward stepwise model).
b. Total effect analyses of rotavirus diarrhea, but using a Cox survival model as in the indirect analyses above, but with a focus on covariates of interest. In particular, two time-varying dummy covariates will be used to track the three seasons: March through May; June through October; November through February. Two interaction terms for Rotarix * season will be added to look at variable effectiveness. (Note: although Bangladesh is land of the *Sadartu*, five dummy variables for these six seasons would be unwieldy).
   In addition, we will look for variable effectiveness by gender and SES quintile.

Finally, a 'check' analysis will be performed in the Overall population to compare rates of non-rotavirus diarrhea between study areas. Unless there are non-specific effects of Rotarix on these kinds of cases, we would expect the rates to be very similar.

4. INTERIM ANALYSIS

There are no interim analyses for efficacy or effectiveness in this study. About 6 months before the end of data collection, an analysis of historical data that were compiled before the study start will be carried out to estimate the design effect for the primary outcome. It is possible that if the estimated design effect were much larger than the one assumed for sample size calculations, the study could be extended accordingly.

This analysis of historical data will not be used in the evaluation of study estimates of efficacy.
