# Supplementary material for: Effectiveness of a live oral human rotavirus vaccine after programmatic introduction in Bangladesh: A cluster-randomized trial
Source: PLoS Med. 2017 Apr 18;14(4):e1002282. doi: 10.1371/journal.pmed.1002282 (PMC5395158; doi:10.1371/journal.pmed.1002282)

Analysis Report

Cluster-randomized trial of human rotavirus vaccine, Rotarix®, among children 6-weeks to 2 years of age in Matlab Bangladesh, 2008-2011.

Trial conducted by PATH, icddr,b, and Johns Hopkins University

Analysis here-in conducted by Jonathan D. Sugimoto and M. Elizabeth Halloran
Vaccine and Infectious Disease Division,
Fred Hutchinson Cancer Research Center

Table of Contents

Item	Page No.
Executive summary with summary table	2
Methods and Definitions for Vaccine Effects	4
Results tables for 6-week to 2 year-olds: Tables 1 to 6	20
Results tables for less-than 1 year-olds, who were older than 6-weeks: Tables 7 to 12	26
Results tables for 1 to 2 year-olds: Tables 13 to 18	31
Table 19: Number of villages that did not contribute person-time of risk, by vaccine parameter, outcome, and intervention group	36
Figure 7: Quarterly frequency of single-strain infections among study participants and the quarterly prevalence of specific G and P serotype combinations among these infections	39

Executive Summary

Background. Rotavirus infection is a significant cause of gastrointestinal illness worldwide, particularly among children under 2 years-of-age. Delivery of a rotavirus vaccine as part of the current Expanded Programme for Immunization (EPI) schedule for childhood vaccinations represents a cost-efficient mechanism for establishing routine rotavirus vaccination programs in low-resource settings. A randomized trial in rural Bangladesh assessed the feasibility and effectiveness of integrating a two dose regimen of rotavirus vaccine into routine EPI immunization visits at 6 and 20 weeks of age.

Materials and Methods. A cluster-randomized, non-placebo-controlled effectiveness trial of the monovalent human rotavirus vaccine (HRV) Rotarix® was conducted between November 2008 and March 2011 (study period). Within strata defined by service areas (icddr,b versus government administered), clusters of households (villages) were randomized to receive either the standard EPI schedule (comparison group) or an enhanced EPI schedule with HRV offered at the 6 and 20 week immunization visits (intervention group). Children residing in a study village and who were 6 to 24 weeks of age at some point during the study period were eligible to participate in the study. The primary study endpoint, acute rotavirus diarrhea (ARD) was defined as 3 or more looser-than-normal stools in a 24 hour period, with a stool specimen (collected at a medical care facility within 7 days of onset of diarrhea) testing positive for rotavirus. Secondary study endpoints included severe (overall Vesikari score >/=11) and very severe ARD (overall Vesikari score >/=15); and strain specific ARD (G1P8, G2P4, G9P8, G12P6, and G12P8, as well as infection with any G1 or P8 containing strain and infection with all other strains). Two negative control outcomes were defined as enterotoxic *E. coli* associated acute diarrhea (same clinical definition as ARD) with and without co-infection with rotavirus, respectively. Study endpoints were assessed through surveillance at medical care facilities that handle nearly all of the diarrheal illness in this population that requires medical attention. The overall, total, and indirect effectiveness of HRV delivered via regular EPI visits were estimated as 1 minus the incidence rate ratio estimated by Poisson regression, using a Pearson scale parameter.

Results. The table on the next page summarizes the estimated effects of HRV for the primary and each secondary study endpoint. The overall effectiveness of HRV toward preventing ARD was 29% (95% confidence limits [CL]: 11%, 43%). HRV's total (combined effects of direct and indirect protection) effectiveness (modified intention to treat) against ARD was 39% (95% CL: 21%, 53%). There was little evidence of any significant indirect protection afforded to children 6-weeks to 2 years of age who did receive HRV.

Interestingly, the overall effectiveness are lower for severe and for very severe ARD, relative to ARD. Relative to estimates for all ARD, HRV is estimated to be substantially more protective (overall and total) against ARD caused by G1P8 and G12P8 viruses, which is encouraging given that this monovalent vaccine contains a G1P8 antigen. There is also some evidence suggesting that Rotarix® may confer some heterotypic protection against ARD associated with a non-G1 and non-P8 serotype rotavirus infection, particularly G2P4.

The effects of HRV on the two negative control outcomes suggest that, though not statistically significant, there is a trend toward HRV protecting against ETEC-associated acute diarrhea. Given that HRV protection against ETEC-associated acute diarrhea is biologically implausible, this trend may represent evidence that either 1) there is some level of correlation between the risks of ETEC and rotavirus associated diarrhea or 2) that bias was introduced post-randomization. If the latter, then the effectiveness of HRV is over-estimated here.

Discussion. Rotarix® confers a moderate level of protection against acute and severe acute rotavirus diarrhea when delivered as part of the 1st and 2nd post-birth EPI visits. Protection is greatest against homotypic infection with G1P8 and G12P8 rotaviruses.

Summary table of the effectiveness (%, 95% confidence limits [CL]) of Rotarix® human rotavirus vaccine against acute rotavirus diarrhea (ARD), severe ARD, very severe ARD, and acute Enterotoxic *E. coli* diarrhea (excluding and including co-infections with rotavirus) among 6-week to 2 years-olds in Matlab, Bangladesh, 2008-2011.

Vaccine Effect	Age Group	Outcome: Acute Rotavirus Diarrhea (ARD)	Severe ARD (Vesikari >/=11)	Very Severe ARD (Vesikari >/=15)	Acute Enterotoxic *E. coli* Diarrhea, <u>Excluding</u> rotavirus co-positive cases	Acute Enterotoxic *E. coli* Diarrhea, <u>Including</u> rotavirus co-positive cases
Overall Effectiveness 1	6-weeks to 2 year	**29.0** (11.3, 43.1)	**22.9** (-0.2, 40.7)	**12.8** (-32.1, 42.5)	**19.2** (-31.4, 50.3)	**5.6** (-40.7, 36.6)
	6-weeks to 0.9 year	**33.0** (14.6, 47.4)	**30.1** (7.1, 47.4)	**18.9** (-30.4, 49.6)	**21.5** (-37.6, 55.2)	**13.9** (-34.7, 44.9)
	1.0 to 1.9 years	**12.2** (-37.4, 43.9)	**-7.7** (-81.8, 36.2)	**-10.4** (-155.8, 52.4)	**13.8** (-108.0, 64.2)	**-22.6** (-149.2, 39.7)
Total Effectiveness, modified intention to treat (mITT) 1	6-weeks to 2 year	**38.7** (20.6, 52.7)	**37.4** (16.1, 53.3)	**37.8** (0.3, 61.2)	**5.3** (-55.4, 42.3)	**4.0** (-46.5, 37.1)
	6-weeks to 0.9 year	**42.4** (23.5, 56.5)	**42.9** (21.5, 58.5)	**45.2** (4.8, 68.4)	**13.0** (-51.2, 50.0)	**13.4** (-39.2, 46.1)
	1.0 to 1.9 years	**27.9** (-15.6, 55.1)	**19.7** (-39.2, 53.6)	**15.4** (-107.0, 65.5)	**-12.5** (-184.9, 55.6)	**-25.0** (-184.4, 45.1)
Total Effectiveness, according to protocol (ATP) 1	6-weeks to 2 year	**41.4** (23.2, 55.2)	**42.8** (22.1, 57.9)	**39.3** (3.2, 61.9)	**19.1** (-33.9, 51.2)	**17.3** (-26.6, 45.9)
	6-weeks to 0.9 year	**45.2** (26.3, 59.3)	**48.0** (27.0, 63.0)	**45.9** (3.6, 69.6)	**26.7** (-30.3, 58.7)	**25.7** (-19.8, 53.9)
	1.0 to 1.9 years	**28.9** (-15.6, 56.3)	**25.8** (-29.5, 57.5)	**18.8** (-92.3, 65.7)	**1.9** (-145.7, 60.9)	**-8.7** (-144.6, 51.7)
Indirect Effectiveness 1, among those too old for vaccination	6-weeks to 2 year	**-1.2** (-43.9, 28.8)	**-5.7** (-58.6, 29.6)	**15.8** (-83.6, 61.4)	**-46.7** (-153.5, 15.2)	**-28.6** (-175.2, 39.9)
	6-weeks to 0.9 year	**1.6** (-96.2, 50.7)	**1.6** (-114.6, 54.9)	**29.9** (-127.1, 78.4)	**-35.3** (-224.8, 43.7)	**-29.8** (-315.6, 59.4)
	1.0 to 1.9 years	**-3.4** (-44.5, 26.0)	**-10.6** (-58.1, 22.6)	**1.8** (-73.4, 44.4)	**-52.7** (-199.6, 22.2)	**-28.0** (-127.5, 28.0)
Indirect Effectiveness 2, among those age-eligible for vaccination	6-weeks to 2 year	**9.2** (-40.8, 41.5)	**0.9** (-68.6, 41.8)	**-144.3** (-503.0, 1.0)	**54.4** (-25.1, 83.4)	**45.9** (-30.4, 77.5)
	6-weeks to 0.9 year	**-6.1** (-78.0, 36.7)	**-4.7** (-90.3, 42.4)	**-341.1** (-1930.6, 4.2)	**53.3** (-48.1, 85.3)	**52.3** (-36.4, 83.3)
	1.0 to 1.9 years	**35.7** (-37.4, 70.0)	**11.6** (-117.6, 64.1)	**-1.2** (-248.4, 70.6)	**50.1** (-76.9, 85.9)	**-0.1** (-168.8, 62.8)

OPV, oral poliovirus vaccine

Methods and Materials

Study outcome definitions.
The clinical case definition for acute diarrhea will be as follows: three or more looser-than-normal stools in a 24-hour period for which care is sought at a medical facility. The effect parameters will be estimated for nine rotavirus-associated outcome definitions: acute rotavirus diarrhea (ARD), severe ARD (overall Vesikari score >/= 11), very severe ARD (overall Vesikari score >/= 15), and strain specific ARD (G1P8, G2P4, G9P8, G12P6, and G12P8, as well as infection with any G1 or P8 containing strain and infection with all other strains). To asses for sources of bias, we will also estimate effect parameters for a negative control condition: acute enterotoxic *E. coli* (ETEC) associated diarrhea (same clinical definition for ARD, but replaces laboratory confirmation of rotavirus infection with laboratory confirmation of infection with ETEC). Two version of the negative control outcome will be considered: 1) including only acute diarrhea cases that were ETEC positive, but rotavirus negative and 2) including all acute diarrhea cases that were ETEC positive (i.e., includes co-infections with rotavirus).

Parameter Definitions. The following section defines the effect parameters estimated for the HRV vaccine or in support of the estimation of these vaccine effects. The term HRV vaccine will be employed as short-hand for the entire intervention being assessed, *i.e.*, the delivery of Rotarix® human rotavirus vaccine as part of post-birth visits 1 and 2 of the standard EPI schedule in Bangladesh. Receipt of a specific dose of OPV will be considered a marker for completion of the EPI visit during which that dose is scheduled to be delivered. Vaccine effects will be estimated as 1 minus the incidence rate ratio (estimated by Poisson regression) for the disease outcome in question among the vaccine group versus the comparison group. The following section will define the person-time contributed to each of these two groups. Parameters that condition on receipt of one or more HRV or OPV doses will only include immunizations received between 6 and 23 weeks of age (inclusive).

Administration of HRV off the protocol-prescribed schedule. Participants in Rotarix villages who received HRV doses off the protocol-prescribed schedule (see Table 20 for numbers), *i.e.*, HRV1 with OPV2 and/or HRV2 with OPV3, are represented in Figure 1 and all similar figures as being members of the box that they would have been assigned to should they have received the same HRV doses, but on the protocol-prescribed schedule. For analysis purposes, individuals who received HRV off protocol prescribed schedule are not considered to contribute person-time of outcome risk before the true administration date for HRV, unless the particular analysis does not condition the contribution of person-time on the date of HRV/OPV administration.

Figure 1. Grouping of study eligible children by trial-randomized treatment arm (Groups A and B) and by reported receipt (+) or non-receipt (-) of the oral poliovirus vaccine (OPV) and Rotarix® human rotavirus vaccine (HRV) by the Expanded Programme on Immunization (EPI) scheduled dose (1 or 2). Receipt of OPV or HRV must occur while the child is resident in a Matlab study village. Children were randomized by village to receive the standard EPI vaccines at post-birth visits 1 and 2 (Non-Rotarix Villages, Group B) or the same standard EPI vaccines, plus one HRV dose per visit (Rotarix Villages, Group A). To illustrate the fact that children residing in non-Rotarix villages were not offered a control vaccine or placebo (i.e., in addition to OPV), the HRV rows for Group B are left blank. Participants in Rotarix villages who received HRV doses off the protocol-prescribed schedule (see Table 20 for numbers), *i.e.*, HRV1 with OPV2 and/or HRV2 with OPV3, are represented in this diagram and all similar figures as being members of the box that they would have been assigned to should they have received the same HRV doses, but on the protocol-prescribed schedule. For analysis purposes, individuals who received HRV off protocol prescribed schedule are not considered to contribute person-time of outcome risk before the true administration date for HRV, unless the particular analysis does not condition contribution of person-time on the date of HRV/OPV administration.

Rotarix Villages

A1	A2	A3
OPV 1 +	OPV 1 +	OPV 1 -
HRV 1 +	HRV 1 +	HRV 1 -
OPV 2 +	OPV 2 -	OPV 2 +
HRV 2 +	HRV 2 -	HRV 2 +

A4	A5	A6	A7
OPV 1 +	OPV 1 +	OPV 1 -	OPV 1 -
HRV 1 -	HRV 1 -	HRV 1 -	HRV 1 -
OPV 2 +	OPV 2 -	OPV 2 +	OPV 2 -
HRV 2 -	HRV 2 -	HRV 2 -	HRV 2 -

Non-Rotarix Villages

B1	B2	B3	B4
OPV 1 +	OPV 1 +	OPV 1 -	OPV 1 -
OPV 2 +	OPV 2 -	OPV 2 +	OPV 2 -

I. Overall effectiveness of HRV among children 6 weeks to 2 years of age

Figure 2. Overall effectiveness parameter definitions

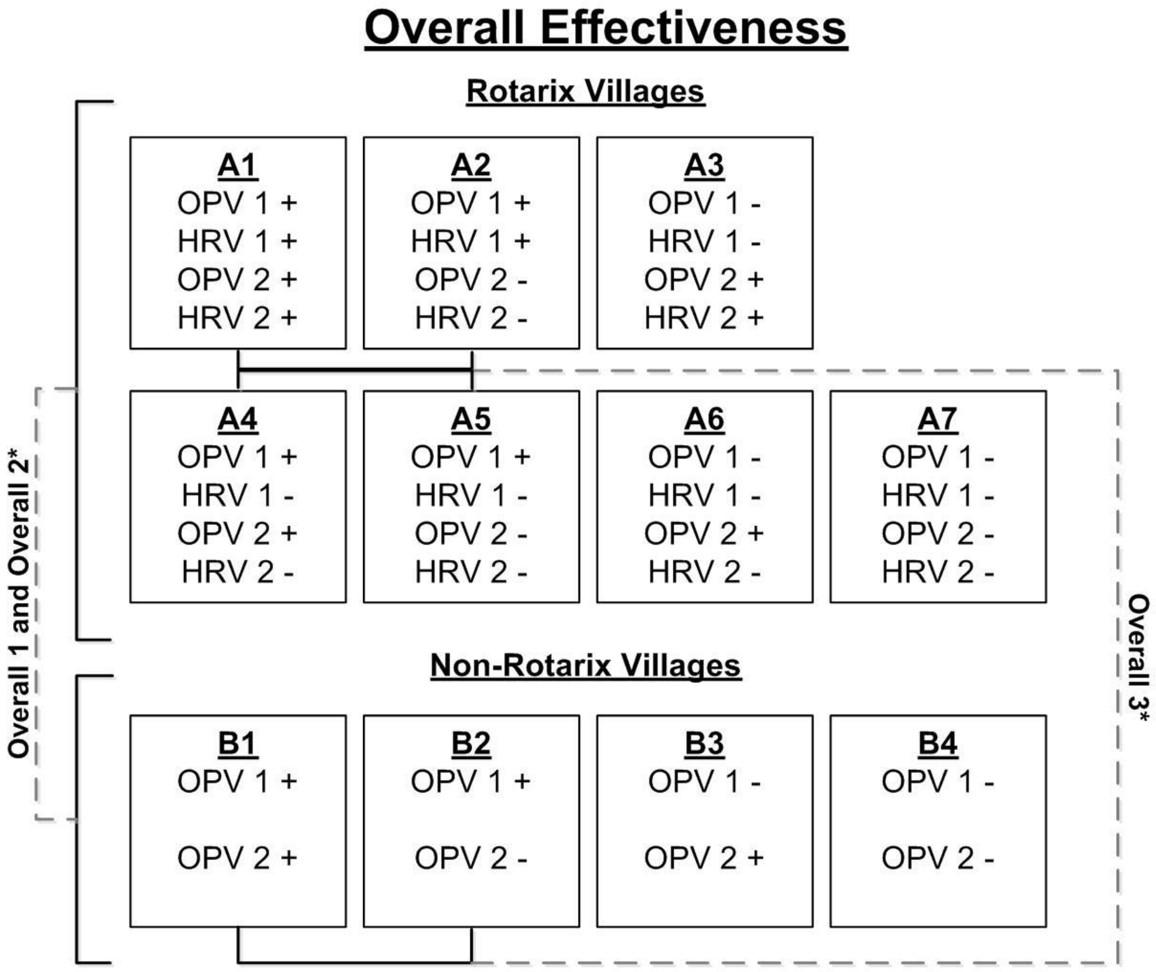

* In addition to children who turn 6-weeks while resident in a participating village during the study period (Overall 1 and all other vaccine effect parameter definitions), this parameter definition includes all otherwise eligible children who turned 6 weeks of age before the start of the study and were still under 21 weeks of age at that time point.

Overall Effectiveness 1. Primary analysis

Vaccine group:
Groups A1-A7 from Figure 1
Person-time included: Person-days accrued between 43 and 731 days of age among children resident in a Rotarix-village at 6 weeks-of-age between the beginning (April 1, 2009 for icddr,b service area and November 1, 2008 for government service area) and the end (March 31, 2011) of study follow-up. Plus, person-days accrued between 43 and 731 days of age among children who in-migrate (from outside of the Matlab study area) into a Rotarix-village while between 6 and 20 weeks-of-age between the beginning (April 1, 2009 for icddr,b service area and November 1, 2008 for government service area) and the end (March 31, 2011) of study follow-up. The first occurrence of the outcome of interest will lead to truncation of the person-time contributed at the date of hospital admission for the episode of illness.

<u>Comparison group:</u>
Groups B1-B4 from Figure 1
Person-time included: Person-days accrued between 43 and 731 days of age among children resident in a Non-Rotarix-village at 6 weeks-of-age between the beginning (April 1, 2009 for icddr,b service area and November 1, 2008 for government service area) and the end (March 31, 2011) of study follow-up. Plus, person-days accrued between 43 and 731 days of age among children who in-migrate (from outside of the Matlab study area) into a Non-Rotarix-village while between 6 and 20 weeks-of-age between the beginning (April 1, 2009 for icddr,b service area and November 1, 2008 for government service area) and the end (March 31, 2011) of study follow-up. The first occurrence of the outcome of interest will lead to truncation of the person-time contributed at the date of hospital admission for the episode of illness.

Overall Effectiveness 2. Add the residents ages 6-weeks-and-one-day to 20 weeks on the first day of the study

<u>Vaccine group:</u>
Groups A1-A7 from Figure 1
Person-time included: Person-days accrued between 43 and 731 days of age among children resident in a Rotarix-village while 6 to 20 weeks-of-age any time between the beginning (April 1, 2009 for icddr,b service area and November 1, 2008 for government service area) and the end (March 31, 2011) of study follow-up. The first occurrence of the outcome of interest will lead to truncation of the person-time contributed at the date of hospital admission for the episode of illness.

<u>Comparison group:</u>
Groups B1-B4 from Figure 1
Person-time included: Person-days accrued between 43 and 731 days of age among children resident in a Non-Rotarix-village while 6 to 20 weeks-of-age any time between the beginning (April 1, 2009 for icddr,b service area and November 1, 2008 for government service area) and the end (March 31, 2011) of study follow-up. The first occurrence of the outcome of interest will lead to truncation of the person-time contributed at the date of hospital admission for the episode of illness.

Overall Effectiveness 3. Restrict the Overall Effectiveness 2 population to those who received Dose 1 of OPV during the study period

<u>Vaccine group:</u>
Groups A1, A2, A4, and A5 from Figure 1
Person-time included: Among recipients of OPV dose 1, person-days accrued between date of OPV1 receipt and 731 days of age among children resident in a Rotarix-village while 6 to 20 weeks-of-age any time between the beginning (April 1, 2009 for icddr,b service area and November 1, 2008 for government service area) and the end (March 31, 2011) of study follow-up. The first occurrence of the outcome of interest will lead to truncation of the person-time contributed at the date of hospital admission for the episode of illness.

<u>Comparison group:</u>
Groups B1 and B2 from Figure 1
Person-time included: Among recipients of OPV dose 1, person-days accrued between date of OPV1 receipt and 731 days of age among children resident in a Non-Rotarix-village while 6 to 20 weeks-of-age any time between the beginning (April 1, 2009 for icddr,b service area and November 1, 2008 for government service area) and the end (March 31, 2011) of study follow-

up. The first occurrence of the outcome of interest will lead to truncation of the person-time contributed at the date of hospital admission for the episode of illness.

II. Total effectiveness of HRV among children 6 weeks to 2 years of age

This set of analyses conditions a child's eligibility to contribute to the analysis on their receipt of OPV dose 1 (modified intention to treat) or on their receipt of both OPV dose 1 and 2 (according to protocol). Based upon assumptions detailed and tested elsewhere, eligibility in the Rotarix-villages is further restricted to receipt of the HRV dose associated with each of the OPV doses upon which eligibility is already conditioned.

Figure 3. Total effectiveness parameter definitions for modified intention to treat (Panel A) and according to protocol populations (Panel B)
A)

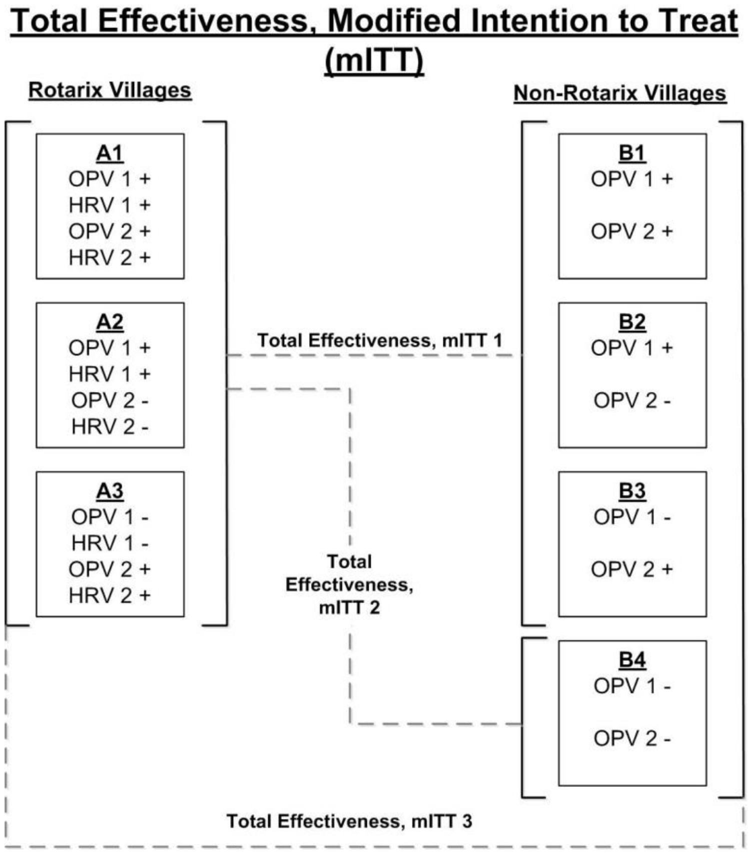

B)

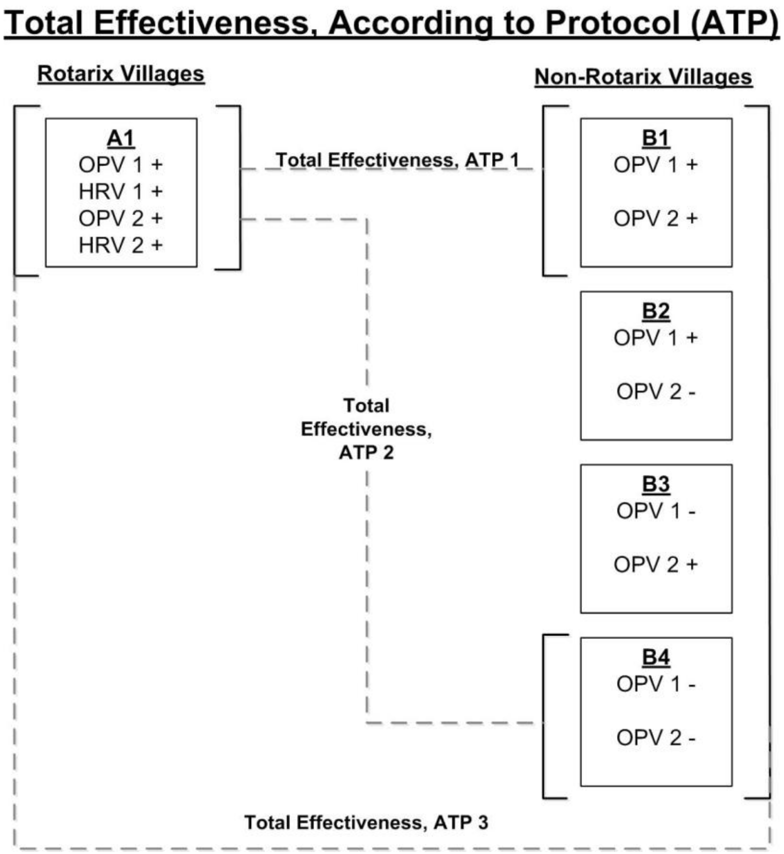

II.a. Modified intention to treat

Total Effectiveness, mITT 1. Comparison group is recipients of OPV1 or OPV2 in Non-Rotarix villages

<u>Vaccine group:</u>
Groups A1-A3 from Figure 1
Person-time included: Among recipients of an HRV dose 1 or dose 2 AND an OPV dose 1 or dose 2, person-days accrued between date of first receipt of OPV (minimum of OPV1 and OPV2 dates) and 731 days of age among children resident in a Rotarix-village while 6 to 20 weeks-of-age any time between the beginning (April 1, 2009 for icddr,b service area and November 1, 2008 for government service area) and the end (March 31, 2011) of study follow-up. The first occurrence of the outcome of interest will lead to truncation of the person-time contributed at the date of hospital admission for the episode of illness.

<u>Comparison group</u>:
Groups B1-B3 from Figure 1
Person-time included: Among recipients of an OPV dose 1 or dose 2, person-days accrued between date of first receipt of OPV (minimum of OPV1 and OPV2 dates) and 731 days of age among children resident in a Non-Rotarix-village while 6 to 20 weeks-of-age any time between the beginning (April 1, 2009 for icddr,b service area and November 1, 2008 for government service area) and the end (March 31, 2011) of study follow-up. The first occurrence of the

outcome of interest will lead to truncation of the person-time contributed at the date of hospital admission for the episode of illness.

Total Effectiveness, mITT 2. Comparison group is residents of Non-Rotarix villages who did not receive OPV

Vaccine group:
Groups A1-A3 from Figure 1
Person-time included: Among recipients of an HRV dose 1 or dose 2 AND an OPV dose 1 or dose 2, person-days accrued between 43 and 731 days of age among children resident in a Rotarix-village while 6 to 20 weeks-of-age any time between the beginning (April 1, 2009 for icddr,b service area and November 1, 2008 for government service area) and the end (March 31, 2011) of study follow-up. The first occurrence of the outcome of interest will lead to truncation of the person-time contributed at the date of hospital admission for the episode of illness.

Comparison group:
Group B4 from Figure 1
Person-time included: Among residents of Non-Rotarix-villages who received neither OPV dose 1 nor dose 2, person-days accrued between 43 and 731 days of age among children resident in a Non-Rotarix-village while 6 to 20 weeks-of-age any time between the beginning (April 1, 2009 for icddr,b service area and November 1, 2008 for government service area) and the end (March 31, 2011) of study follow-up. The first occurrence of the outcome of interest will lead to truncation of the person-time contributed at the date of hospital admission for the episode of illness.

Total Effectiveness, mITT 3. Comparison group is all age-eligible residents of Non-Rotarix villages

Vaccine group:
Groups A1-A3 from Figure 1
Person-time included: Among recipients of an HRV dose 1 or dose 2 AND an OPV dose 1 or dose 2, person-days accrued between 43 and 731 days of age among children resident in a Rotarix-village while 6 to 20 weeks-of-age any time between the beginning (April 1, 2009 for icddr,b service area and November 1, 2008 for government service area) and the end (March 31, 2011) of study follow-up. The first occurrence of the outcome of interest will lead to truncation of the person-time contributed at the date of hospital admission for the episode of illness.

Comparison group:
Groups B1-B4 from Figure 1
Person-time included: Person-days accrued between 43 and 731 days of age among children resident in a Non-Rotarix-village while 6 to 20 weeks-of-age any time between the beginning (April 1, 2009 for icddr,b service area and November 1, 2008 for government service area) and the end (March 31, 2011) of study follow-up. The first occurrence of the outcome of interest will lead to truncation of the person-time contributed at the date of hospital admission for the episode of illness.

II.b. According to Protocol

Total Effectiveness, ATP 1. Comparison group is recipients of OPV1 and OPV2 in Non-Rotarix villages

Vaccine group:
Group A1 from Figure 1
Person-time included: Among recipients of both HRV doses and both OPV doses, person-days accrued between two weeks after the receipt of OPV dose 2 and 731 days of age among children resident in a Rotarix-village while 6 to 20 weeks-of-age any time between the beginning (April 1, 2009 for icddr,b service area and November 1, 2008 for government service area) and the end (March 31, 2011) of study follow-up. The first occurrence of the outcome of interest will lead to truncation of the person-time contributed at the date of hospital admission for the episode of illness.

Comparison group:
Group B1 from Figure 1
Person-time included: Among recipients of both OPV doses, person-days between two weeks after the receipt of OPV dose 2 and 731 days of age among children resident in a Non-Rotarix-village while 6 to 20 weeks-of-age any time between the beginning (April 1, 2009 for icddr,b service area and November 1, 2008 for government service area) and the end (March 31, 2011) of study follow-up. The first occurrence of the outcome of interest will lead to truncation of the person-time contributed at the date of hospital admission for the episode of illness.

Total Effectiveness, ATP 2. Comparison group is residents of Non-Rotarix villages who did not receive OPV

Vaccine group:
Group A1 from Figure 1
Person-time included: Among recipients of both HRV doses and both OPV doses, person-days accrued between 182 (26 weeks) and 731 days of age among children resident in a Rotarix-village while 6 to 20 weeks-of-age any time between the beginning (April 1, 2009 for icddr,b service area and November 1, 2008 for government service area) and the end (March 31, 2011) of study follow-up. The first occurrence of the outcome of interest will lead to truncation of the person-time contributed at the date of hospital admission for the episode of illness.

Comparison group:
Group B4 from Figure 1
Person-time included: Among residents of Non-Rotarix-villages who received neither OPV dose, person-days accrued between 182 (26 weeks) and 731 days of age among children resident in a Non-Rotarix-village while 6 to 20 weeks-of-age any time between the beginning (April 1, 2009 for icddr,b service area and November 1, 2008 for government service area) and the end (March 31, 2011) of study follow-up. The first occurrence of the outcome of interest will lead to truncation of the person-time contributed at the date of hospital admission for the episode of illness.

Total Effectiveness, ATP 3. Comparison group is all age-eligible residents of Non-Rotarix villages

Vaccine group:
Group A1 from Figure 1
Person-time included: Among recipients of both HRV doses and both OPV doses, person-days accrued between 182 (26 weeks) and 731 days of age among children resident in a Rotarix-village while 6 to 20 weeks-of-age any time between the beginning (April 1, 2009 for icddr,b service area and November 1, 2008 for government service area) and the end (March 31, 2011) of study follow-up. The first occurrence of the outcome of interest will lead to truncation of the person-time contributed at the date of hospital admission for the episode of illness.

Comparison group:
Groups B1-B4 from Figure 1
Person-time included: Person-days accrued between 182 (26 weeks) and 731 days of age among children resident in a Non-Rotarix-village while 6 to 20 weeks-of-age any time between the beginning (April 1, 2009 for icddr,b service area and November 1, 2008 for government service area) and the end (March 31, 2011) of study follow-up. The first occurrence of the outcome of interest will lead to truncation of the person-time contributed at the date of hospital admission for the episode of illness.

III. Direct effectiveness of HRV among children 6 weeks to 2 years of age

Figure 4. Direct effectiveness parameter definitions for modified intention to treat (Panel A) and according to protocol populations (Panel B)
A)

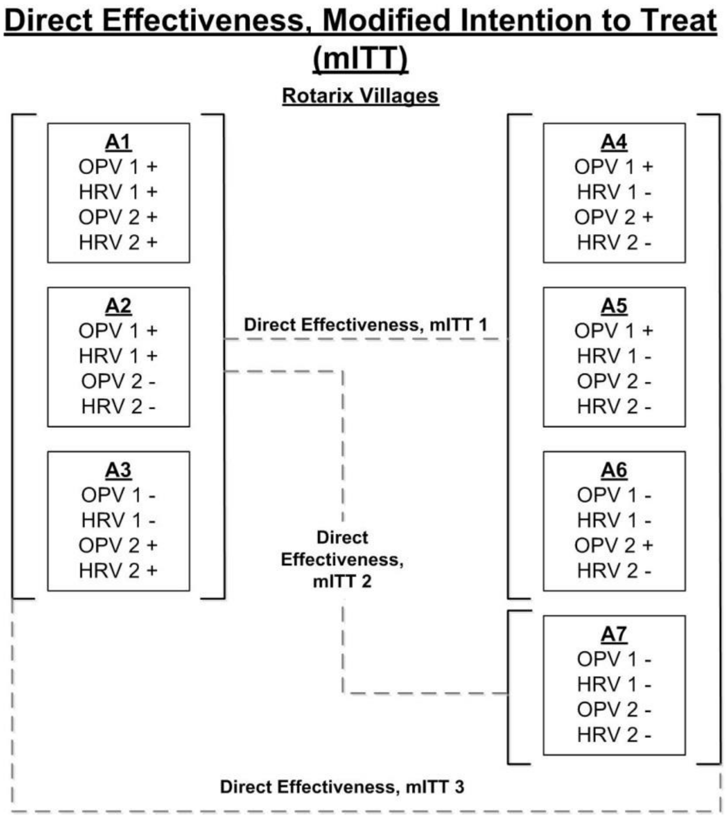

B)

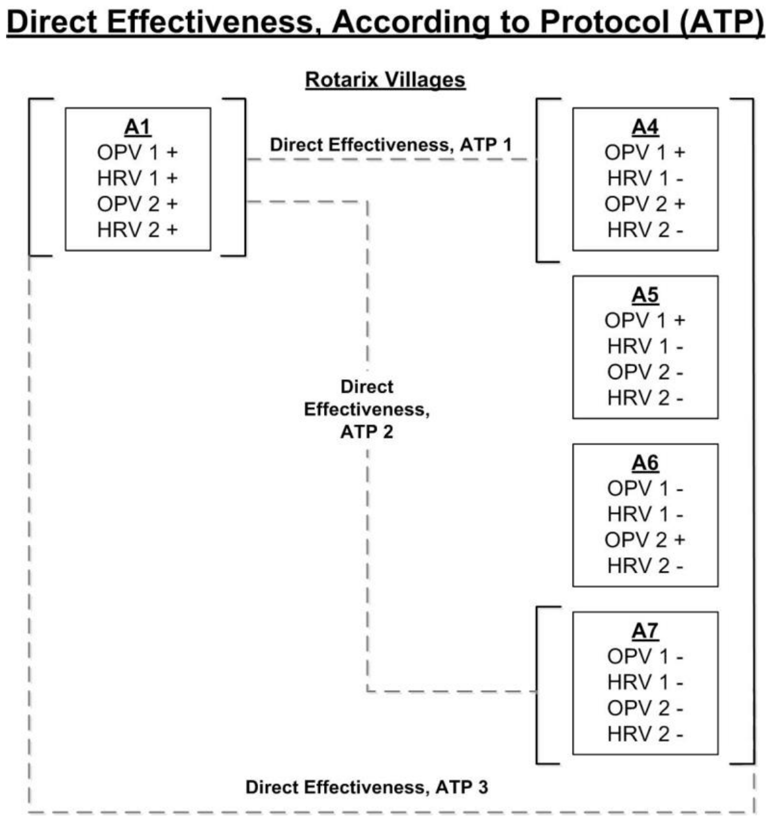

III.a. Modified intention to treat

Direct Effectiveness, mITT 1. Comparison group is recipients of OPV1 or OPV2 in Rotarix villages who did not receive HRV

Vaccine group:
Groups A1-A3 from Figure 1
Person-time included: Among recipients of an HRV dose 1 or dose 2 AND an OPV dose 1 or dose 2, person-days accrued between date of first receipt of OPV (minimum of OPV1 and OPV2 dates) and 731 days of age among children resident in a Rotarix-village while 6 to 20 weeks-of-age any time between the beginning (April 1, 2009 for icddr,b service area and November 1, 2008 for government service area) and the end (March 31, 2011) of study follow-up. The first occurrence of the outcome of interest will lead to truncation of the person-time contributed at the date of hospital admission for the episode of illness.

Comparison group:
Groups A4-A6 from Figure 1
Person-time included: Among recipients of an OPV dose 1 or dose 2 who did not receive any HRV, person-days accrued between date of first receipt of OPV (minimum of OPV1 and OPV2 dates) and 731 days of age among children resident in a Rotarix-village while 6 to 20 weeks-of-age any time between the beginning (April 1, 2009 for icddr,b service area and November 1, 2008 for government service area) and the end (March 31, 2011) of study follow-up. The first occurrence of the outcome of interest will lead to truncation of the person-time contributed at the date of hospital admission for the episode of illness.

Direct Effectiveness, mITT 2. Comparison group is residents of Rotarix villages who received neither OPV nor HRV

Vaccine group:
Groups A1-A3 from Figure 1
Person-time included: Among recipients of an HRV dose 1 or dose 2 AND an OPV dose 1 or dose 2, person-days accrued between 43 and 731 days of age among children resident in a Rotarix-village while 6 to 20 weeks-of-age any time between the beginning (April 1, 2009 for icddr,b service area and November 1, 2008 for government service area) and the end (March 31, 2011) of study follow-up. The first occurrence of the outcome of interest will lead to truncation of the person-time contributed at the date of hospital admission for the episode of illness.

Comparison group:
Group A7 from Figure 1
Person-time included: Among residents of Rotarix-villages who received neither OPV nor HRV, person-days accrued between 43 and 731 days of age among children resident in a Rotarix-village while 6 to 20 weeks-of-age any time between the beginning (April 1, 2009 for icddr,b service area and November 1, 2008 for government service area) and the end (March 31, 2011) of study follow-up. The first occurrence of the outcome of interest will lead to truncation of the person-time contributed at the date of hospital admission for the episode of illness.

Direct Effectiveness, mITT 3. Comparison group is all age-eligible residents of Rotarix villages who did not receive HRV

Vaccine group:
Groups A1-A3 from Figure 1
Person-time included: Among recipients of an HRV dose 1 or dose 2 AND an OPV dose 1 or dose 2, person-days accrued between 43 and 731 days of age among children resident in a Rotarix-village while 6 to 20 weeks-of-age any time between the beginning (April 1, 2009 for icddr,b service area and November 1, 2008 for government service area) and the end (March 31, 2011) of study follow-up. The first occurrence of the outcome of interest will lead to truncation of the person-time contributed at the date of hospital admission for the episode of illness.

Comparison group:
Groups A4-A7 from Figure 1
Person-time included: Among children who did not receive any HRV, person-days accrued between 43 and 731 days of age among children resident in a Rotarix-village while 6 to 20 weeks-of-age any time between the beginning (April 1, 2009 for icddr,b service area and November 1, 2008 for government service area) and the end (March 31, 2011) of study follow-up. The first occurrence of the outcome of interest will lead to truncation of the person-time contributed at the date of hospital admission for the episode of illness.

III.b. According to Protocol

Direct Effectiveness, ATP 1. Comparison group is recipients of OPV1 and OPV2 in Rotarix villages who did not receive any HRV

Vaccine group:
Group A1 from Figure 1
Person-time included: Among recipients of both HRV doses and both OPV doses, person-days accrued between two weeks after the receipt of OPV dose 2 and 731 days of age among children resident in a Rotarix-village while 6 to 20 weeks-of-age any time between the beginning (April 1, 2009 for icddr,b service area and November 1, 2008 for government service area) and the end (March 31, 2011) of study follow-up. The first occurrence of the outcome of interest will lead to truncation of the person-time contributed at the date of hospital admission for the episode of illness.

Comparison group:
Group A4 from Figure 1
Person-time included: Among recipients of both OPV doses who did not receive any HRV, person-days between two weeks after the receipt of OPV dose 2 and 731 days of age among children resident in a Rotarix-village at 6 weeks-of-age between the beginning (April 1, 2009 for icddr,b service area and November 1, 2008 for government service area) and the end (March 31, 2011) of study follow-up. The first occurrence of the outcome of interest will lead to truncation of the person-time contributed at the date of hospital admission for the episode of illness.

Direct Effectiveness, ATP 2. Comparison group is residents of Rotarix villages who received neither OPV nor HRV

Vaccine group:
Group A1 from Figure 1
Person-time included: Among recipients of both HRV doses and both OPV doses, person-days accrued between 182 (26 weeks) and 731 days of age among children resident in a Rotarix-village while 6 to 20 weeks-of-age any time between the beginning (April 1, 2009 for icddr,b service area and November 1, 2008 for government service area) and the end (March 31, 2011) of study follow-up. The first occurrence of the outcome of interest will lead to truncation of the person-time contributed at the date of hospital admission for the episode of illness.

Comparison group:
Group A7 from Figure 1
Person-time included: Among residents of Rotarix-villages who received neither OPV nor HRV, person-days accrued between 182 (26 weeks) and 731 days of age among children resident in a Rotarix-village while 6 to 20 weeks-of-age any time between the beginning (April 1, 2009 for icddr,b service area and November 1, 2008 for government service area) and the end (March 31, 2011) of study follow-up. The first occurrence of the outcome of interest will lead to truncation of the person-time contributed at the date of hospital admission for the episode of illness.

Direct Effectiveness, ATP 3. Comparison group is all age-eligible residents of Rotarix villages who did not receive any HRV

Vaccine group:
Group A1 from Figure 1
Person-time included: Among recipients of both HRV doses and both OPV doses, person-days accrued between 182 (26 weeks) and 731 days of age among children resident in a Rotarix-village while 6 to 20 weeks-of-age any time between the beginning (April 1, 2009 for icddr,b service area and November 1, 2008 for government service area) and the end (March 31, 2011) of study follow-up. The first occurrence of the outcome of interest will lead to truncation of the person-time contributed at the date of hospital admission for the episode of illness.

Comparison group:
Groups A4-A7 from Figure 1
Person-time included: Among children who did not receive any HRV, person-days accrued between 182 (26 weeks) and 731 days of age among children resident in a Rotarix-village while 6 to 20 weeks-of-age any time between the beginning (April 1, 2009 for icddr,b service area and November 1, 2008 for government service area) and the end (March 31, 2011) of study follow-up. The first occurrence of the outcome of interest will lead to truncation of the person-time contributed at the date of hospital admission for the episode of illness.

IV. Indirect effectiveness of HRV

Indirect Effectiveness 1. Among residents 20 weeks to 2 years of age at the start of the study period or in-migrating within the same age range

Vaccine group:
Person-time included: Among children 20-weeks to 2 years of age and resident in a Rotarix-village at the start of the study (April 1, 2009 for icddr,b service area and November 1, 2008 for government service area) and children within the same age range who in-migrate during the study period, time accrued from the start of the study or in-migration to the first of the end (March 31, 2011) of study follow-up or two-years of age. The first occurrence of the outcome of interest will lead to truncation of the person-time contributed at the date of hospital admission for the episode of illness.

Comparison group:
Person-time included: Among children 20-weeks to 2 years of age and resident in a non-Rotarix-village at the start of the study (April 1, 2009 for icddr,b service area and November 1, 2008 for government service area) and children within the same age range who in-migrate during the study period, time accrued from the start of the study or in-migration to the first of the end (March 31, 2011) of study follow-up or two-years of age. The first occurrence of the outcome of interest will lead to truncation of the person-time contributed at the date of hospital admission for the episode of illness.

Indirect Effectiveness 2. Among all residents who did not received HRV and who received at least one OPV dose

Vaccine group:
Person-time included: Among children resident in a Rotarix-village while 6 to 20 weeks-of-age any time between the beginning (April 1, 2009 for icddr,b service area and November 1, 2008 for government service area) and the end (March 31, 2011) of study follow-up, the time accrued

from start of study or in-migration until the first of the receipt of the first non-birth dose of OPV, receipt of the first dose of Rotarix, two-years of age, or the end of the study follow-up. The first occurrence of the outcome of interest will lead to truncation of the person-time contributed at the date of hospital admission for the episode of illness.

Comparison group:
Person-time included: Among children resident in a non-Rotarix-village while 6 to 20 weeks-of-age any time between the beginning (April 1, 2009 for icddr,b service area and November 1, 2008 for government service area) and the end (March 31, 2011) of study follow-up, the time accrued from start of study or in-migration until the first of the receipt of the first non-birth dose of OPV, two-years of age, or the end of the study follow-up. The first occurrence of the outcome of interest will lead to truncation of the person-time contributed at the date of hospital admission for the episode of illness.

V. Other miscellaneous effects

Figure 6. Other effect parameter definitions

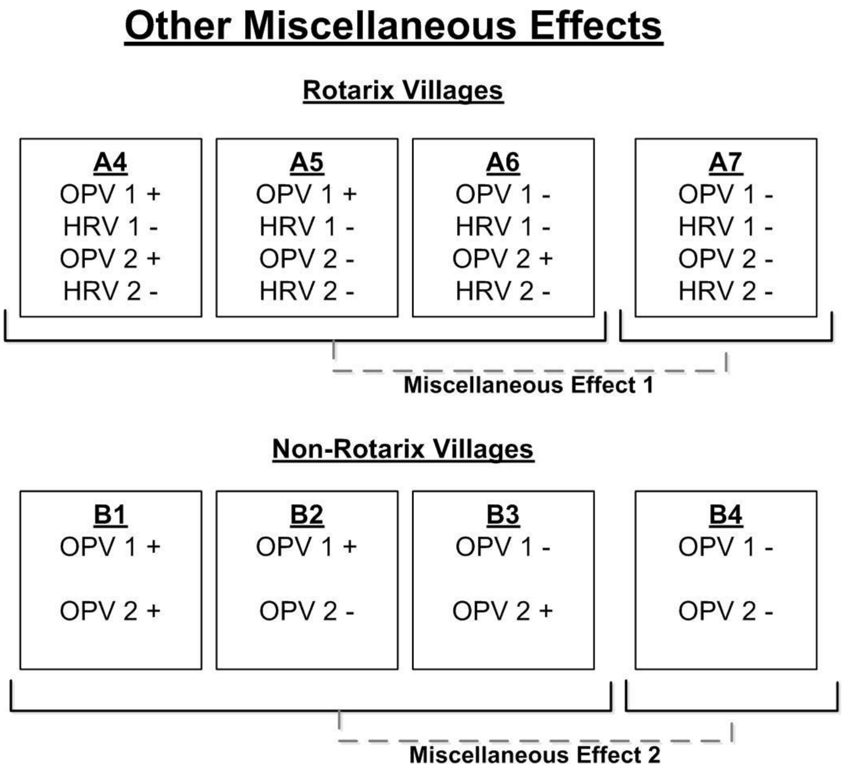

Miscellaneous Effect 1. Among residents of Rotarix villages, comparing individuals who received any OPV but no HRV to those who received neither OPV nor HRV

Vaccine group:
Groups A4-A6 from Figure 1
Person-time included: Among recipients of any OPV but no HRV, person-days accrued between 43 and 731 days of age among children resident in a Rotarix-village at 6 weeks-of-age between the beginning (April 1, 2009 for icddr,b service area and November 1, 2008 for government

service area) and the end (March 31, 2011) of study follow-up. The first occurrence of the outcome of interest will lead to truncation of the person-time contributed at the date of hospital admission for the episode of illness.

Comparison group:
Group A7 from Figure 1
Person-time included: Among children who did not receive any HRV or OPV, person-days accrued between 43 and 731 days of age among children resident in a Rotarix-village at 6 weeks-of-age between the beginning (April 1, 2009 for icddr,b service area and November 1, 2008 for government service area) and the end (March 31, 2011) of study follow-up. The first occurrence of the outcome of interest will lead to truncation of the person-time contributed at the date of hospital admission for the episode

Miscellaneous Effect 2. Among residents of non-Rotarix villages, comparing individuals who received any OPV to those who did not received any OPV

Vaccine group:
Groups B1-B3 from Figure 1
Person-time included: Among recipients of any OPV, person-days accrued between 43 and 731 days of age among children resident in a non-Rotarix-village at 6 weeks-of-age between the beginning (April 1, 2009 for icddr,b service area and November 1, 2008 for government service area) and the end (March 31, 2011) of study follow-up. The first occurrence of the outcome of interest will lead to truncation of the person-time contributed at the date of hospital admission for the episode of illness.

Comparison group:
Group B4 from Figure 1
Person-time included: Among children who did not receive any OPV, person-days accrued between 43 and 731 days of age among children resident in a non-Rotarix-village at 6 weeks-of-age between the beginning (April 1, 2009 for icddr,b service area and November 1, 2008 for government service area) and the end (March 31, 2011) of study follow-up. The first occurrence of the outcome of interest will lead to truncation of the person-time contributed at the date of hospital admission for the episode of illness.

Statistical methods

Primary analysis.
For each of the vaccine effect parameter definitions listed above, the total number of outcomes and the number of person-days of risk will be summarized at the village level, with the exception of the direct effectiveness related parameters, which will be summarized by strata defined within each village (see each parameter description for strata definitions). Poisson regression models are fit to the summarized case counts with the following indicator variables as independent predictors: intervention group indicator (Vaccine versus Comparison) and area (icddr,b versus government administered, included due to randomization of villages was stratified by area). The regression models were fit using the *glm* (generalized linear models) function in STATA v13.1, using a log link function, the Poisson family of models, the person-day totals as the offset variable (a measure of the level of exposure that generated the case counts in the dependent variable), and a Pearson Chi-squared scale parameter (village-level). Since estimation of the direct effectiveness parameters involved fitting Poisson regression models to case counts summarized by sub-village level strata, including a Pearson Chi-squared scale parameter was not feasible, due to the need to use the robust estimator of the variance to account for intra-

village correlation of observations. The difference between the incidence rates in non-Rotarix versus Rotarix villages was estimated in STATA v13.1 using the non-parametric approach described in Section 12.3.2 of Hayes and Moulton (2009), which accounts for the stratified nature of the randomization.

Analysis Results
The following Tables and Figures present the estimates of the vaccine effectiveness parameters for the Rotarix® human rotavirus vaccine (HRV) among children 6-weeks to 2 years of age in Matlab, Bangladesh, during 2008-2011. The primary parameters of interest are those related to the overall/population, total, and indirect effectiveness of HRV. Direct effectiveness estimates are provided for completeness, but please note the following important concern. It isn't entirely certain that direct effectiveness estimates derived from cluster-randomized trials represent unbiased estimates of a vaccine's true direct effectiveness. Given the lack of a control/placebo intervention in this particular trial, concerns about the potential for substantial bias in estimates of the direct effectiveness are especially acute.

Table 1. Overall, total, and indirect effectiveness (%, 95% confidence limits [95% CL]) of the human rotavirus vaccine (Rotarix®) against acute rotavirus diarrhea, severe ARD, and very severe ARD among children 6-weeks to 2 years-old in Matlab, Bangladesh, 2008-2011.

		Acute Rotavirus Diarrhea (ARD)				Severe ARD (Vesikari >/=11)				Very Severe ARD (Vesikari >/=15)			
**Effect**	**Group**	**N / PT**	**IR**	**VE (95% CL)**	**RD (95% CL)**	**N / PT**	**IR**	**VE (95% CL)**	**RD (95% CL)**	**N / PT**	**IR**	**VE (95% CL)**	**RD (95% CL)**
Overall 1[†]	Vacc	164/5857	2.80	**29.0**	**1.28**	128/5880	2.18	**22.9**	**0.83**	43/5933	0.72	**12.8**	**0.15**
	Comp	206/5026	4.10	(11.3, 43.1)	(0.31, 2.25)	149/5058	2.95	(-0.2, 40.7)	(-0.04, 1.71)	44/5121	0.86	(-32.1, 42.5)	(-0.28, 0.58)
Overall 2	Vacc	195/6960	2.80	**24.9**	**1.12**	151/6992	2.16	**20.4**	**0.74**	56/7054	0.79	**0.3**	**0.07**
	Comp	235/6031	3.90	(7.7, 38.9)	(0.24, 2.01)	172/6068	2.83	(-1.8, 37.7)	(-0.07, 1.54)	51/6147	0.83	(-44.1, 31.0)	(-0.31, 0.46)
Overall 3	Vacc	150/5512	2.72	**29.6**	**1.13**	116/5535	2.10	**25.3**	**0.78**	37/5586	0.66	**21.9**	**0.17**
	Comp	186/4620	4.03	(10.6, 44.6)	(0.15, 2.12)	136/4647	2.93	(2.0, 43.0)	(-0.09, 1.65)	41/4707	0.87	(-20.0, 49.2)	(-0.23, 0.56)
Total mITT 1[†]	Vacc	108/4735	2.28	**38.7**	**1.39**	80/4754	1.68	**37.4**	**0.99**	25/4790	0.52	**37.8**	**0.30**
	Comp	194/4998	3.88	(20.6, 52.7)	(0.47, 2.32)	142/5027	2.82	(16.1, 53.3)	(0.19, 1.79)	44/5089	0.86	(0.3, 61.2)	(-0.09, 0.70)
Total mITT 2	Vacc	112/4951	2.26	**68.5**	**7.79**	82/4970	1.65	**66.9**	**6.23**	26/5006	0.52	**57.8**	**1.80**
	Comp	24/320	7.50	(50.5, 79.9)	(2.59, 12.98)	17/327	5.20	(42.8, 80.8)	(1.06, 11.40)	4/336	1.19	(-54.3, 88.5)	(-1.60, 5.19)
Total mITT 3	Vacc	112/4951	2.26	**39.2**	**1.59**	82/4970	1.65	**38.7**	**1.15**	26/5006	0.52	**35.6**	**0.32**
	Comp	235/6031	3.90	(22.3, 52.4)	(0.70, 2.48)	172/6068	2.83	(18.4, 53.9)	(0.36, 1.93)	51/6147	0.83	(-1.0, 59.0)	(-0.04, 0.69)
Total ATP 1[†]	Vacc	102/4117	2.48	**41.4**	**1.73**	75/4135	1.81	**42.8**	**1.38**	25/4171	0.60	**39.3**	**0.35**
	Comp	172/3893	4.42	(23.2, 55.2)	(0.64, 2.81)	130/3919	3.32	(22.1, 57.9)	(0.42, 2.35)	40/3976	1.01	(3.2, 61.9)	(-0.09, 0.79)
Total ATP 2	Vacc	95/3249*	2.92	**71.2**	**19.65**	70/3267*	2.14	**69.5**	**16.95**	22/3302*	0.67	**54.5**	**3.13**
	Comp	21/193	10.85	(49.6, 83.5)	(5.60, 33.70)	15/200	7.50	(38.9, 84.8)	(2.89, 31.00)	3/209	1.44	(-129.9, 91.0)	(-3.17, 9.43)
Total ATP 3	Vacc	95/3249	2.92	**34.2**	**1.87**	70/3267	2.14	**35.8**	**1.48**	22/3302	0.67	**28.9**	**0.28**
	Comp	194/4131	4.70	(13.3, 50.1)	(0.57, 3.16)	148/4165	3.55	(11.2, 53.6)	(0.31, 2.65)	41/4242	0.97	(-17.7, 57.1)	(-0.22, 0.79)
Indirect 1[†]	Vacc	105/3454	3.04	**-1.2**	**2.62**	87/3463	2.51	**-5.7**	**2.37**	32/3506	0.91	**15.8**	**2.86**
	Comp	96/3156	3.04	(-43.9, 28.8)	(-3.57, 8.82)	77/3171	2.43	(-58.6, 29.6)	(-3.82, 8.57)	35/3202	1.09	(-83.6, 61.4)	(-3.31, 9.04)
Indirect 2[†]	Vacc	35/1028	3.40	**9.2**	**1.86**	28/1034	2.71	**0.9**	**1.33**	16/1042	1.54	**-144.3**	**-0.35**
	Comp	41/1074	3.82	(-40.8, 41.5)	(-0.42, 4.13)	30/1082	2.77	(-68.6, 41.8)	(-0.94, 3.59)	7/1099	0.64	(-503.0, 1.0)	(-1.57, 0.87)

N, number of cases; PT, person-time at-risk; IR, incidence rate per 100 person-years; VE, vaccine effectiveness; Vacc, Rotarix village; Comp, non-Rotarix villages; RD, rate difference = IR in Comp. – IR in Vacc.

* A number of villages did not contribute person-time. Please refer to Table 19 for more details.

† Primary version(s) of each effect parameter.

Table 2. Direct effectiveness (%, 95% confidence limits [95% CL]) of the human rotavirus vaccine (Rotarix®) and other effects (i.e., selection effects) against acute rotavirus diarrhea, severe ARD, and very severe ARD among children 6-weeks to 2 years-old in Matlab, Bangladesh, 2008-2011.

Effect	Group	Acute Rotavirus Diarrhea (ARD)				Severe ARD (Vesikari >/=11)				Very Severe ARD (Vesikari >/=15)			
		**N / PT**	**IR**	**VE (95% CL)**	**RD (95% CL)**	**N / PT**	**IR**	**VE (95% CL)**	**RD (95% CL)**	**N / PT**	**IR**	**VE (95% CL)**	**RD (95% CL)**
Direct mITT 1	Vacc	108/4735	2.28	**52.3**	**2.31**	80/4754	1.68	**56.5**	**1.51**	25/4790	0.52	**62.3**	**0.68**
	Comp	54/1128	4.79	(32.4, 66.4)	(0.42, 4.20)	44/1136	3.87	(38.1, 69.4)	(-0.03, 3.05)	16/1155	1.39	(32.0, 79.1)	(-0.08, 1.45)
Direct mITT 2	Vacc	112/4951	2.26	**66.6**	**7.18**	82/4970	1.65	**73.2**	**7.43**	26/5006	0.52	**84.1**	**1.29**
	Comp	12/178	6.73	(36.3, 82.5)	(0.56, 13.80)	11/180	6.12	(49.9, 85.6)	(0.82, 14.03)	6/183	3.27	(65.6, 92.6)	(-0.06, 2.65)
Direct mITT 3	Vacc	112/4951	2.26	**46.6**	**1.97**	82/4970	1.65	**53.1**	**1.67**	26/5006	0.52	**65.9**	**0.91**
	Comp	83/1907	4.35	(25.2, 61.8)	(0.63, 3.31)	69/1919	3.59	(33.3, 67.0)	(0.46, 2.88)	30/1946	1.54	(44.1, 79.2)	(0.20, 1.62)
Direct ATP 1	Vacc	102/4117	2.48	**51.7**	**2.45**	75/4135	1.81	**55.9**	**1.56**	25/4171	0.60	**58.5**	**0.67**
	Comp	52/1012	5.14	(30.7, 66.4)	(0.32, 4.59)	42/1020	4.12	(36.3, 69.5)	(-0.18, 3.30)	15/1038	1.44	(23.1, 77.6)	(-0.18, 1.51)
Direct ATP 2	Vacc	95/3249*	2.92	**62.1**	**30.07**	70/3267*	2.14	**68.3**	**30.27**	22/3302*	0.67	**73.2**	**0.92**
	Comp	9/116	7.74	(22.8, 81.4)	(1.50, 58.64)	8/118	6.80	(35.3, 84.5)	(1.69, 58.85)	3/121	2.47	(19.6, 91.1)	(-0.75, 2.59)
Direct ATP 3	Vacc	95/3249*	2.92	**40.6**	**2.27**	70/3267*	2.14	**47.2**	**1.92**	22/3302*	0.67	**60.8**	**0.94**
	Comp	74/1443	5.13	(14.7, 58.6)	(0.41, 4.13)	61/1455	4.19	(22.1, 64.3)	(0.24, 3.61)	26/1481	1.76	(30.9, 77.7)	(0.02, 1.87)
Misc 1†	Vacc	49/879*	5.57	**27.6**	**27.58**	40/887*	4.51	**33.2**	**28.61**	15/905*	1.66	**31.3**	**0.20**
	Comp	9/116	7.74	(-46.1, 64.1)	(-1.57, 56.72)	8/118	6.80	(-39.7, 68.1)	(-0.49, 57.71)	3/121	2.47	(-114.6, 78.0)	(-1.82, 2.23)
Misc 2†	Vacc	198/5230	3.79	**15.1**	**3.58**	145/5259	2.76	**13.6**	**3.40**	44/5321	0.83	**-2.4**	**0.47**
	Comp	37/801	4.62	(-20.3, 40.0)	(-0.39, 7.55)	27/809	3.34	(-26.3, 40.9)	(-0.59, 7.39)	7/825	0.85	(-109.1, 49.8)	(-0.94, 1.89)

N, number of cases; PT, person-time at-risk; IR, incidence rate per 100 person-years; VE, vaccine effectiveness; Vacc, Rotarix recipients; Comp, did not receive Rotarix; RD, rate difference = IR in Comp. – IR in Vacc.

* A number of villages did not contribute person-time. Please refer to Table 19 for more details.

† Since these two parameters assess the level of any differences in risk of rotavirus by oral polio virus vaccine status among those who did not receive Rotarix, the definitions for the comparison groups differ from all other effect parameters (see Figure 6).

Table 3. Overall, total, and indirect effectiveness (%, 95% confidence limits [95% CL]) of the human rotavirus vaccine (Rotarix®) against acute rotavirus diarrhea (ARD) associated with G1 or P8 serotypes or all non-G1 and non-P8 serotypes among children 6-weeks to 2 years-old in Matlab, Bangladesh, 2008-2011.

THE ORIGINAL VERSION OF THIS TABLE WAS REMOVED IN THE FEBRUARY 24, 2016 VERSION OF THIS REPORT. SEE THE NEW VERSION OF TABLE 3 BELOW FOR TOTAL EFFECTIVENESS 1 ESTIMATES FOR ARD BY G AND P SEROTYPES.

Table 3 (revised February 24, 2016). G and P serotype specific estimates of the total effectiveness 1, according to protocol, (%, 95% confidence limits [95% CL]) of the human rotavirus vaccine (Rotarix®) against acute rotavirus diarrhea (ARD) associated with infection by a single strain (i.e., excluding infections with no RT-PCR results; untyped P and/or G serotype; or where more than one G and/or P was detected) among children 6-weeks to 2 years-old in Matlab, Bangladesh, 2008-2011. Table 3.A presents Total ATP 1 estimates by GP combinations for combinations that occurred with sufficient frequency to permit estimation. Table 3.B categorizes all 222 single-strain ARD into two categories G1 or P8 serotypes vs. all non-G1 and non-P8 serotypes.

Table 3.A.

		Acute Rotavirus Diarrhea (ARD)		
**Combination of G and P serotypes**	**Group**	**N / PT**	**IR**	**VE (95% CL)**
G1P8	Vacc	18/4166	0.43	**54.5 (18.7, 74.6)**
	Comp	38/3984	0.95	
G2P4	Vacc	10/4166	0.24	**45.8 (-42.4, 79.4)**
	Comp	17/3988	0.43	
G9P8	Vacc	15/4166	0.36	**20.0 (-44.7, 55.8)**
	Comp	19/3991	0.48	
G12P6	Vacc	10/4167	0.24	**36.4 (-39.3, 71.0)**
	Comp	16/3987	0.40	
G12P8	Vacc	24/4164	0.58	**51.5 (18.5, 71.1)**
	Comp	52/3978	1.31	

N, number of cases; PT, person-time at-risk; IR, incidence rate per 100 person-years; VE, vaccine effectiveness; Vacc, Rotarix village; Comp, non-Rotarix villages

Table 3.B.

		Acute Rotavirus Diarrhea (ARD)		
**Strain Category**	**Group**	**N / PT**	**IR**	**VE (95% CL)**
G1 or P8	Vacc	57/4147	1.37	**48.1 (27.9, 62.7)**
	Comp	111/3950	2.81	
Neither G1 nor P8	Vacc	20/4159	0.48	**43.0 (-2.9, 68.4)**
	Comp	34/3973	0.86	

N, number of cases; PT, person-time at-risk; IR, incidence rate per 100 person-years; VE, vaccine effectiveness; Vacc, Rotarix village; Comp, non-Rotarix villages

Table 4. Direct effectiveness (%, 95% confidence limits [95% CL]) of the human rotavirus vaccine (Rotarix®) and other effects (i.e., selection effects) against acute rotavirus diarrhea (ARD) associated with G1 or P8 serotypes or all non-G1 and non-P8 serotypes among children 6-weeks to 2 years-old in Matlab, Bangladesh, 2008-2011.

THIS TABLE WAS REMOVED IN THE FEBRUARY 24, 2016 VERSION OF THIS REPORT. SEE TABLE 3 FOR TOTAL EFFECTIVENESS 1 ESTIMATES FOR ARD BY G AND P SEROTYPES.

Table 5. Overall, total, and indirect effectiveness (%, 95% confidence limits [95% CL]) of the human rotavirus vaccine (Rotarix®) against acute diarrhea associated with Enterotoxic *E. coli* infection (excluding and including rotavirus co-infected cases) among children 6-weeks to 2 years-old in Matlab, Bangladesh, 2008-2011.

Effect		Acute Enterotoxic *E. coli* Diarrhea, Excluding rotavirus co-positive cases				Acute Enterotoxic *E. coli* Diarrhea, Including rotavirus co-positive cases			
	**Group**	**N / PT**	**IR**	**VE (95% CL)**	**RD (95% CL)**	**N / PT**	**IR**	**VE (95% CL)**	**RD (95% CL)**
Overall 1†	Vacc	29/5944	0.49	**19.2**	**0.14**	54/5928	0.91	**5.6**	**0.14**
	Comp	34/5127	0.66	(-31.4, 50.3)	(-0.20, 0.47)	54/5114	1.06	(-40.7, 36.6)	(-0.35, 0.62)
Overall 2	Vacc	37/7071	0.52	**19.3**	**0.12**	67/7050	0.95	**-0.2**	**0.07**
	Comp	44/6146	0.72	(-25.0, 48.0)	(-0.21, 0.44)	64/6133	1.04	(-44.7, 30.6)	(-0.38, 0.52)
Overall 3	Vacc	30/5594	0.54	**12.5**	**0.11**	54/5579	0.97	**-0.6**	**0.05**
	Comp	32/4713	0.68	(-42.8, 46.3)	(-0.25, 0.46)	50/4701	1.06	(-49.2, 32.1)	(-0.44, 0.53)
Total mITT 1†	Vacc	26/4791	0.54	**5.3**	**0.06**	41/4782	0.86	**4.0**	**0.03**
	Comp	32/5097	0.63	(-55.4, 42.3)	(-0.28, 0.41)	50/5085	0.98	(-46.5, 37.1)	(-0.43, 0.50)
Total mITT 2	Vacc	26/5007	0.52	**38.6**	**0.35**	41/4997	0.82	**27.6**	**1.91**
	Comp	3/333	0.90	(-84.4, 79.6)	(-0.68, 1.38)	4/333	1.20	(-143.9, 78.5)	(-1.68, 5.50)
Total mITT 3	Vacc	26/5007	0.52	**18.9**	**0.11**	41/4997	0.82	**12.3**	**0.11**
	Comp	44/6146	0.72	(-28.6, 48.9)	(-0.22, 0.45)	64/6133	1.04	(-33.5, 42.4)	(-0.36, 0.58)
Total ATP 1†	Vacc	24/4173	0.58	**19.1**	**0.19**	38/4164	0.91	**17.3**	**0.23**
	Comp	31/3982	0.78	(-33.9, 51.2)	(-0.21, 0.59)	48/3970	1.21	(-26.6, 45.9)	(-0.28, 0.73)
Total ATP 2	Vacc	21/3304*	0.64	**-43.8**	**-0.25**	34/3295*	1.03	**-16.3**	**3.15**
	Comp	1/206	0.48	(-722.6, 74.9)	(-0.78, 0.29)	2/206	0.97	(-827.9, 85.4)	(-3.55, 9.85)
Total ATP 3	Vacc	21/3304	0.64	**12.4**	**0.19**	34/3295	1.03	**1.2**	**0.17**
	Comp	35/4242	0.83	(-46.5, 47.6)	(-0.24, 0.63)	50/4230	1.18	(-53.7, 36.5)	(-0.38, 0.72)
Indirect 1†	Vacc	34/3508	0.97	**-46.7**	**-0.29**	49/3494	1.40	**-28.6**	**2.86**
	Comp	23/3213	0.72	(-153.5, 15.2)	(-0.85, 0.27)	37/3202	1.16	(-175.2, 39.9)	(-3.28, 9.01)
Indirect 2†	Vacc	5/1050	0.48	**54.4**	**0.26**	7/1048	0.67	**45.9**	**0.82**
	Comp	12/1090	1.10	(-25.1, 83.4)	(-0.85, 1.37)	14/1089	1.29	(-30.4, 77.5)	(-0.69, 2.34)

N, number of cases; PT, person-time at-risk; IR, incidence rate per 100 person-years; VE, vaccine effectiveness; Vacc, Rotarix village; Comp, non-Rotarix villages; RD, rate difference = IR in Comp. – IR in Vacc.

* A number of villages did not contribute person-time. Please refer to Table 19 for more details.

† Primary version(s) of each effect parameter.

Table 6. Direct effectiveness (%, 95% confidence limits [95% CL]) of the human rotavirus vaccine (Rotarix®) and other effects (i.e., selection effects) against acute diarrhea associated with Enterotoxic *E. coli* infection (excluding and including rotavirus co-infected cases) among children 6-weeks to 2 years-old in Matlab, Bangladesh, 2008-2011.

Effect	Group	Acute Enterotoxic *E. coli* Diarrhea, Excluding rotavirus co-positive cases				Acute Enterotoxic *E. coli* Diarrhea, Including rotavirus co-positive cases			
		N / PT	IR	VE (95% CL)	RD (95% CL)	N / PT	IR	VE (95% CL)	RD (95% CL)
Direct mITT 1	Vacc	26/4791	0.54	**-5.7**	**-0.14**	41/4782	0.86	**47.8**	**0.33**
	Comp	6/1164	0.52	(-78.2, 37.3)	(-0.52, 0.25)	19/1154	1.65	(22.5, 64.8)	(-0.41, 1.08)
Direct mITT 2	Vacc	26/5007	0.52	**68.6**	**1.26**	41/4997	0.82	**50.2**	**0.99**
	Comp	3/184	1.63	(4.1, 89.7)	(-0.54, 3.06)	3/184	1.63	(-50.1, 83.5)	(-0.82, 2.80)
Direct mITT 3	Vacc	26/5007	0.52	**0.8**	**0.00**	41/4997	0.82	**34.8**	0.13
	Comp	11/1962	0.56	(-75.5, 43.9)	(-0.47, 0.47)	26/1950	1.33	(0.7, 57.1)	(-0.44, 0.70)
Direct ATP 1	Vacc	24/4173	0.58	**-1.0**	**-0.10**	38/4164	0.91	**50.0**	**0.50**
	Comp	6/1048	0.57	(-70.9, 40.3)	(-0.52, 0.31)	19/1038	1.83	(25.4, 66.4)	(-0.32, 1.31)
Direct ATP 2	Vacc	21/3304*	0.64	**60.7**	**1.27**	34/3295*	1.03	**36.3**	**0.95**
	Comp	2/122	1.64	(-53.6, 89.9)	(-0.87, 3.41)	2/122	1.64	(-143.3, 83.4)	(-1.21, 3.11)
Direct ATP 3	Vacc	21/3304*	0.64	**-15.3**	**-0.12**	34/3295*	1.03	**28.3**	**0.05**
	Comp	9/1497	0.60	(-110.4, 36.8)	(-0.54, 0.30)	23/1485	1.55	(-9.7, 53.2)	(-0.54, 0.64)
Misc 1†	Vacc	7/913*	0.77	**52.8**	**1.34**	19/903*	2.10	**-29.9**	**0.49**
	Comp	2/122	1.64	(-73.1, 87.1)	(-0.86, 3.53)	2/122	1.64	(-334.3, 61.1)	(-1.91, 2.90)
Misc 2†	Vacc	33/5330	0.62	**50.3**	**-0.01**	52/5317	0.98	**27.6**	**1.27**
	Comp	11/816	1.35	(-6.0, 76.7)	(-0.54, 0.52)	12/816	1.47	(-40.2, 62.6)	(-1.82, 4.37)

N, number of cases; PT, person-time at-risk; IR, incidence rate per 100 person-years; VE, vaccine effectiveness; Vacc, Rotarix recipients; Comp, did not receive Rotarix; RD, rate difference = IR in Comp. – IR in Vacc.
* A number of villages did not contribute person-time. Please refer to Table 19 for more details.
† Since these two parameters assess the level of any differences in risk of rotavirus by oral polio virus vaccine status among those who did not receive Rotarix, the definitions for the comparison groups differ from all other effect parameters (see Figure 6).

Table 7. Overall, total, and indirect effectiveness (%, 95% confidence limits [95% CL]) of the human rotavirus vaccine (Rotarix®) against acute rotavirus diarrhea, severe ARD, and very severe ARD among children 6-weeks to 1 years-old in Matlab, Bangladesh, 2008-2011.

**Effect**	**Group**	**Acute Rotavirus Diarrhea (ARD)**				**Severe ARD (Vesikari >/=11)**				**Very Severe ARD (Vesikari >/=15)**			
		**N / PT**	**IR**	**VE (95% CL)**	**RD (95% CL)**	**N / PT**	**IR**	**VE (95% CL)**	**RD (95% CL)**	**N / PT**	**IR**	**VE (95% CL)**	**RD (95% CL)**
Overall 1†	Vacc	123/3913	3.14	**33.0**	**1.94**	93/3922	2.37	**30.1**	**1.31**	31/3940	0.79	**18.9**	**0.46**
	Comp	166/3411	4.87	(14.6, 47.4)	(0.71, 3.16)	121/3424	3.53	(7.1, 47.4)	(0.22, 2.40)	35/3448	1.02	(-30.4, 49.6)	(-0.09, 1.01)
Overall 2	Vacc	144/4409	3.27	**26.7**	**1.72**	110/4419	2.49	**24.4**	**1.18**	41/4439	0.92	**-2.4**	**0.29**
	Comp	179/3862	4.64	(7.7, 41.8)	(0.55, 2.90)	133/3876	3.43	(0.8, 42.4)	(0.10, 2.25)	37/3902	0.95	(-55.2, 32.4)	(-0.19, 0.78)
Overall 3	Vacc	111/3551	3.13	**33.3**	**1.75**	83/3560	2.33	**31.1**	**1.19**	26/3576	0.73	**27.8**	**0.42**
	Comp	147/3011	4.88	(13.7, 48.5)	(0.47, 3.03)	107/3023	3.54	(7.4, 48.8)	(0.07, 2.32)	32/3045	1.05	(-19.7, 56.5)	(-0.11, 0.95)
Total mITT 1†	Vacc	81/3058	2.65	**42.4**	**2.00**	58/3066	1.89	**42.9**	**1.40**	17/3079	0.55	**45.2**	**0.55**
	Comp	152/3193	4.76	(23.5, 56.5)	(0.79, 3.20)	111/3206	3.46	(21.5, 58.5)	(0.38, 2.43)	34/3227	1.05	(4.8, 68.4)	(0.06, 1.04)
Total mITT 2	Vacc	85/3274	2.60	**65.3**	**8.45**	60/3282	1.83	**67.6**	**6.71**	18/3294	0.55	**38.1**	**1.82**
	Comp	17/223	7.63	(40.7, 79.6)	(2.92, 13.99)	13/225	5.78	(40.4, 82.4)	(1.38, 12.04)	2/228	0.88	(-325.9, 91.0)	(-1.62, 5.26)
Total mITT 3	Vacc	85/3274	2.60	**42.0**	**2.23**	60/3282	1.83	**44.4**	**1.68**	18/3294	0.55	**39.8**	**0.58**
	Comp	179/3862	4.64	(24.0, 55.6)	(1.06, 3.39)	133/3876	3.43	(23.8, 59.4)	(0.65, 2.70)	37/3902	0.95	(-3.6, 65.0)	(0.13, 1.04)
Total ATP 1†	Vacc	75/2464	3.04	**45.2**	**2.61**	53/2472	2.14	**48.0**	**2.08**	17/2484	0.68	**45.9**	**0.72**
	Comp	135/2340	5.77	(26.3, 59.3)	(1.10, 4.12)	101/2351	4.30	(27.0, 63.0)	(0.75, 3.41)	31/2371	1.31	(3.6, 69.6)	(0.07, 1.37)
Total ATP 2	Vacc	68/1597*	4.26	**69.7**	**23.34**	48/1603*	2.99	**72.3**	**18.83**	14/1615*	0.87	**10.7**	**2.81**
	Comp	14/96	14.55	(41.9, 84.2)	(8.16, 38.51)	11/98	11.25	(41.8, 86.8)	(4.40, 33.26)	1/101	0.99	(-1448.3, 94.9)	(-3.50, 9.12)
Total ATP 3	Vacc	68/1597	4.26	**37.1**	**3.46**	48/1603	2.99	**43.1**	**2.90**	14/1615	0.87	**32.6**	**0.76**
	Comp	138/1962	7.03	(13.3, 54.3)	(1.23, 5.69)	109/1973	5.52	(17.1, 61.0)	(0.88, 4.92)	27/1997	1.35	(-27.8, 64.4)	(-0.08, 1.59)
Indirect 1†	Vacc	37/519	7.12	**1.6**	**2.16**	31/520	5.96	**1.6**	**1.20**	13/525	2.48	**29.9**	**7.93**
	Comp	34/482	7.06	(-96.2, 50.7)	(-21.55, 25.86)	29/483	6.00	(-114.6, 54.9)	(-22.43, 24.83)	17/486	3.50	(-127.1, 78.4)	(-11.54, 27.40)
Indirect 2†	Vacc	26/651	3.99	**-6.1**	**2.21**	21/652	3.22	**-4.7**	**1.82**	12/654	1.83	**-341.1**	**-0.17**
	Comp	27/706	3.83	(-78.0, 36.7)	(-0.44, 4.86)	22/708	3.11	(-90.3, 42.4)	(-0.79, 4.43)	3/712	0.42	(-1930.6, 4.2)	(-1.70, 1.36)

N, number of cases; PT, person-time at-risk; IR, incidence rate per 100 person-years; VE, vaccine effectiveness; Vacc, Rotarix village; Comp, non-Rotarix villages; RD, rate difference = IR in Comp. – IR in Vacc.

* A number of villages did not contribute person-time. Please refer to Table 19 for more details.

† Primary version(s) of each effect parameter.

Table 8. Direct effectiveness (%, 95% confidence limits [95% CL]) of the human rotavirus vaccine (Rotarix®) and other effects (i.e., selection effects) against acute rotavirus diarrhea, severe ARD, and very severe ARD among children 6-weeks to 1 years-old in Matlab, Bangladesh, 2008-2011.

Effect	Group	Acute Rotavirus Diarrhea (ARD)				Severe ARD (Vesikari >/=11)				Very Severe ARD (Vesikari >/=15)			
		N / PT	IR	VE (95% CL)	RD (95% CL)	N / PT	IR	VE (95% CL)	RD (95% CL)	N / PT	IR	VE (95% CL)	RD (95% CL)
Direct mITT 1	Vacc	81/3058	2.65	**55.2**	**1.98**	58/3066	1.89	**60.9**	**1.67**	17/3079	0.55	**71.6**	**0.93**
	Comp	39/664	5.88	(32.8, 70.1)	(0.09, 3.86)	32/665	4.81	(41.0, 74.0)	(-0.01, 3.36)	13/670	1.94	(38.7, 86.9)	(-0.01, 1.87)
Direct mITT 2	Vacc	85/3274	2.60	**72.3**	**7.45**	60/3282	1.83	**78.5**	**7.73**	18/3294	0.55	**86.6**	**1.59**
	Comp	11/119	9.25	(46.8, 85.6)	(0.66, 14.24)	10/119	8.37	(59.4, 88.6)	(0.98, 14.47)	5/121	4.14	(67.7, 94.5)	(-0.26, 3.44)
Direct mITT 3	Vacc	85/3274	2.60	**52.2**	**1.84**	60/3282	1.83	**60.3**	**1.89**	18/3294	0.55	**74.1**	**1.03**
	Comp	59/1071	5.51	(28.0, 68.3)	(0.30, 3.38)	50/1073	4.66	(40.8, 73.4)	(0.46, 3.33)	23/1081	2.13	(49.8, 86.6)	(0.23, 1.83)
Direct ATP 1	Vacc	75/2464	3.04	**55.2**	**2.12**	53/2472	2.14	**60.9**	**1.76**	17/2484	0.68	**68.6**	**0.95**
	Comp	37/547	6.76	(31.4, 70.8)	(-0.12, 4.36)	30/549	5.46	(39.2, 74.9)	(-0.23, 3.74)	12/554	2.17	(29.3, 86.0)	(-0.15, 2.05)
Direct ATP 2	Vacc	68/1597*	4.26	**70.1**	**29.75**	48/1603*	2.99	**75.7**	**29.96**	14/1615*	0.87	**74.5**	**0.44**
	Comp	8/57	14.06	(38.6, 85.4)	(1.07, 58.42)	7/57	12.21	(50.0, 88.2)	(1.29, 58.63)	2/59	3.41	(10.1, 92.8)	(-1.24, 2.11)
Direct ATP 3	Vacc	68/1597*	4.26	**47.1**	**2.38**	48/1603*	2.99	**55.7**	**2.52**	14/1615*	0.87	**71.2**	**1.21**
	Comp	50/607	8.24	(15.5, 66.9)	(-0.28, 5.05)	42/609	6.90	(29.0, 72.4)	(0.09, 4.95)	19/616	3.09	(38.9, 86.4)	(0.01, 2.41)
Misc 1†	Vacc	34/390*	8.72	**40.0**	**27.69**	28/391*	7.15	**42.8**	**28.13**	12/396*	3.03	**13.5**	**-0.77**
	Comp	8/57	14.06	(-21.5, 70.4)	(-1.60, 56.98)	7/57	12.21	(-22.6, 73.3)	(-1.11, 57.37)	2/59	3.41	(-253.3, 78.8)	(-3.20, 1.65)
Misc 2†	Vacc	156/3425	4.55	**12.9**	**4.03**	114/3438	3.32	**22.8**	**4.25**	34/3460	0.98	**-47.2**	**0.74**
	Comp	23/436	5.27	(-34.4, 43.6)	(-0.51, 8.57)	19/438	4.34	(-23.4, 51.7)	(-0.27, 8.77)	3/442	0.68	(-396.0, 56.3)	(-1.47, 2.94)

N, number of cases; PT, person-time at-risk; IR, incidence rate per 100 person-years; VE, vaccine effectiveness; Vacc, Rotarix recipients; Comp, did not receive Rotarix; RD, rate difference = IR in Comp. – IR in Vacc.

* A number of villages did not contribute person-time. Please refer to Table 19 for more details.

† Since these two parameters assess the level of any differences in risk of rotavirus by oral polio virus vaccine status among those who did not receive Rotarix, the definitions for the comparison groups differ from all other effect parameters (see Figure 6).

Table 9. Overall, total, and indirect effectiveness (%, 95% confidence limits [95% CL]) of the human rotavirus vaccine (Rotarix®) against acute rotavirus diarrhea (ARD) associated with G1 or P8 serotypes or all non-G1 and non-P8 serotypes among children 6-weeks to 1 years-old in Matlab, Bangladesh, 2008-2011.

THIS TABLE WAS REMOVED IN THE FEBRUARY 24, 2016 VERSION OF THIS REPORT. SEE TABLE 3 FOR TOTAL EFFECTIVENESS 1 ESTIMATES FOR ARD BY G AND P SEROTYPES.

Table 10. Direct effectiveness (%, 95% confidence limits [95% CL]) of the human rotavirus vaccine (Rotarix®) and other effects (i.e., selection effects) against acute rotavirus diarrhea (ARD) associated with G1 or P8 serotypes or all non-G1 and non-P8 serotypes among children 6-weeks to 1 years-old in Matlab, Bangladesh, 2008-2011.

THIS TABLE WAS REMOVED IN THE FEBRUARY 24, 2016 VERSION OF THIS REPORT. SEE TABLE 3 FOR TOTAL EFFECTIVENESS 1 ESTIMATES FOR ARD BY G AND P SEROTYPES.

Table 11. Overall, total, and indirect effectiveness (%, 95% confidence limits [95% CL]) of the human rotavirus vaccine (Rotarix®) against acute diarrhea associated with Enterotoxic *E. coli* infection (excluding and including rotavirus co-infected cases) among children 6-weeks to 1 years-old in Matlab, Bangladesh, 2008-2011.

**Effect**		**Acute Enterotoxic *E. coli* Diarrhea, Excluding rotavirus co-positive cases**				**Acute Enterotoxic *E. coli* Diarrhea, Including rotavirus co-positive cases**			
	**Group**	**N / PT**	**IR**	**VE (95% CL)**	**RD (95% CL)**	**N / PT**	**IR**	**VE (95% CL)**	**RD (95% CL)**
Overall 1†	Vacc	19/3942	0.48	**21.5**	**0.29**	37/3938	0.94	**13.9**	**0.30**
	Comp	24/3449	0.70	(-37.6, 55.2)	(-0.11, 0.68)	42/3444	1.22	(-34.7, 44.9)	(-0.31, 0.92)
Overall 2	Vacc	25/4442	0.56	**24.4**	**0.27**	47/4437	1.06	**9.2**	**0.24**
	Comp	33/3900	0.85	(-21.5, 53.0)	(-0.15, 0.70)	51/3895	1.31	(-35.2, 39.0)	(-0.38, 0.85)
Overall 3	Vacc	20/3578	0.56	**15.0**	**0.26**	38/3574	1.06	**6.0**	**0.18**
	Comp	23/3046	0.76	(-46.7, 50.8)	(-0.18, 0.70)	39/3041	1.28	(-46.6, 39.7)	(-0.45, 0.82)
Total mITT 1†	Vacc	17/3078	0.55	**13.0**	**0.24**	29/3076	0.94	**13.4**	**0.21**
	Comp	23/3229	0.71	(-51.2, 50.0)	(-0.17, 0.66)	39/3224	1.21	(-39.2, 46.1)	(-0.39, 0.81)
Total mITT 2	Vacc	17/3294	0.52	**59.3**	**0.58**	29/3292	0.88	**48.6**	**2.09**
	Comp	3/226	1.33	(-14.4, 85.5)	(-0.60, 1.76)	4/226	1.77	(-53.3, 82.8)	(-1.55, 5.73)
Total mITT 3	Vacc	17/3294	0.52	**31.0**	**0.34**	29/3292	0.88	**24.9**	**0.36**
	Comp	33/3900	0.85	(-14.8, 58.6)	(-0.07, 0.75)	51/3895	1.31	(-18.8, 52.5)	(-0.26, 0.98)
Total ATP 1†	Vacc	15/2484	0.60	**26.7**	**0.47**	26/2482	1.05	**25.7**	**0.51**
	Comp	22/2373	0.93	(-30.3, 58.7)	(-0.04, 0.98)	37/2368	1.56	(-19.8, 53.9)	(-0.16, 1.18)
Total ATP 2	Vacc	12/1615*	0.74	**19.6**	**0.08**	22/1613*	1.36	**28.5**	**3.34**
	Comp	1/99	1.01	(-310.0, 84.2)	(-0.80, 0.96)	2/99	2.02	(-269.2, 86.2)	(-3.41, 10.09)
Total ATP 3	Vacc	12/1615	0.74	**28.4**	**0.72**	22/1613	1.36	**16.9**	**0.75**
	Comp	24/1997	1.20	(-24.9, 58.9)	(0.08, 1.35)	37/1992	1.86	(-38.2, 50.0)	(-0.15, 1.65)
Indirect 1†	Vacc	11/525	2.10	**-35.3**	**-0.51**	19/523	3.63	**-29.8**	**8.60**
	Comp	8/488	1.64	(-224.8, 43.7)	(-2.06, 1.04)	14/486	2.88	(-315.6, 59.4)	(-10.29, 27.50)
Indirect 2†	Vacc	4/655	0.61	**53.3**	**0.30**	5/655	0.76	**52.3**	**0.92**
	Comp	10/708	1.41	(-48.1, 85.3)	(-1.36, 1.96)	12/708	1.69	(-36.4, 83.3)	(-1.03, 2.87)

N, number of cases; PT, person-time at-risk; IR, incidence rate per 100 person-years; VE, vaccine effectiveness; Vacc, Rotarix village; Comp, non-Rotarix villages; RD, rate difference = IR in Comp. – IR in Vacc.

* A number of villages did not contribute person-time. Please refer to Table 19 for more details.

† Primary version(s) of each effect parameter.

Table 12. Direct effectiveness (%, 95% confidence limits [95% CL]) of the human rotavirus vaccine (Rotarix®) and other effects (i.e., selection effects) against acute diarrhea associated with Enterotoxic *E. coli* infection (excluding and including rotavirus co-infected cases) among children 6-weeks to 1 years-old in Matlab, Bangladesh, 2008-2011.

Effect	Group	Acute Enterotoxic *E. coli* Diarrhea, Excluding rotavirus co-positive cases				Acute Enterotoxic *E. coli* Diarrhea, Including rotavirus co-positive cases			
		**N / PT**	**IR**	**VE (95% CL)**	**RD (95% CL)**	**N / PT**	**IR**	**VE (95% CL)**	**RD (95% CL)**
Direct mITT 1	Vacc	17/3078	0.55	**8.3**	**-0.03**	29/3076	0.94	**52.0**	**0.60**
	Comp	4/673	0.59	(-96.7, 57.2)	(-0.50, 0.44)	13/671	1.94	(15.1, 72.8)	(-0.41, 1.61)
Direct mITT 2	Vacc	17/3294	0.52	**71.1**	**1.44**	29/3292	0.88	**49.2**	**1.11**
	Comp	2/121	1.66	(-19.9, 93.0)	(-1.11, 3.99)	2/121	1.66	(-100.5, 87.1)	(-1.46, 3.68)
Direct mITT 3	Vacc	17/3294	0.52	**26.2**	**0.26**	29/3292	0.88	**44.9**	**0.47**
	Comp	8/1084	0.74	(-55.9, 65.1)	(-0.46, 0.98)	18/1081	1.66	(9.0, 66.6)	(-0.40, 1.34)
Direct ATP 1	Vacc	15/2484	0.60	**17.1**	**0.06**	26/2482	1.05	**55.9**	**0.92**
	Comp	4/556	0.72	(-68.8, 59.2)	(-0.47, 0.59)	13/554	2.35	(24.1, 74.3)	(-0.26, 2.09)
Direct ATP 2	Vacc	12/1615*	0.74	**58.7**	**0.19**	22/1613*	1.36	**22.3**	**-0.29**
	Comp	1/59	1.70	(-167.7, 93.6)	(-0.64, 1.02)	1/59	1.70	(-382.1, 87.5)	(-1.29, 0.70)
Direct ATP 3	Vacc	12/1615*	0.74	**16.5**	**0.18**	22/1613*	1.36	**40.5**	**0.56**
	Comp	6/619	0.97	(-74.1, 59.9)	(-0.39, 0.75)	15/616	2.43	(-0.4, 64.7)	(-0.50, 1.62)
Misc 1†	Vacc	5/398*	1.26	**30.8**	**-0.09**	13/396*	3.28	**-81.0**	**-1.41**
	Comp	1/59	1.70	(-337.9, 89.1)	(-1.26, 1.08)	1/59	1.70	(-741.1, 61.0)	(-3.60, 0.78)
Misc 2†	Vacc	24/3462	0.69	**65.4**	**0.19**	41/3457	1.19	**46.7**	**1.42**
	Comp	9/439	2.05	(14.9, 86.0)	(-0.67, 1.06)	10/439	2.28	(-14.1, 75.1)	(-1.74, 4.59)

N, number of cases; PT, person-time at-risk; IR, incidence rate per 100 person-years; VE, vaccine effectiveness; Vacc, Rotarix recipients; Comp, did not receive Rotarix; RD, rate difference = IR in Comp. – IR in Vacc.
* A number of villages did not contribute person-time. Please refer to Table 19 for more details.
† Since these two parameters assess the level of any differences in risk of rotavirus by oral polio virus vaccine status among those who did not receive Rotarix, the definitions for the comparison groups differ from all other effect parameters (see Figure 6).

Table 13. Overall, total, and indirect effectiveness (%, 95% confidence limits [95% CL]) of the human rotavirus vaccine (Rotarix®) against acute rotavirus diarrhea, severe ARD, and very severe ARD among children 1 to 2 years old in Matlab, Bangladesh, 2008-2011.

Effect	Group	Acute Rotavirus Diarrhea (ARD)				Severe ARD (Vesikari >/=11)				Very Severe ARD (Vesikari >/=15)			
		N / PT	IR	VE (95% CL)	RD (95% CL)	N / PT	IR	VE (95% CL)	RD (95% CL)	N / PT	IR	VE (95% CL)	RD (95% CL)
Overall 1†	Vacc	41/1944*	2.11	**12.2**	**-0.21**	35/1958*	1.79	**-7.7**	**-0.21**	12/1993*	0.60	**-10.4**	**-0.57**
	Comp	40/1615	2.48	(-37.4, 43.9)	(-1.53, 1.11)	28/1633	1.71	(-81.8, 36.2)	(-1.41, 0.99)	9/1673	0.54	(-155.8, 52.4)	(-1.18, 0.05)
Overall 2	Vacc	51/2552*	2.00	**19.0**	**-0.03**	41/2573*	1.59	**6.6**	**-0.10**	15/2615*	0.57	**7.1**	**-0.34**
	Comp	56/2169	2.58	(-17.8, 44.3)	(-1.09, 1.03)	39/2192	1.78	(-45.9, 40.2)	(-1.03, 0.83)	14/2244	0.62	(-96.9, 56.2)	(-0.94, 0.25)
Overall 3	Vacc	39/1961*	1.99	**16.2**	**-0.02**	33/1976*	1.67	**4.5**	**0.08**	11/2010*	0.55	**2.1**	**-0.31**
	Comp	39/1609	2.42	(-32.8, 47.1)	(-1.30, 1.27)	29/1624	1.79	(-62.2, 43.8)	(-1.09, 1.26)	9/1663	0.54	(-128.0, 58.0)	(-0.89, 0.27)
Total mITT 1†	Vacc	27/1677*	1.61	**27.9**	**0.40**	22/1688*	1.30	**19.7**	**0.38**	8/1712*	0.47	**15.4**	**-0.14**
	Comp	42/1805	2.33	(-15.6, 55.1)	(-0.78, 1.58)	31/1821	1.70	(-39.2, 53.6)	(-0.71, 1.48)	10/1861	0.54	(-107.0, 65.5)	(-0.75, 0.47)
Total mITT 2	Vacc	27/1677*	1.61	**75.5**	**2.26**	22/1688*	1.30	**64.6**	**0.62**	8/1712*	0.47	**79.2**	**0.03**
	Comp	7/97	7.19	(48.6, 88.3)	(-0.23, 4.76)	4/102	3.92	(9.1, 86.2)	(-1.06, 2.31)	2/108	1.85	(15.6, 94.9)	(-0.88, 0.95)
Total mITT 3	Vacc	27/1677*	1.61	**33.4**	**0.47**	22/1688*	1.30	**21.8**	**0.23**	8/1712*	0.47	**27.7**	**-0.16**
	Comp	56/2169	2.58	(-2.5, 56.7)	(-0.62, 1.55)	39/2192	1.78	(-28.0, 52.2)	(-0.71, 1.17)	14/2244	0.62	(-62.2, 67.8)	(-0.74, 0.42)
Total ATP 1†	Vacc	27/1652*	1.63	**28.9**	**0.36**	22/1664*	1.32	**25.8**	**0.36**	8/1687*	0.47	**18.8**	**-0.24**
	Comp	37/1553	2.38	(-15.6, 56.3)	(-0.89, 1.62)	29/1568	1.85	(-29.5, 57.5)	(-0.80, 1.52)	9/1605	0.56	(-92.3, 65.7)	(-0.82, 0.34)
Total ATP 2	Vacc	27/1652*	1.63	**75.2**	**2.22**	22/1664*	1.32	**64.2**	**0.58**	8/1687*	0.47	**78.9**	**0.03**
	Comp	7/97	7.19	(47.6, 88.3)	(-0.29, 4.73)	4/102	3.92	(7.0, 86.2)	(-1.13, 2.29)	2/108	1.85	(14.5, 94.8)	(-0.88, 0.95)
Total ATP 3	Vacc	27/1652*	1.63	**32.5**	**0.42**	22/1664*	1.32	**20.8**	**0.19**	8/1687*	0.47	**26.6**	**-0.16**
	Comp	56/2169	2.58	(-4.2, 56.3)	(-0.69, 1.53)	39/2192	1.78	(-30.2, 51.8)	(-0.78, 1.16)	14/2244	0.62	(-64.2, 67.2)	(-0.74, 0.42)
Indirect 1†	Vacc	68/2934	2.32	**-3.4**	**0.24**	56/2943	1.90	**-10.6**	**-0.02**	19/2981	0.64	**1.8**	**0.17**
	Comp	62/2675	2.32	(-44.5, 26.0)	(-0.79, 1.28)	48/2688	1.79	(-58.1, 22.6)	(-0.79, 0.75)	18/2716	0.66	(-73.4, 44.4)	(-0.29, 0.63)
Indirect 2†	Vacc	9/377*	2.39	**35.7**	**-0.20**	7/382*	1.83	**11.6**	**-1.03**	4/387*	1.03	**-1.2**	**-0.88**
	Comp	14/368	3.80	(-37.4, 70.0)	(-2.68, 2.28)	8/374	2.14	(-117.6, 64.1)	(-3.21, 1.14)	4/386	1.04	(-248.4, 70.6)	(-2.53, 0.76)

N, number of cases; PT, person-time at-risk; IR, incidence rate per 100 person-years; VE, vaccine effectiveness; Vacc, Rotarix village; Comp, non-Rotarix villages; RD, rate difference = IR in Comp. – IR in Vacc.

* A number of villages did not contribute person-time. Please refer to Table 19 for more details.

† Primary version(s) of each effect parameter.

Table 14. Direct effectiveness (%, 95% confidence limits [95% CL]) of the human rotavirus vaccine (Rotarix®) and other effects (i.e., selection effects) against acute rotavirus diarrhea, severe ARD, and very severe ARD among children 1 to 2 years old in Matlab, Bangladesh, 2008-2011.

Effect	Group	Acute Rotavirus Diarrhea (ARD)				Severe ARD (Vesikari >/=11)				Very Severe ARD (Vesikari >/=15)			
		**N / PT**	**IR**	**VE (95% CL)**	**RD (95% CL)**	**N / PT**	**IR**	**VE (95% CL)**	**RD (95% CL)**	**N / PT**	**IR**	**VE (95% CL)**	**RD (95% CL)**
Direct mITT 1	Vacc	27/1677*	1.61	**49.8**	**4.52**	22/1688*	1.30	**49.0**	**2.97**	8/1712*	0.47	**26.2**	**0.55**
	Comp	15/465	3.23	(5.4, 73.3)	(-1.37, 10.41)	12/471	2.55	(-0.6, 74.1)	(-2.26, 8.20)	3/485	0.62	(-147.3, 78.0)	(-1.11, 2.20)
Direct mITT 2	Vacc	27/1677*	1.61	**1.6**	**1.08**	22/1688*	1.30	**19.7**	**1.39**	8/1712*	0.47	**72.0**	**1.74**
	Comp	1/59	1.69	(-658.9, 87.3)	(-2.40, 4.55)	1/60	1.66	(-522.5, 89.6)	(-2.04, 4.82)	1/63	1.60	(-110.0, 96.3)	(-1.47, 4.96)
Direct mITT 3	Vacc	27/1677*	1.61	**41.2**	**2.75**	22/1688*	1.30	**40.2**	**2.00**	8/1712*	0.47	**42.0**	**0.88**
	Comp	24/836	2.87	(-8.6, 68.1)	(-0.37, 5.86)	19/846	2.25	(-16.2, 69.3)	(-0.81, 4.81)	7/865	0.81	(-53.1, 78.0)	(-0.59, 2.35)
Direct ATP 1	Vacc	27/1652*	1.63	**49.1**	**4.48**	22/1664*	1.32	**48.2**	**2.93**	8/1687*	0.47	**25.1**	**0.55**
	Comp	15/465	3.23	(3.8, 73.0)	(-1.42, 10.37)	12/471	2.55	(-2.2, 73.8)	(-2.30, 8.16)	3/485	0.62	(-150.0, 77.5)	(-1.11, 2.20)
Direct ATP 2	Vacc	27/1652*	1.63	**0.4**	**1.03**	22/1664*	1.32	**18.7**	**1.34**	8/1687*	0.47	**71.6**	**1.74**
	Comp	1/59	1.69	(-669.9, 87.1)	(-2.46, 4.52)	1/60	1.66	(-531.5, 89.5)	(-2.11, 4.79)	1/63	1.60	(-113.5, 96.2)	(-1.47, 4.96)
Direct ATP 3	Vacc	27/1652*	1.63	**40.4**	**2.70**	22/1664*	1.32	**39.5**	**1.96**	8/1687*	0.47	**41.2**	**0.88**
	Comp	24/836	2.87	(-10.3, 67.8)	(-0.42, 5.83)	19/846	2.25	(-17.9, 68.9)	(-0.86, 4.78)	7/865	0.81	(-55.0, 77.7)	(-0.59, 2.35)
Misc 1[†]	Vacc	15/489*	3.07	**-82.8**	**-3.44**	12/495*	2.42	**-44.7**	**-1.80**	3/509*	0.59	**61.7**	**1.21**
	Comp	1/59	1.69	(-1300.7, 76.1)	(-12.31, 5.43)	1/60	1.66	(-1045.5, 81.7)	(-9.80, 6.21)	1/63	1.60	(-241.9, 95.7)	(-2.58, 5.01)
Misc 2[†]	Vacc	42/1805*	2.33	**34.5**	**0.43**	31/1821*	1.70	**12.4**	**-0.49**	10/1861*	0.54	**48.5**	**0.06**
	Comp	14/365	3.84	(-12.4, 61.9)	(-1.11, 1.97)	8/371	2.16	(-73.0, 55.7)	(-1.67, 0.70)	4/383	1.04	(-49.6, 82.2)	(-0.60, 0.73)

N, number of cases; PT, person-time at-risk; IR, incidence rate per 100 person-years; VE, vaccine effectiveness; Vacc, Rotarix recipients; Comp, did not receive Rotarix; RD, rate difference = IR in Comp. – IR in Vacc.

* A number of villages did not contribute person-time. Please refer to Table 19 for more details.

† Since these two parameters assess the level of any differences in risk of rotavirus by oral polio virus vaccine status among those who did not receive Rotarix, the definitions for the comparison groups differ from all other effect parameters (see Figure 6).

Table 15. Overall, total, and indirect effectiveness (%, 95% confidence limits [95% CL]) of the human rotavirus vaccine (Rotarix®) against acute rotavirus diarrhea (ARD) associated with G1 or P8 serotypes or all non-G1 and non-P8 serotypes among children 1 to 2 years old in Matlab, Bangladesh, 2008-2011.

THIS TABLE WAS REMOVED IN THE FEBRUARY 24, 2016 VERSION OF THIS REPORT. SEE TABLE 3 FOR TOTAL EFFECTIVENESS 1 ESTIMATES FOR ARD BY G AND P SEROTYPES.

Table 16. Direct effectiveness (%, 95% confidence limits [95% CL]) of the human rotavirus vaccine (Rotarix®) and other effects (i.e., selection effects) against acute rotavirus diarrhea (ARD) associated with G1 or P8 serotypes or all non-G1 and non-P8 serotypes among children 1 to 2 years old in Matlab, Bangladesh, 2008-2011.

THIS TABLE WAS REMOVED IN THE FEBRUARY 24, 2016 VERSION OF THIS REPORT. SEE TABLE 3 FOR TOTAL EFFECTIVENESS 1 ESTIMATES FOR ARD BY G AND P SEROTYPES.

Table 17. Overall, total, and indirect effectiveness (%, 95% confidence limits [95% CL]) of the human rotavirus vaccine (Rotarix®) against acute diarrhea associated with Enterotoxic *E. coli* infection (excluding and including rotavirus co-infected cases) among children 1 to 2 years old in Matlab, Bangladesh, 2008-2011.

Effect		Acute Enterotoxic *E. coli* Diarrhea, Excluding rotavirus co-positive cases				Acute Enterotoxic *E. coli* Diarrhea, Including rotavirus co-positive cases			
	**Group**	**N / PT**	**IR**	**VE (95% CL)**	**RD (95% CL)**	**N / PT**	**IR**	**VE (95% CL)**	**RD (95% CL)**
Overall 1†	Vacc	10/2002*	0.50	**13.8**	**-0.21**	17/1990*	0.85	**-22.6**	**-0.26**
	Comp	10/1678	0.60	(-108.0, 64.2)	(-0.77, 0.35)	12/1670	0.72	(-149.2, 39.7)	(-0.92, 0.40)
Overall 2	Vacc	12/2630*	0.46	**4.7**	**-0.17**	20/2613*	0.77	**-35.7**	**-0.24**
	Comp	11/2246	0.49	(-123.1, 59.3)	(-0.58, 0.24)	13/2238	0.58	(-168.3, 31.3)	(-0.71, 0.22)
Overall 3	Vacc	10/2016*	0.50	**6.2**	**-0.21**	16/2005*	0.80	**-22.6**	**-0.23**
	Comp	9/1667	0.54	(-129.7, 61.7)	(-0.75, 0.33)	11/1660	0.66	(-157.8, 41.7)	(-0.87, 0.41)
Total mITT 1†	Vacc	9/1713*	0.53	**-12.5**	**-0.29**	12/1706*	0.70	**-25.0**	**-0.31**
	Comp	9/1868	0.48	(-184.9, 55.6)	(-0.85, 0.28)	11/1861	0.59	(-184.4, 45.1)	(-0.96, 0.35)
Total mITT 2	Vacc								
	Comp								
Total mITT 3	Vacc	9/1713*	0.53	**-13.8**	**-0.30**	12/1706*	0.70	**-30.5**	**-0.38**
	Comp	11/2246	0.49	(-184.1, 54.4)	(-0.86, 0.25)	13/2238	0.58	(-189.5, 41.2)	(-1.00, 0.24)
Total ATP 1†	Vacc	9/1689*	0.53	**1.9**	**-0.25**	12/1682*	0.71	**-8.7**	**-0.26**
	Comp	9/1609	0.56	(-145.7, 60.9)	(-0.83, 0.33)	11/1602	0.69	(-144.6, 51.7)	(-0.94, 0.42)
Total ATP 2	Vacc								
	Comp								
Total ATP 3	Vacc	9/1689*	0.53	**-15.2**	**-0.31**	12/1682*	0.71	**-32.1**	**-0.39**
	Comp	11/2246	0.49	(-187.3, 53.8)	(-0.87, 0.25)	13/2238	0.58	(-192.8, 40.5)	(-1.01, 0.23)
Indirect 1†	Vacc	23/2983	0.77	**-52.7**	**-0.24**	30/2971	1.01	**-28.0**	**-0.09**
	Comp	15/2725	0.55	(-199.6, 22.2)	(-0.83, 0.35)	23/2715	0.85	(-127.5, 28.0)	(-0.80, 0.62)
Indirect 2†	Vacc	1/395*	0.25	**50.1**	**0.02**	2/393*	0.51	**-0.1**	**-0.07**
	Comp	2/381	0.52	(-76.9, 85.9)	(-0.33, 0.36)	2/381	0.53	(-168.8, 62.8)	(-0.45, 0.32)

N, number of cases; PT, person-time at-risk; IR, incidence rate per 100 person-years; VE, vaccine effectiveness; Vacc, Rotarix village; Comp, non-Rotarix villages; RD, rate difference = IR in Comp. – IR in Vacc.

* A number of villages did not contribute person-time. Please refer to Table 19 for more details.

† Primary version(s) of each effect parameter.

Table 18. Direct effectiveness (%, 95% confidence limits [95% CL]) of the human rotavirus vaccine (Rotarix®) and other effects (i.e., selection effects) against acute diarrhea associated with Enterotoxic *E. coli* infection (excluding and including rotavirus co-infected cases) among children 1 to 2 years old in Matlab, Bangladesh, 2008-2011.

**Effect**		**Acute Enterotoxic *E. coli* Diarrhea, Excluding rotavirus co-positive cases**				**Acute Enterotoxic *E. coli* Diarrhea, Including rotavirus co-positive cases**			
	**Group**	**N / PT**	**IR**	**VE (95% CL)**	**RD (95% CL)**	**N / PT**	**IR**	**VE (95% CL)**	**RD (95% CL)**
Direct mITT 1	Vacc	9/1713*	0.53	**-27.6**	**-0.35**	12/1706*	0.70	**43.3**	**-0.21**
	Comp	2/491	0.41	(-299.9, 59.3)	(-0.96, 0.26)	6/484	1.24	(-35.0, 76.2)	(-0.98, 0.56)
Direct mITT 2	Vacc	9/1713*	0.53	**65.9**	**5.74**	12/1706*	0.70	**53.5**	**5.56**
	Comp	1/64	1.57	(-175.9, 95.8)	(-2.74, 14.22)	1/64	1.57	(-270.2, 94.2)	(-2.93, 14.05)
Direct mITT 3	Vacc	9/1713*	0.53	**-51.2**	**-0.34**	12/1706*	0.70	**22.7**	**-0.31**
	Comp	3/878	0.34	(-330.9, 47.0)	(-0.92, 0.24)	8/869	0.92	(-86.0, 67.9)	(-0.98, 0.36)
Direct ATP 1	Vacc	9/1689*	0.53	**-29.4**	**-0.35**	12/1682*	0.71	**42.5**	**-0.21**
	Comp	2/491	0.41	(-302.3, 58.4)	(-0.97, 0.26)	6/484	1.24	(-36.3, 75.8)	(-0.99, 0.56)
Direct ATP 2	Vacc	9/1689*	0.53	**65.5**	**5.74**	12/1682*	0.71	**52.9**	**5.55**
	Comp	1/64	1.57	(-179.9, 95.7)	(-2.75, 14.22)	1/64	1.57	(-275.0, 94.1)	(-2.94, 14.04)
Direct ATP 3	Vacc	9/1689*	0.53	**-53.1**	**-0.35**	12/1682*	0.71	**21.7**	**-0.32**
	Comp	3/878	0.34	(-334.4, 46.0)	(-0.93, 0.23)	8/869	0.92	(-88.0, 67.4)	(-0.99, 0.36)
Misc 1†	Vacc	2/515*	0.39	**75.4**	**6.10**	6/507*	1.18	**25.8**	**5.77**
	Comp	1/64	1.57	(-157.7, 97.7)	(-2.43, 14.62)	1/64	1.57	(-546.0, 91.5)	(-2.77, 14.31)
Misc 2†	Vacc	9/1868*	0.48	**-1.9**	**-0.14**	11/1861*	0.59	**-23.7**	**-0.31**
	Comp	2/378	0.53	(-377.0, 78.2)	(-0.51, 0.24)	2/377	0.53	(-520.4, 75.4)	(-0.76, 0.15)

N, number of cases; PT, person-time at-risk; IR, incidence rate per 100 person-years; VE, vaccine effectiveness; Vacc, Rotarix recipients; Comp, did not receive Rotarix; RD, rate difference = IR in Comp. – IR in Vacc.
* A number of villages did not contribute person-time. Please refer to Table 19 for more details.
† Since these two parameters assess the level of any differences in risk of rotavirus by oral polio virus vaccine status among those who did not receive Rotarix, the definitions for the comparison groups differ from all other effect parameters (see Figure 6).

Table 19. The number of villages that did not contribute person-time of outcome risk, by effect parameter, outcome, and vaccine group.

Vaccine Effect	Age Group	Number of Vaccine : Comparison villages (out of 71 of each type) that did not contribute person-time of risk, by outcome						
		Acute Rotavirus Diarrhea (ARD)	Severe ARD (Vesikari >/=11)	Very Severe ARD (Vesikari >/=15)	G1 or P8 ARD	Non-G1 and Non-P8 ARD	Acute Enterotoxic *E. coli* Diarrhea,	
							<u>Excluding</u> rotavirus co-positive cases	<u>Excluding</u> rotavirus co-positive cases
Overall 1	6-weeks to 2 year	0:00	0:00	0:00	0:00	0:00	0:00	0:00
	6-weeks to 0.9 year	0:00	0:00	0:00	0:00	0:00	0:00	0:00
	1.0 to 1.9 years	1:00	1:00	1:00	1:00	1:00	1:00	1:00
Overall 2	6-weeks to 2 year	0:00	0:00	0:00	0:00	0:00	0:00	0:00
	6-weeks to 0.9 year	0:00	0:00	0:00	0:00	0:00	0:00	0:00
	1.0 to 1.9 years	1:00	1:00	1:00	1:00	1:00	1:00	1:00
Overall 3	6-weeks to 2 year	0:00	0:00	0:00	0:00	0:00	0:00	0:00
	6-weeks to 0.9 year	0:00	0:00	0:00	0:00	0:00	0:00	0:00
	1.0 to 1.9 years	1:00	1:00	1:00	1:00	1:00	1:00	1:00
Total, mITT 1	6-weeks to 2 year	0:00	0:00	0:00	0:00	0:00	0:00	0:00
	6-weeks to 0.9 year	0:00	0:00	0:00	0:00	0:00	0:00	0:00
	1.0 to 1.9 years	1:00	1:00	1:00	1:00	1:00	1:00	1:00
Total, mITT 2	6-weeks to 2 year	0:00	0:00	0:00	0:00	0:00	0:00	0:00
	6-weeks to 0.9 year	0:00	0:00	0:00	0:00	0:00	0:00	0:00
	1.0 to 1.9 years	1:20	1:20	1:18	1:20	1:20	1:17	1:18
Total, mITT 3	6-weeks to 2 year	0:00	0:00	0:00	0:00	0:00	0:00	0:00
	6-weeks to 0.9 year	0:00	0:00	0:00	0:00	0:00	0:00	0:00
	1.0 to 1.9 years	1:00	1:00	1:00	1:00	1:00	1:00	1:00
Total, ATP 1	6-weeks to 2 year	0:00	0:00	0:00	0:00	0:00	0:00	0:00
	6-weeks to 0.9 year	0:00	0:00	0:00	0:00	0:00	0:00	0:00
	1.0 to 1.9 years	1:00	1:00	1:00	1:00	1:00	1:00	1:00
Total, ATP 2	6-weeks to 2 year	0:05	0:05	0:05	0:05	0:05	0:05	0:05
	6-weeks to 0.9 year	0:05	0:05	0:05	0:05	0:05	0:05	0:05
	1.0 to 1.9 years	1:20	1:20	1:18	1:20	1:20	1:17	1:18
Total, ATP 3	6-weeks to 2 year	0:00	0:00	0:00	0:00	0:00	0:00	0:00
	6-weeks to 0.9 year	0:00	0:00	0:00	0:00	0:00	0:00	0:00
	1.0 to 1.9 years	1:00	1:00	1:00	1:00	1:00	1:00	1:00
Indirect 1	6-weeks to 2 year	0:00	0:00	0:00	0:00	0:00	0:00	0:00
	6-weeks to 0.9 year	0:00	0:00	0:00	0:00	0:00	0:00	0:00
	1.0 to 1.9 years	0:00	0:00	0:00	0:00	0:00	0:00	0:00
Indirect 2	6-weeks to 2 year	0:00	0:00	0:00	0:00	0:00	0:00	0:00
	6-weeks to 0.9 year	0:00	0:00	0:00	0:00	0:00	0:00	0:00
	1.0 to 1.9 years	4:07	4:07	4:05	4:07	4:07	4:05	4:06
Direct, mITT 1	6-weeks to 2 year	0:00	0:00	0:00	0:00	0:00	0:00	0:00
	6-weeks to 0.9 year	0:00	0:00	0:00	0:00	0:00	0:00	0:00
	1.0 to 1.9 years	1:01	1:01	1:01	1:01	1:01	1:01	1:01
Direct, mITT 2	6-weeks to 2 year	0:00	0:00	0:00	0:00	0:00	0:00	0:00
	6-weeks to 0.9 year	0:00	0:00	0:00	0:00	0:00	0:00	0:00
	1.0 to 1.9 years	1:20	1:20	1:17	1:20	1:20	1:17	1:17
Direct, mITT 3	6-weeks to 2 year	0:00	0:00	0:00	0:00	0:00	0:00	0:00

Vaccine Effect	Age Group	Number of Vaccine : Comparison villages (out of 71 of each type) that did not contribute person-time of risk, by outcome						
		**Acute Rotavirus Diarrhea (ARD)**	**Severe ARD (Vesikari >/=11)**	**Very Severe ARD (Vesikari >/=15)**	**G1 or P8 ARD**	**Non-G1 and Non-P8 ARD**	**Acute Enterotoxic *E. coli* Diarrhea,**	
							**Excluding rotavirus co-positive cases**	**Excluding rotavirus co-positive cases**
	6-weeks to 0.9 year	0:00	0:00	0:00	0:00	0:00	0:00	0:00
	1.0 to 1.9 years	1:01	1:01	1:01	1:01	1:01	1:01	1:01
Direct, ATP 1	6-weeks to 2 year	0:00	0:00	0:00	0:00	0:00	0:00	0:00
	6-weeks to 0.9 year	0:00	0:00	0:00	0:00	0:00	0:00	0:00
	1.0 to 1.9 years	1:01	1:01	1:01	1:01	1:01	1:01	1:01
Direct, ATP 2	6-weeks to 2 year	0:09	0:09	0:09	0:09	0:09	0:09	0:09
	6-weeks to 0.9 year	0:09	0:09	0:09	0:09	0:09	0:09	0:09
	1.0 to 1.9 years	1:20	1:20	1:17	1:20	1:20	1:17	1:17
Direct, ATP 3	6-weeks to 2 year	0:01	0:01	0:01	0:01	0:01	0:01	0:01
	6-weeks to 0.9 year	0:01	0:01	0:01	0:01	0:01	0:01	0:01
	1.0 to 1.9 years	1:01	1:01	1:01	1:01	1:01	1:01	1:01
Miscellaneous 1	6-weeks to 2 year	1:09	1:09	1:09	1:09	1:09	1:09	1:09
	6-weeks to 0.9 year	1:09	1:09	1:09	1:09	1:09	1:09	1:09
	1.0 to 1.9 years	1:20	1:20	1:17	1:20	1:20	1:17	1:17
Miscellaneous 2	6-weeks to 2 year	0:00	0:00	0:00	0:00	0:00	0:00	0:00
	6-weeks to 0.9 year	0:00	0:00	0:00	0:00	0:00	0:00	0:00
	1.0 to 1.9 years	0:08	0:08	0:06	0:08	0:08	0:06	0:07

Table 20. For the population contributing to the Overall Effectiveness 1 analysis, the number (N=99) of participants in HRV villages who received at least one HRV dose off-protocol, i.e., HRV 1 with OPV2 and/or HRV2 with OPV3. The table also summarizes the number of days that elapsed from when the associated HRV dose should have been administered (per protocol) to when the dose was actually received.

Combinations of HRV-OPV dose mismatched	Number of Participants	Number of days between the True Administration Date and the Prescribed Administration Date									
		OPV2 versus OPV1					OPV3 versus OPV2				
		Mean	Standard Deviation	Median	Minimum	Maximum	Mean	Standard Deviation	Median	Minimum	Maximum
HRV1+OPV2	12	37	9	36	29	57					
HRV1+OPV2, HRV2+OPV3	75	40	11	36	29	79	32	3	31	29	49
HRV2+OPV3	12						39	11	36	29	65

Figure 7. By quarter, the number (dashed line) of single-strain infections (G and P serotype typed and only one of each reported [N=466]) detected among study participants (*i.e.*, eligible to contribute to the estimation of at least one of the human rotavirus vaccine, Rotarix®, effectiveness parameter), as well as the quarterly prevalence of each combination of G and P serotypes (color-shaded regions). The strain prevalence estimates are stacked, so that the width of each color band represents the proportion of single strain infections reporting the associated G-P combination.

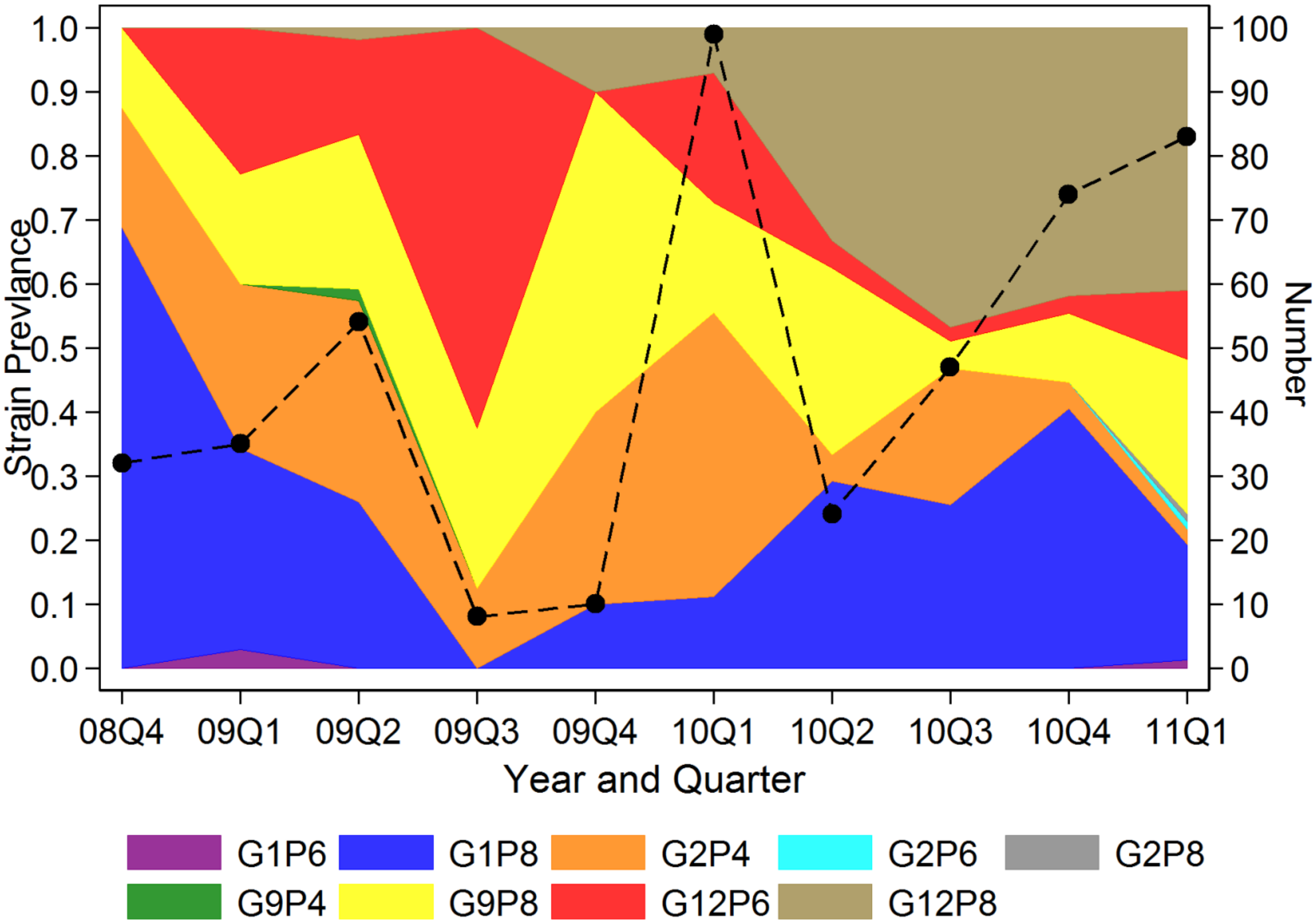

Supplement: S5 Text — (PDF) [file pmed.1002282.s005.pdf]
