# Supplementary material for: Effectiveness of a live oral human rotavirus vaccine after programmatic introduction in Bangladesh: A cluster-randomized trial
Source: PLoS Med. 2017 Apr 18;14(4):e1002282. doi: 10.1371/journal.pmed.1002282 (PMC5395158; doi:10.1371/journal.pmed.1002282)
Supplement: S2 Data — (DOCX) [file pmed.1002282.s008.docx]

**Table. Description of the contents of the trial dataset**

| **Column Index** | **Column Name** | **Description** | **Possible Values** |
| --- | --- | --- | --- |
| 1 | service_area | Indicator for the icddr,b service area. Clusters were randomized within these service areas. | 0=government service area  1=icddr,b service area |
| 2 | cluster_masked | Masked cluster identification number | government service area: 1-75  icddr,b service area: 1-67 |
| 3 | masked_individual_id | Masked unique individual identifier | government service area: 1-6389  icddr,b service area: 1-5928 |
| 4 | cluster_intervention_assignment | Cluster-level intervention assignment | 0=Standard EPI  1=Standard EPI + Rotarix |
| 5 | age_at_enrollment | Age (days) at enrollment | >=42 |
| 6 | age_at_1st_exit_from_study_area | Age (days) at earliest of first emigration out of the study area, 2nd year birthday, or end of study. |  |
| 7 | age_at_re_entry_to_study_area | Age (days) at first post-enrollment immigration back into the study area |  |
| 8 | age_at_2nd_exit_from_study_area | Age (days) at earliest of second emigration out of the study area, 2nd year birthday, or end of study. (missing if 1^st^ emigration never occurred prior to 2^nd^ birthday or end of study) |  |
| 9 | age_at_rotarix_dose_1 | Age (days) of receipt of Rotarix dose 1 for residents of clusters with cluster_intervention_assignment=1 |  |
| 10 | age_at_rotarix_dose_2 | Age (days) of receipt of Rotarix dose 2 for residents of clusters with cluster_intervention_assignment=1 |  |
| 11 | age_at_opv_dose_1 | Age (days) of receipt of post-birth dose 1 of the oral polio vaccine |  |
| 12 | age_at_opv_dose_2 | Age (days) of receipt of post-birth dose 2 of the oral polio vaccine |  |
| 13 | age_at_opv_dose_3 | Age (days) of receipt of post-birth dose 3 of the oral polio vaccine |  |
|  | First post-enrollment acute rotavirus (rv_1st_ prefix) and enterotoxic *E. coli* (etec_1st_ prefix) Diarrhea Episodes |  |  |
| 14/29 | age_at_admission | Age (days) at admission to study surveillance facility |  |
| 15/30 | in_study_area | Did the episode occur while child was resident in the study area? | 0=No  1=Yes |
| 16/31 | hosp_required | Did the visit to the hospital meet the criteria for hospitalization? (overnight stay/admission) | 0=No  1=Yes |
| 17/32 | only_oral_rehyd_admin | Was the patient only treated with oral rehydration during the diarrheal episode? | 0=No  1=Yes |
| 18/33 | num_diar_in_24_hrs | During the diarrheal episode, the maximum number of looser than normal stools during a 24-hour period |  |
| 19/34 | num_vomitting_in_24_hrs | During the diarrheal episode, the maximum number of episodes of vomiting during a 24-hour period |  |
| 20/35 | level_of_dehydration | Clinically-assessed level of dehydration | 1=None  2=Some  3=Severe |
| 21/36 | highest_temp_in_24_hrs | Highest temperature (rectal equivalent) recorded during the diarrheal episode (degrees Celsius) |  |
| 22/37 | duration_diarrhea | Duration (days) of looser than normal stools |  |
| 23/38 | duration_vomitting | Duration (days) of vomiting during the diarrheal episode |  |
| 24/39 | modified_vesikari_score | Modified Vesikari Score (based upon preceding 8 variables) | 0-20 |
|  | Pathogen-specific information about diarrheal episodes |  |  |
| 25 | rv_1st_age_at_spec_coll | First acute rotavirus diarrhea episode: age (days) when clinical specimen was collected for ELISA and G and P typing laboratory tests. |  |
| 26 | rv_1st_elisa_result | First acute rotavirus diarrhea episode: Positive ELISA laboratory test result indicator | 1=Positive |
| 27 | rv_1st_g_type | First acute rotavirus diarrhea episode: G typing result | 0 (undetermined),1,2,9,12,M (multiple G types detected in the same specimen) |
| 28 | rv_1st_p_type | First acute rotavirus diarrhea episode: G typing result | 0 (undetermined),4,6,8,M (multiple P types detected in the same specimen) |
| 40 | etec_1st_rotavirus_coinf | First acute enterotoxic *E. coli* diarrheal episode: Indicator for whether or not rotavirus coinfection was detected via ELISA. | 0=No  1=Yes |
